# Optimization of the genetic code expansion technology for intracellular labelling and single-molecule tracking of proteins in genomically re-coded *E. coli*

**DOI:** 10.1039/d5cb00221d

**Published:** 2025-11-24

**Authors:** Filip Ilievski, Linnea Wikström, Anneli Borg, Ivan L. Volkov, Gerrit Brandis, Magnus Johansson

**Affiliations:** a Department of Cell and Molecular Biology, Uppsala University Uppsala Sweden m.johansson@icm.uu.se; b Uppsala Antibiotic Center, Uppsala University Uppsala Sweden

## Abstract

Single-molecule tracking (SMT) is a powerful tool for real-time studies of protein interactions in living cells. Dye-labelled SNAP-tag and HaloTag self-labelling proteins have simplified SMT significantly, due to their superior photophysical properties compared to fluorescent proteins. However, due to their size, fusion of these tags to a protein of interest often results in loss of protein function. We introduce FLORENCE – a universal labelling method for SMT, based on genetic code expansion (GCE). We overcome significant caveats related to re-coded strains, vectors, and dyes and report successful tracking of site-specifically intracellularly labelled proteins in genomically re-coded *E. coli.* Our findings establish a robust *in vivo* protein-labelling strategy, expanding the capabilities of SMT as a method to study the dynamics of proteins in living cells. Moreover, we observe that the strain-promoted azide–alkyne click-chemistry reaction occurs as fast as 30 min in live *E. coli* cells and can be used as a robust labelling reaction.

## Abstract 2

Genetic code expansion (GCE) technology has been optimized, applied, and validated for single-molecule tracking (SMT) of intracellularly dye-labelled proteins in a fully re-coded *E. coli* strain.

## Graphical abstract

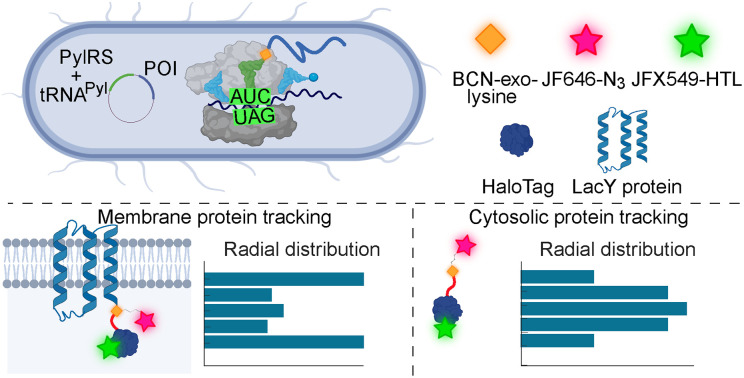

## Introduction

Single-molecule tracking (SMT) has in recent years been successfully used to study intermolecular dynamics directly inside living cells.^1–4^ A prerequisite of the method, however, is that the protein of interest (POI) can be labeled with a fluorophore, typically *via* genetic fusion to a fluorescent protein^5^ or self-labeling tag,^2^ or by *in vitro* covalent labeling with subsequent cell re-internalization by electroporation.^6–8^ Although historically successful, the protein fusion approach is restricted to POIs that can tolerate the attachment of a 20–30 kDa protein moiety at their N- or C-terminus without significant function disruption. *In vitro* labeling of the POI with small, photostable organic dyes provides better opportunities for labeling without compromising the function of the POI and extends the flexibility regarding the label position. So far, however, this approach has primarily been used for electroporation and tracking of nucleic acids,^6–8^ which have proven more stable during the electroporation process, with only a few proof-of-concept studies of electroporated proteins.^9^ A universal labeling approach combining the flexibility of site-specific incorporation of small organic dyes into the POI with the robustness and convenience of genetically encoded fluorescence labeling would, thus, provide new opportunities for live-cell biochemical studies using SMT.

Genetic code expansion (GCE) is, to date, the only technology for site-specific co-translational insertion of designer chemical entities into proteins that offers almost complete flexibility regarding the position in the amino acid sequence.^10^ The most widely adopted approach thus far is by stop codon UAG re-assignment, pioneered by the Schultz group^11^ and expanded to other model organisms by others.^12^ Hitherto, GCE, in *E. coli*, has been successfully used to, *e.g.*, expand the chemical space of protein therapeutics, identify protein–protein interactions by *in vitro* photo-crosslinking, and produce antibody–drug conjugates.^10,13,14^ However, the application of this system to directly study protein dynamics inside living *E. coli* cells remains underexplored. Although Kipper *et al.*^15^ successfully click-labeled and tracked the outer membrane protein OmpC, their approach did not allow for intracellular protein labeling, mainly due to the unspecific retention of dyes inside the cells, masking the signal of the protein of interest. Moreover, when GCE was applied in release factor 1 (RF1)-containing strains, significant toxicity was observed due to the presence of truncated proteins. Subsequent to the work of Kipper *et al.*,^15^ a number of improvements have been made in each of the necessary components of a GCE system for SMT. For example, the synthesis of the bright, cell-permeable, and easily washable Janelia Fluor (JF) series of dyes has simplified *in vivo* single-molecule fluorescence studies tremendously,^2,16^ and further, re-coded *E. coli* strains based on the C321 lineage have been extensively engineered and adapted for more efficient protein production.^17–19^ However, the reported re-coded strains are not suitable for direct biological studies, as they suffer from physiological defects resulting from the hypermutator background used for construction.^20^ Thus far, there have not been any reports on the rational generation of a model re-coded *E. coli* suitable for biological studies by SMT and live-cell microscopy experiments, based on the GCE technology.

**Fig. 1:**
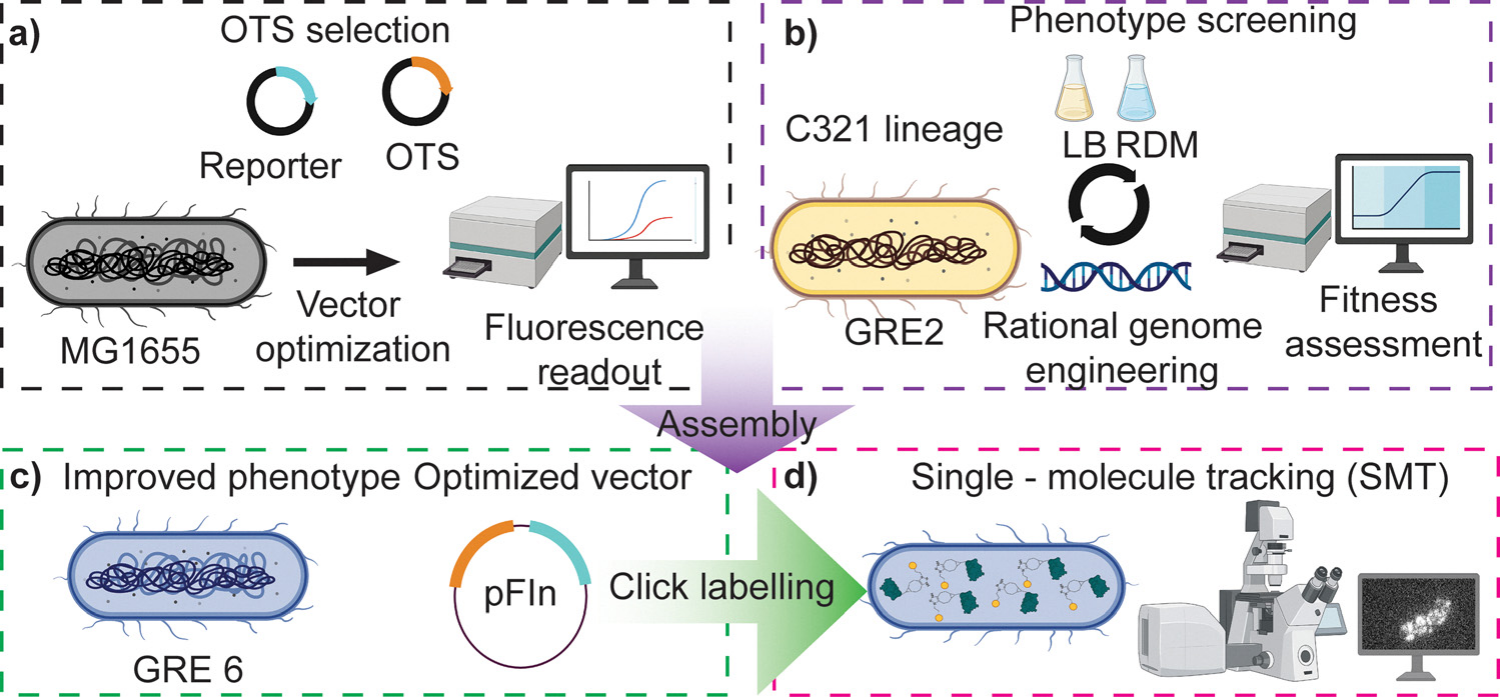
Schematic representation of FLORENCE development. (a) The workflow started with screening for optimal OTS (PylRS/tRNA_CUA_ pairs) engineered for clickable ncAA by monitoring the expression of a fluorescent reporter in the commonly used lab strain MG1655. (b) In parallel, a GRE was rationally engineered for fitness improvements and physiological temperature growth in LB and RDM (Rich Defined Media, 0.2% glucose). (c) Within the improved working phenotype of the GRE, the expression vector (pFIn) was then optimized. (d) SMT of labeled proteins validated the presented approach.

The aim of this work was to engineer a minimal, rapid, robust, and general system for site-specific, bio-orthogonal dye-labeling of proteins *in vivo*, for biological studies using SMT. We developed such a system by evaluating the most commonly used, commercially accessible cell-permeable non-canonical amino acids (ncAAs), dyes, newly engineered orthogonal translation systems (OTS), and genomically re-coded *E. coli* strains (GREs) (Fig. 1). We rationalize the usage of a GRE with the fact that (i) a UAG-free strain allows single-codon resolution labeling of only a specific, well-defined protein, without any off-target labeling of abundant intracellular proteins terminating with endogenous UAG codons; (ii) RF1-deficient strains significantly reduce the production of truncated proteins,^21^ which otherwise might interfere with the cell function.^1^ We term the system FLORENCE (Fluorescence labelling in re-coded *E. coli* with non-canonical chemical entities) and benchmark it, using SMT of intracellularly labeled proteins in living bacterial cells. Our data obtained from direct comparisons between HaloTag-ligand (HTL) and click-labelled proteins validate FLORENCE as a robust labeling method for SMT.

## Results

### Selection of PylRS for clickable ncAA incorporation

**Fig. 2:**
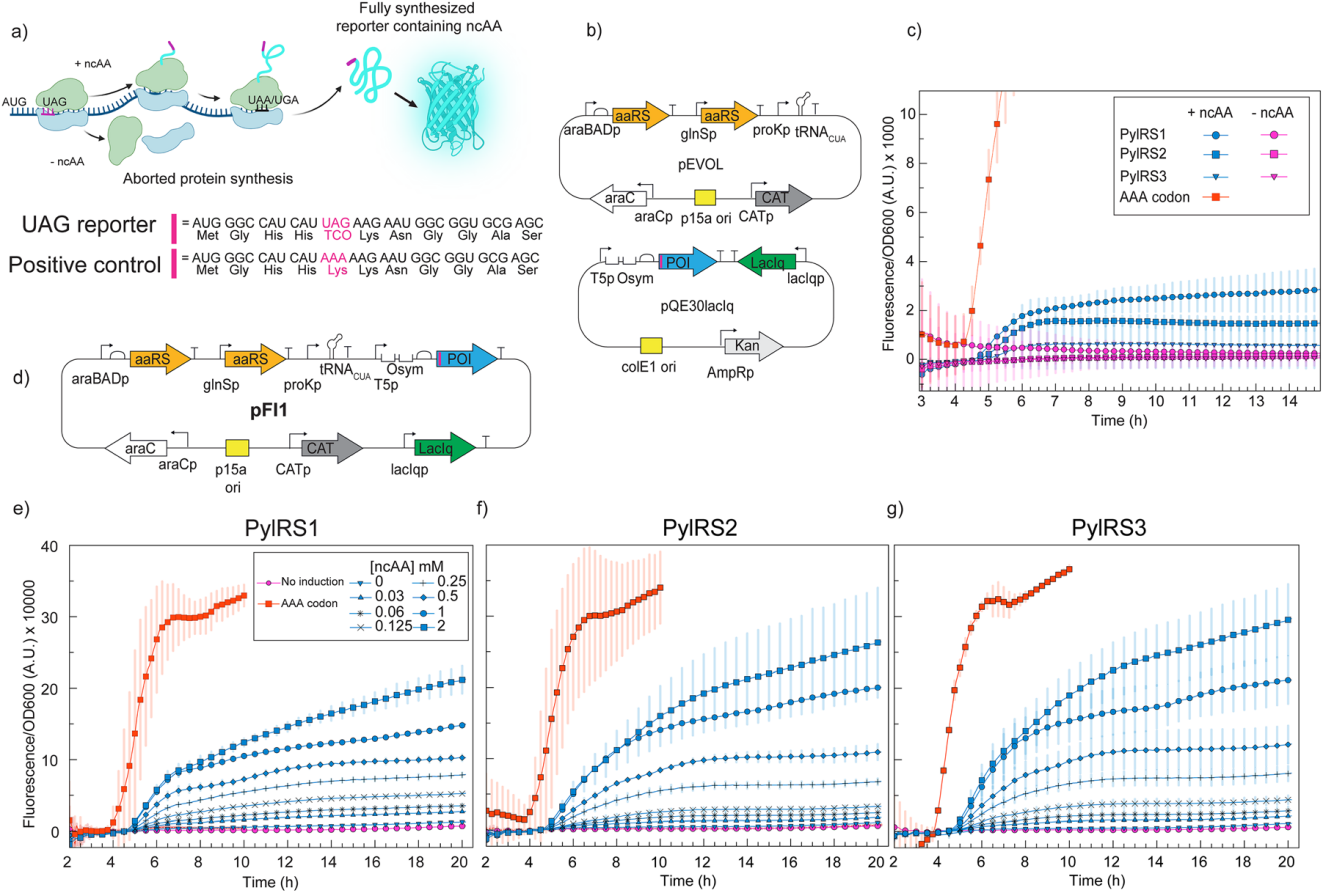
(a) Schematic representation of the fluorescence reporter assay. The reporter protein, SCFP3a, contains an N-terminally fused codon-context linker with the UAG codon. In the absence of ncAA, expression of the reporter is suppressed. (b) Schematic representation of the two-plasmid system, where the reporter protein is under the control of the LacIq repressor and the OTS is on the pEVOL vector. (c) Reporter fluorescence signal normalized to cell density (OD_600_) for the three PylRSs tested, using the two-plasmid system. The experiments were performed in LB media in the presence of 1 mM TCO*AK, with co-induction of the OTS with 0.02% arabinose and 1 mM IPTG of the reporter. A UAG-to-AAA mutation in a separate, identical construct is used as a positive control. (d) Schematic representation of the single plasmid system pFI1, where the reporter protein SCFP3a with *lacIq* was cloned onto the pEVOL vector. (e)–(g) Reporter fluorescence signal normalized to cell density (OD_600_) for the three PylRSs tested, using the one-plasmid system. The experiments were performed in the presence of increasing concentrations of TCO*AK, with co-induction of the OTS with 0.02% arabinose and 1 mM IPTG of the reporter. A UAG-to-AAA mutation in a separate, identical construct is used as a positive control. Data are representative of three independent replicates.

We started by adopting the pEVOL-based^22^ OTS system^23^ from Kipper *et al.*,^15^ containing the *Methanoscarnia mazei* pyrrolysyl-tRNA synthetase (PylRS1), which has been rationally engineered to incorporate bulky click-amino acids.^24^ The pEVOL vector harbors two copies of the PylRS – one constitutively expressed (*glns* promoter) and one under the control of the *araBAD* promoter. In addition, the plasmid contains a constitutively expressed native tRNA^Pyl^ gene from *M. mazei* under the native *proK* promoter. In the study, we also included the latest engineered versions of *Methanomethylophilus alvus* PylRS (PylRS2 and PylRS3) with reported high *in vitro* aminoacylation activity of clickable ncAAs.^25^ The advantage of the *M. alvus* PylRSs is their reported higher solubility compared to other PylRSs,^25^ which makes them more attractive if high levels of the PylRS expression are needed. Corresponding pEVOL plasmids with the two PylRS1 copies replaced with either PylRS2 or PylRS3 were, hence, also constructed. To investigate the efficiency of ncAA incorporation in the presence of either of the PylRS variants, we utilized an IPTG inducible reporter system (*lac O*_sym_ promoter), where a UAG codon, along with a 10 amino acid-long codon-context linker, had been inserted at the N-terminus of the SCFP3a fluorescent protein gene (Fig. 2a). Such an optimized codon-context linker has previously been reported to improve the UAG reading efficiency.^26^ During these initial tests of the OTS and expression system, we used the clickable ncAA TCO*AK (ncAA2, Fig. 5a).^15^ Our single-molecule tracking experiments^27^ are typically performed in Rich Defined Media (RDM), due to lower autofluorescence compared to, *e.g.*, LB. However, since our RDM contains 0.2% glucose, and glucose might interfere with protein expression from the *lac* promoter (*i.e.*, inactive CRP^28^), we chose to perform all optimization experiments in LB. In the final microscopy experiments, the ncAA-containing protein was also expressed in LB, and the growth medium was switched to RDM prior to cell preparation for microscopy (Fig. 6 and Methods).

The reporter protein SCFP3a^29^ was first expressed in the commonly used *E. coli* lab strain MG1655 from a separate pQE30 high-copy-number plasmid under the control of the IPTG-inducible T5-lac hybrid promoter (Fig. 2b). We found no significant differences in the reporter production signal (fluorescence normalized to OD_600_) for the three PylRSs tested (Fig. 2c). However, the reporter protein production is in all three cases significantly lower than that from an identical pQE30 reporter plasmid with the position 5 UAG codon replaced by a canonical AAA lysine codon (Fig. 2c). Low efficiency of ncAA incorporation has been reported previously^22,26,30^ and is considered one of the major bottlenecks of the approach. It should be noted, however, that our fluorescence-based assay does not necessarily represent the absolute protein yields. Changing the AAA Lys5 codon to the UAG ncAA codon might also affect the folding and maturation of the reporter protein – something that potentially could be detected by quantifying protein yields using, *e.g.*, western blotting. The observation that a higher reporter signal can be reached using a different plasmid construct (Fig. 2e and Fig. S1), though, suggests that such potential folding and maturation is not the only reason for a lower signal compared to the AAA-codon containing positive control.

In an attempt to decrease the number of variables in the systems’ construction, decrease variability due to the plasmid copy number, and decrease the plasmid copy number for expressing potentially toxic proteins during SMT, we generated a one-plasmid system with the reporter protein along with its regulatory components cloned onto the pEVOL vector, keeping the cassette architecture from the two-plasmid system (Fig. 2b). In this one-plasmid system (pFI1, Fig. 2d), the reporter signal is markedly higher than that in the two-plasmid system, independent of the PylRS variant used (Fig. 2e–g). Titration of the ncAA suggests saturation at around 1 mM in all three cases. Further, to test whether the position of the reporter on the vector could influence the expression efficiency, we also placed the reporter in a different region on the pEVOL vector (pFI2), annotated not to contain any coding or regulatory sequences. We saw a notable decrease in fluorescence (Fig. S1), suggesting that, indeed, for a single-plasmid system, the expression of the gene is position-dependent, relative to other genes on a given plasmid, as recently reported.^31^ Based on the slightly higher reporter signal at higher ncAA concentration (Fig. 2e–g), we decided to pursue the study using the PylRS3 variant on the first one-plasmid system tested (pFI1), where the POI gene is positioned directly downstream of the *proK* terminator of the tRNA^Pyl^.

### Rational engineering of the C321 lineage improves fitness and allows growth in imaging media at 37 °C

**Fig. 3:**
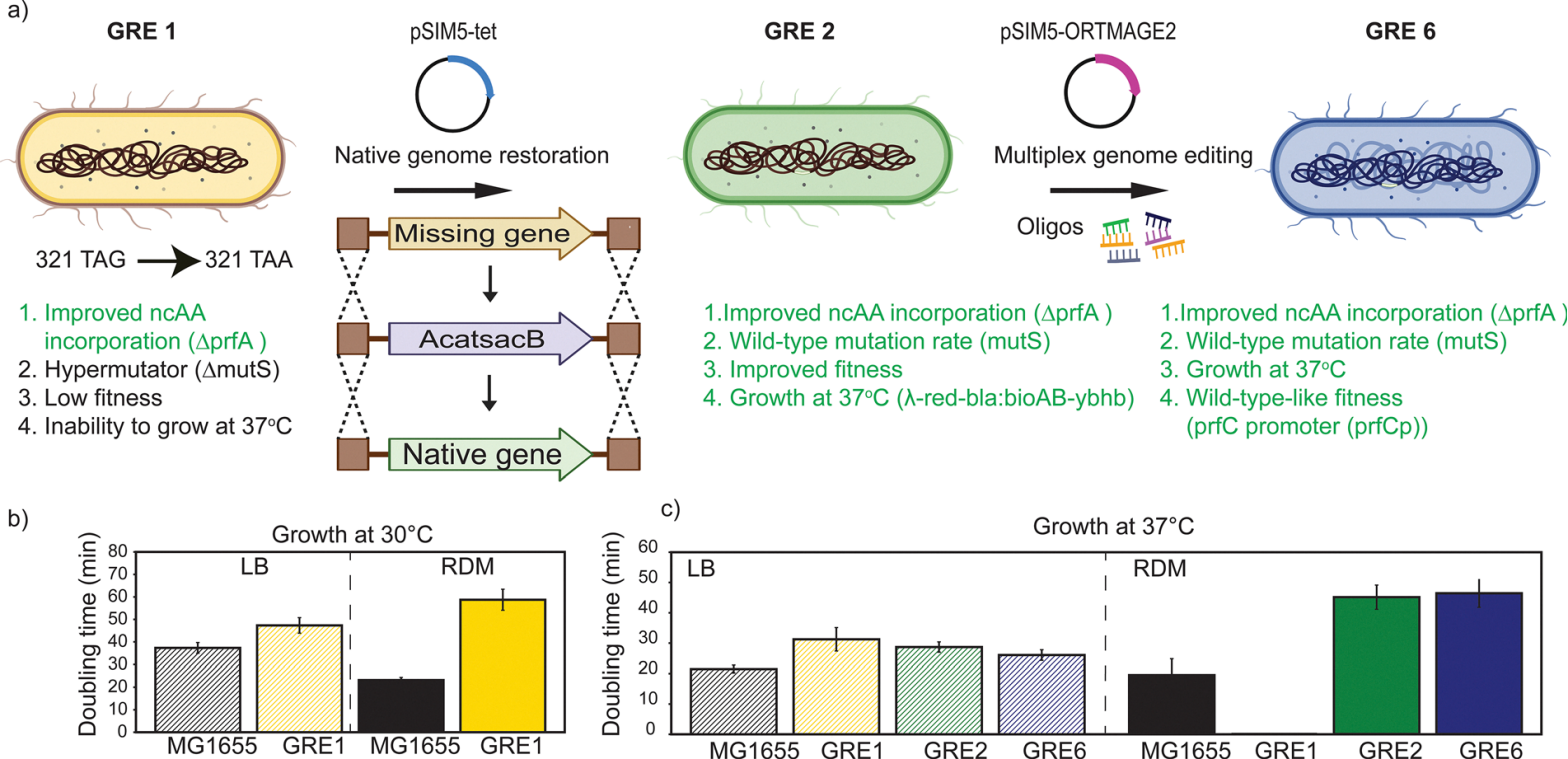
(a) Rational engineering of C321 lineage for SMT to restore a near-MG1655-like genotype and phenotype. Missing genes at native loci were reinstalled by integrating the *CatSacB* cassette for selection/counterselection. Multiplex gene editing (MGE) was subsequently applied to revert the MAGE and adaptive lab evolution-acquired single-point mutations targeting multiple loci, using a newly constructed pSIM5-ORTMAGE2 plasmid.^2^ (b) and (c) Characterization of C321.ΔA.M9adapted (GRE1). The data are representative of three independent experiment replicates.

In order to generate a GRE suitable for SMT, we first screened the most recent community-available strains from the C321 lineage^20^ based on their fitness in the transparent low-fluorescent Rich Defined Medium (RDM 0.2% glucose) used in our single-molecule tracking experiments.^2^ The C321 lineage is a *tour de force* of the Church lab, where the lab strain MG1655 underwent complete replacement of all annotated UAG to UAA codons. This strain also lacks RF1 (Δ*prf*A), which increases ncAA incorporation efficiency and prevents formation of truncated products.^21^GRE1 (C321.ΔA.M9.adapted)^19^ is a minimal-medium adapted strain of the C321 lineage,^18^ with reported improved fitness after laboratory evolution (ALE) in all tested media.^19^ The fitness improvement in GRE1 has been mainly attributed to a hitchhiker mutation in the *folA* promoter acquired during the ALE of the parent strain, and the *prfB* (RF2) T246A mutation, characteristic of the B lineage of *E. coli* strains. Interestingly, Hemez *et al.*^32^ reported a 50% decrease in the doubling time after introducing the T246A mutation in the *prfB* gene in the ancestral C321.^20^ In our case, GRE1 displayed a doubling time of 60 min in RDM at 30 °C (Fig. 3b) and an inability to grow at 37 °C in the same medium. As RDM is the optimal low-fluorescent imaging medium on which all our studies are based,^2,7,8,33^ we sought to optimize the growth of GRE1 in RDM (Fig. 3a). We performed rational genome engineering of GRE1 by restoring the native *bioAB-ybhb* locus, originally replaced with the lambda red prophage system for multiplex automated genome editing (MAGE^20^), to enable growth at 37 °C. We also restored *mutS* (mismatch DNA repair protein) and *tolC* (efflux transporter), to obtain a stable genotype^34^ and decrease cell autofluorescence,^35^ respectively. This round of engineering generated GRE2, which grows both in LB and RDM at 37 °C (Fig. 3c). To further investigate if the fitness of GRE2 could be improved in RDM, we removed the frameshift in *rph*, *flgE*, and *ybhb* (GRE3), using multiplex genome editing (MGE). We reverted the mutations in the *ftsA* and *yeeJ* loci back to wild-type, as reported by Thyer *et al.*^36^ (GRE4). We subsequently removed the frameshift mutation in the *ilvG* gene and reverted the single-point mutations in *leuS*, *bamA*, and *gpp* as reported by Hemez *et al.*^32^ (GRE5). Finally, we restored the −10 box of the native *prfC* promoter (GRE6) (Fig. 3c). We, however, did not find any significant improvement in the growth rate of GRE6 compared to GRE3–GRE5 in RDM at 37 °C (Fig. S2). In LB media at 37 °C, there was a slight improvement in the growth of GRE6 compared to GRE1, and in particular, the optimized GRE6 strain displays much more homogenous cell morphology relative to the ancestral GRE1 strain when grown on a mother machine microfluidic device (Fig. S3).^37^ However, single-cell analysis in the growth rate of GRE2 and GRE6 in both RDM and LB media did not show a significant difference (Fig. S4 and S5). Based on its’ genomic similarity to MG1655, we present the final engineered strain GRE6 as a model organism suitable for SMT using GCE and click-chemistry labelling.

### ncAA incorporation in GRE6 requires constitutive expression of aaRS

**Fig. 4:**
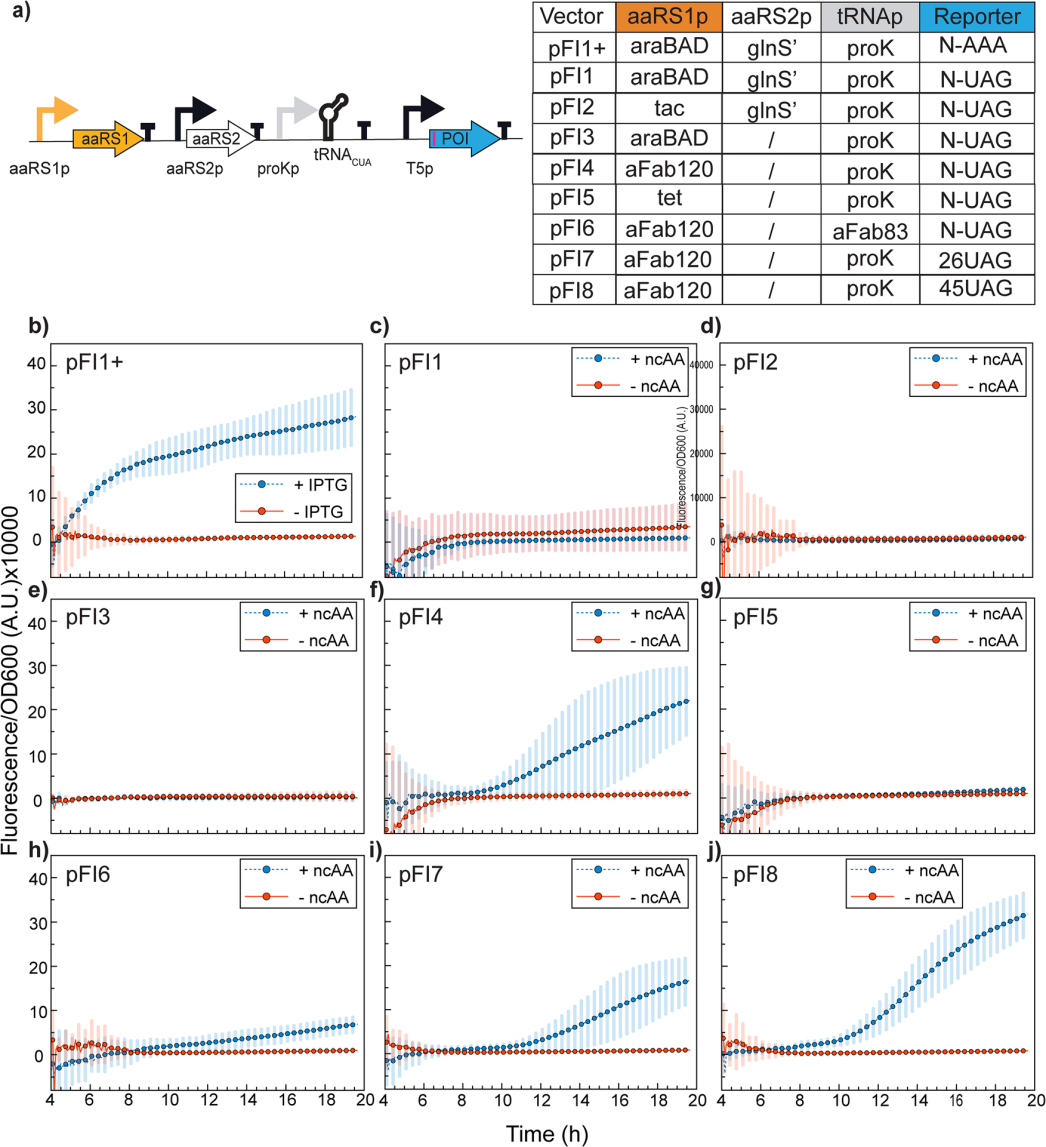
Vector optimization for aaRS expression in GRE6 in LB media. (a) Schematic representation of the constructs’ backbone and all construct variants tested. pFI1+ is included as a positive control for SCFP3a expression, with the AAA codon instead of UAG at the same position. (b)–(j) Reporter fluorescence signal normalized to cell density (OD_600_) for all one-plasmid constructs tested. The experiments were performed in the absence (−ncAA) or presence (+ncAA) of 1 mM TCO*AK, with co-induction of the OTS with 0.02% arabinose for pFI1, pFI1+, and pFI3, 0.2% anhydrotetracycline for pFI5 and 1 mM IPTG of the reporter in all constructs and the OTS in pFI2. All experiments were performed in LB media. Data are representative of *n* = 3 independent experiments.

When the one-plasmid OTS and the reporter system (PylRS3 and pFI1, Fig. 4a) were inserted and induced in our optimized GRE6, we observed no reporter protein expression (Fig. 4c). We hypothesized that some of the remaining mutations in our optimized GRE6 might affect the arabinose metabolism or arabinose-dependent transcription regulation. In fact, Yi *et al.*^38^ reported downregulation of the arabinose transporter and different metabolic re-wiring of the parental C321 compared to MG1655. To address this hypothetical problem, we generated constructs with a single aaRS copy under the control of the inducible *tac* promoter^39^ (pFI2, Fig. 4d), the inducible *tet* promoter^32^ (pFI5, Fig. 4g), or with a constitutive promoter (pFI4, Fig. 4f). For the latter, we chose *apFAB120*,^40^ which has been demonstrated to work reliably and without any significant fitness cost *in vivo.*^41^ In all of these constructs, we retained the native *proK* promoter of the tRNA.

We did not find any activity of PylRS3 under inducible conditions, neither with IPTG in pFI2 nor with anhydrotetracycline in pFI5. It should also be noted that a single PylRS copy plasmid, with arabinose-induced expression, did not yield any significant reporter signal (pFI3, Fig. 4e). Contrary to this, the construct with a single copy, constitutively expressed PylRS3 (pFI4, Fig. 4f), resulted in a clear reporter signal. In a recent report, the Ellington lab,^42^ switched their induction system from arabinose to IPTG while evolving translation elongation factor SelB in a C321 strain, indicating that arabinose induction could be problematic in this lineage, in line with our results. Moreover, we observed decreased ncAA incorporation efficiency by changing the tRNA promoter from *proK* to *aFab83* in the pFI4 vector (pFI6, Fig. 4h). *aFab83* and *aFab120* were reported to have the same promoter strength;^40^ however, the strength of *proK* remains unknown. Overall, these results demonstrate that for GRE6, without fully resolved MAGE-acquired metabolic defects, constitutive expression of PylRS3 from an *aFAB120* promoter provides optimal expression of protein production, in line with the findings of Seitchik *et al*.^43^ To understand whether this is due to metabolic defects or any other defects in gene regulation mechanisms, further studies will be needed.

Finally, since our ultimate aim was to generate a system for site-specific click-labeling of proteins with minimal disturbance to the native structure and function, we also generated constructs in which the UAG codon was moved into the reporter gene (positions 26 and 45) without the surrounding codon-context linker. We selected the positions based on the Consurf database,^44^ a curated repository with conservation-based sequence analysis, predicting single-site mutations permissible for mutagenesis. From these results, we found that the codon-context linker has little if any influence on reporter protein production from our construct (pFI4, pFI7, pFI8, Fig. 4f, i and j) and that the positions 26 and 45 on the SCFP3a are indeed tolerable for ncAA incorporation.

### Screening of clickable ncAAs and dyes for SMT

**Fig. 5:**
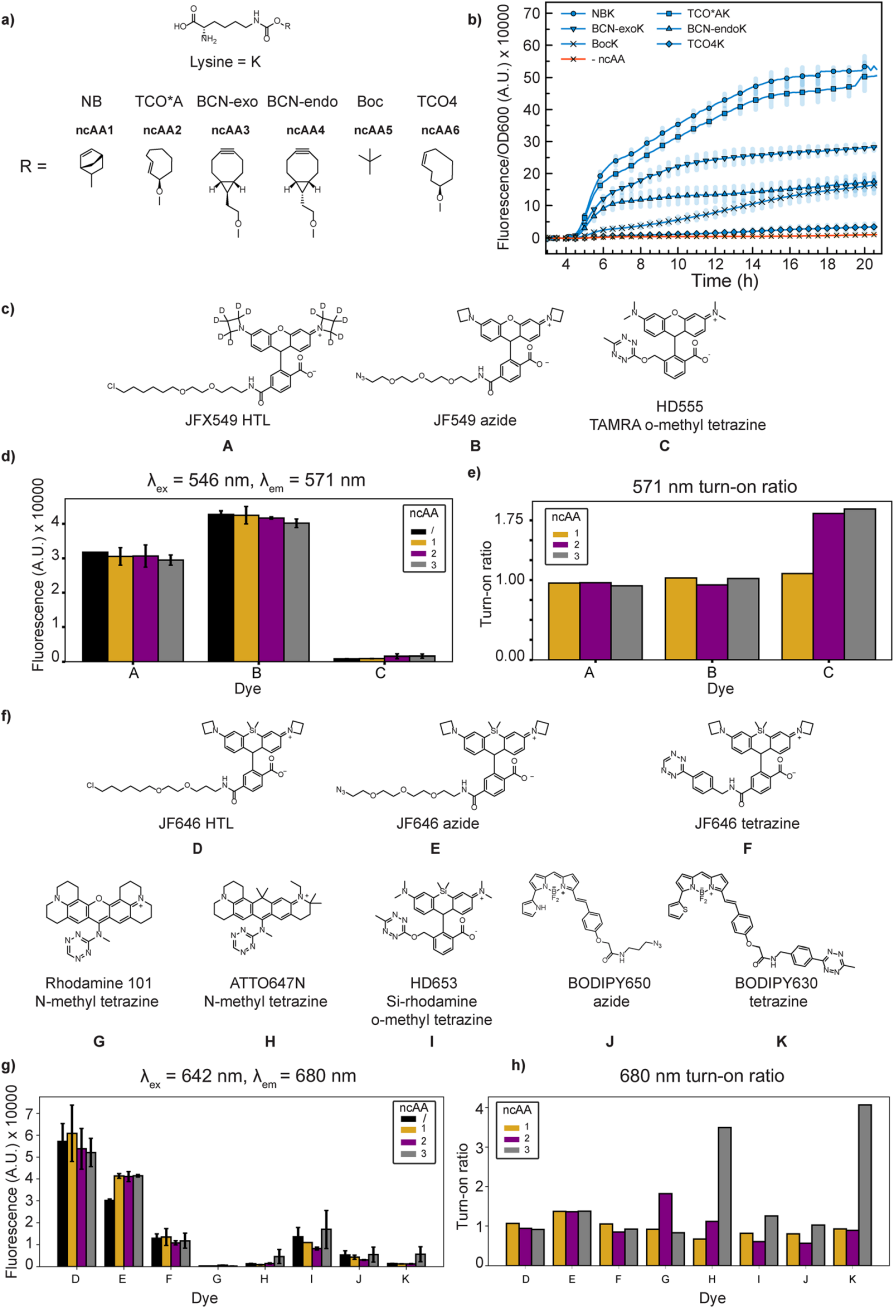
(a) Structure of clickable amino acids assayed. (b) Incorporation efficiency of amino acids in GRE6 using pFI4. (c) Structures of 546 nm excitable dyes. (d) Fluorescence and (e) turn on ratios of 546 nm excited dyes, measured at 1 µM with or without the addition of 50 µM clickable amino acid. (f) Structures of 642 nm excitable dyes. (g) Fluorescence and (h) turn on ratios of 642 nm excited dyes, measured at 1 µM with or without the addition of 50 µM clickable amino acids. The data are a representation of two independent experimental replicates.

A number of clickable ncAAs for protein labeling have been reported, with varying incorporation efficiency depending on both the efficiency of the tRNA aminoacylation reaction,^25^ delivery by EF-Tu,^45^ and the actual incorporation into polypeptides on the ribosome.^46,47^ Furthermore, the structure of the clickable group has been reported to influence the photophysical properties of the dyes in the final click-product.^48^ Based on those observations, we designed two sets of experiments. We first used the pFI4 construct (Fig. 4f) to test PylRS3's ability to incorporate commercially available clickable ncAAs norbornene-lysine (ncAA1, NBK), a *trans*-cyclo-2-octene lysine axial isomer (ncAA2, TCO*AK), a bicyclo [6.1.0] nonyne-lysine exo diastereoisomer (ncAA3, BCN-exoK), a bicyclo [6.1.0] nonyne-lysine endo diastereoisomer (ncAA4, BCN-endoK), N-ε-boc lysine (ncAA5, BocK), and a *trans*-cyclo-4-octene lysine equatorial isomer (ncAA6, TCO4)) in the reporter protein's N-terminus (Fig. 5a and b), and we then tested the fluorescence dependence of clickable ncAAs *in vitro* using reported cell-permeable dyes (Fig. 5c–g).

At 1 mM ncAA concentration, PylRS3 incorporated the bulky ncAAs TCO*AK (ncAA2) with higher efficiency compared to BCN-endoK (ncAA4) and BCN-exoK (ncAA3), evaluated as the fluorescence/OD_600_ at *t* = 5 h (1 h after induction) (Fig. 5b). These results are in line with previous *in vitro* experiments,^25^ confirming that PylRS3 shows highly specific aminoacylation efficiency to its native rationally engineered substrate. Interestingly, the ncAA NBK (ncAA1) was incorporated at the highest efficiency, a result not reported previously to our knowledge for any *M. alvus* PylRS system. Overall, these findings suggest that there is a good correlation between the *in vitro* and *in vivo* performance of rationally engineered PylRS systems in GRE6 and that the substrate scope of these engineered systems, as demonstrated by the incorporation of NBK (ncAA1), requires broader characterization.

We used the top three most efficiently incorporated clickable ncAAs: norbornene-lysine (NBK, ncAA1), TCO*A-lysine (TCO*AK, ncAA2), and a bicyclononyne-lysine exo isomer (BCN-exoK, ncAA3), to investigate the behavior of the click product with available dyes containing orthogonal click handles. We tested the commercially available azides and tetrazines compatible with 546 nm and 642 nm excitation wavelengths, used in our optical setup. For the 546 nm excitation series (Fig. 5c), we included the JF549 azide (dye B) and TAMRA *o*-methyl tetrazine (HD555) (dye C). As a non-clickable control in order to compare the fluorogenic propensity, we chose a JFX549 halotag (chloroalkane ligand, HTL) (dye A). For the 642 nm excitation series we chose (Fig. 5f) JF646 HTL (dye D), JF646 azide (dye E), JF646 tetrazine (dye F), rhodamine 101 *N*-methyl tetrazine^49^(dye G), ATTO647N *N*-methyl tetrazine^49^ (dye H), silicon rhodamine o-methyl tetrazine (HD653, dye I),^50^ BODIPY650 azide (dye J), and BODIPY650 tetrazine (dye K). We refrained from using any variants of the Cy3/Cy5 series and sulfonated ATTO dyes, as Kipper *et al.*^15^ demonstrated unspecific retention of these classes of dyes in the cytoplasm of *E. coli*.

At 1 µM dye and 50 µM ncAA, with 30 min incubation time at 37 °C, simulating our *in vivo* reaction conditions, we find very little quenching capacity of the tetrazine-coupled dyes for both the 546 nm and 642 nm excitable series in *in vivo*-mimicking pH 7.5 polymix buffer^51^ (Fig. 5d, e and g, h). As expected, the JF core fluorophores gave the highest fluorescence intensity compared to HD555. HD555 shows the highest turn-on propensity when reacted with both TCO*AK and BCN-exoK as reported; however, this turn-on ratio is significantly lower than that previously reported.^50^ None of the tetrazines showed an increase in fluorescence when reacted with NBK (ncAA1), which is in line with observations that NBK does have the slowest reaction kinetic as a strained alkene,^52^ requiring longer than 30 min for reaction completion. For the 646 nm series, dye G displayed an ∼2× turn-on propensity with TCO*AK, whereas dye H showed an ∼3× increase in fluorescence with both TCO*AK and BCN-exoK, still, significantly lower than that previously reported.^49^ The highest turn-on propensity was noticed between BODIPY650 tetrazine (dye K) and BCN-exoK (3× higher signal), in line with reports for similar structures.^53^ Overall, the results suggest that in a highly reducing intracellular-like environment, turn-on propensities are lower compared to values measured in inorganic solvents and data obtained from mammalian cells, where all of these dyes have been characterized.^48,49,54^ These data further suggest that, for intracellular biophysical studies, JF-based fluorophores should probably be the primary choice for protein labelling, both as HTLs and clickable-ligands.

### SMT validation of FLORENCE

**Fig. 6:**
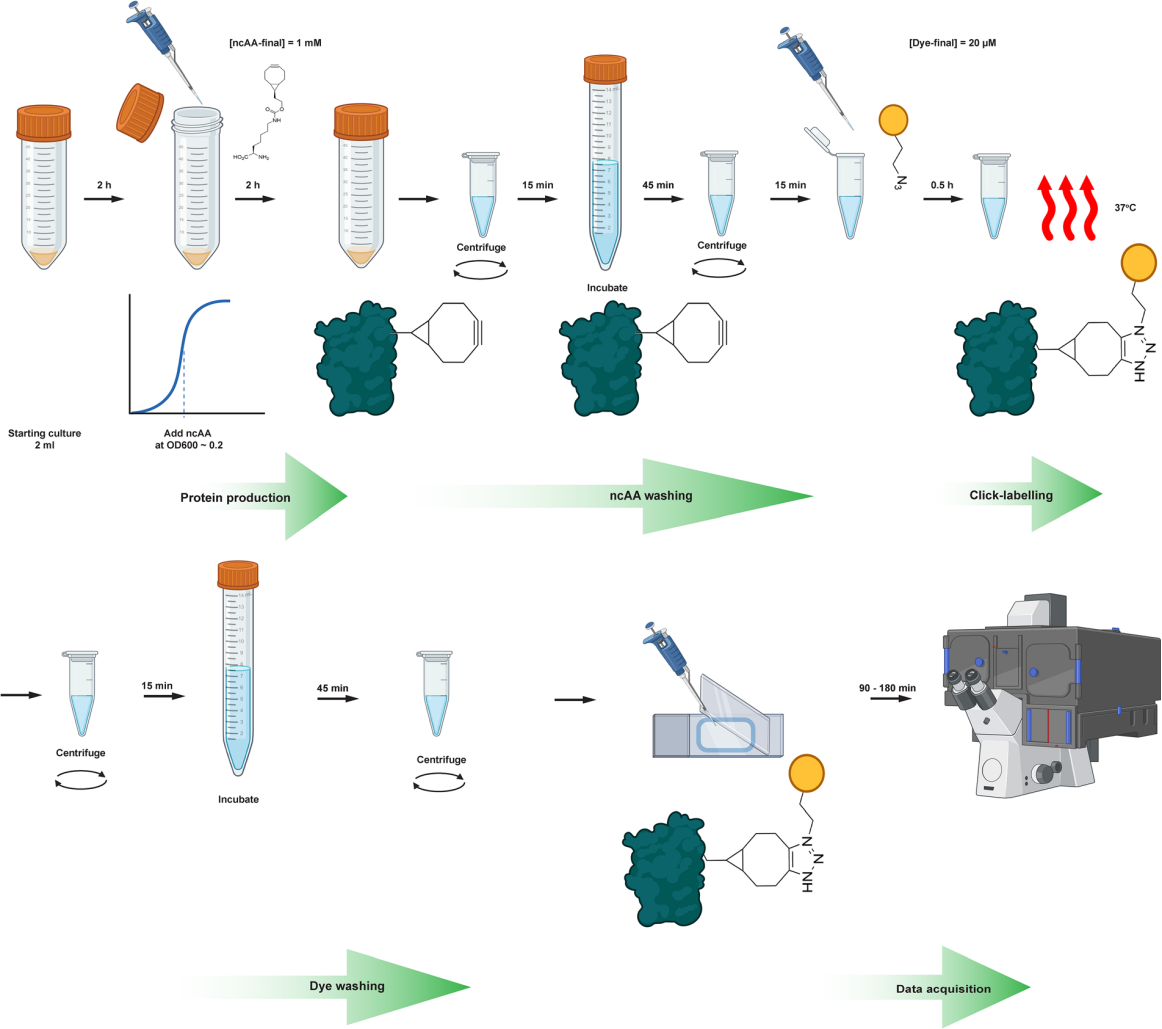
Schematic representation of the ncAA labelling procedure. The protocol starts with inoculation of 10 colonies in 2 mL LB media in a 50 mL Falcon tube supplemented with the antibiotic for plasmid maintenance and growth to OD_600_ ∼ 0.2–0.4. ncAA is supplied at 1 mM final concentration and the protein production is performed for 2 h. After that, 5 consecutive 5 min washing steps are performed (centrifugation at 8000×*g*), with subsequent removal of the supernatant and resuspension of the pellet in 1 mL of M9 1% glucose supplemented with 1.7 µg mL^−1^ pluronics. Next, cells are incubated for 45 min at 37 °C with shaking in 2 mL RDM 0.2% glucose at 37 °C. After repeating the 5 × 5 min washes in 1 mL of M9 1% glucose supplemented with 1.7 µg mL^−1^ pluronics, the azide-dye is added at a final concentration of 20 µM to 100 µL of cellular suspension and the SPAAC is carried out at 37 °C for 30 min in RDM 0.2% glucose. The labelled cellular suspension is then washed, firstly 5 × 5 min washes in 1 mL of M9 1% glucose supplemented with 1.7 µg mL^−1^ pluronics, followed by 45 min incubation at 37 °C in RDM 0.2% glucose, followed by 5 × 5 min washes in 1 mL of M9 1% glucose supplemented with 1.7 µg mL^−1^ pluronics. Two subsequent dilutions of 1 : 10 and 1 : 500 are made of the final cell suspension and 0.5 µL is applied on an agarose pad. The cells are incubated between 60 and 180 min at 37 °C before imaging (depending on the cell growth rate). Compared to Kipper *et al.*,^15^ our experimental procedure decreases the number of washes and omits the step of overnight washes.

In order to benchmark FLORENCE for SMT, we first fused the evolved codon-context linker^26^ to the N-terminus of the HaloTag protein^5^ and expressed it from the pFI4 vector in GRE6. This construct allowed us to label the HaloTag both conventionally, using JFX549-HTL (dye A), and with JF646-azide (dye E) in the presence of the ncAA BCN-exoK (ncAA3) in the same cell culture. As a clickable amino acid, we chose BCN-exoK (ncAA3), despite being the third most efficiently incorporated ncAA. We rationalize the choice of BCN-exoK because of the reported tautomerizations of tetrazines with strained alkenes, which in the course of 30–60 min post-clicking lead to a formation of a non-fluorescence tautomer that requires a further step of oxidation in order to convert back to its fluorescent form.^48^ As a click-reactive fluorophore, we chose JF646-azide because of (i) its outstanding brightness among other clickable partners (Fig. 5g), (ii) demonstrated cell permeability and washability of the JF549-HTL and JF646-HTL dyes used for SMT in *E. coli*,^55^ (iii) higher stability of alkyl-azides compared to tetrazines,^56^ (iv) structural similarity between the HTL and azide linker, with previously demonstrated good permeability and washability,^55^ and (v) decreased cellular autofluorescence and background signal in the >600 nm excitation region.^49^ To investigate any potential problems with unspecific dye retention in the cells, as evident in the work of Kipper *et al.*,^15^ we also included a washing control, using a HaloTag protein construct where the N-terminal linker UAG codon was substituted with a lysine (AAA) codon. A schematic representation of the labeling procedure is shown in Fig. 6.

**Fig. 7:**
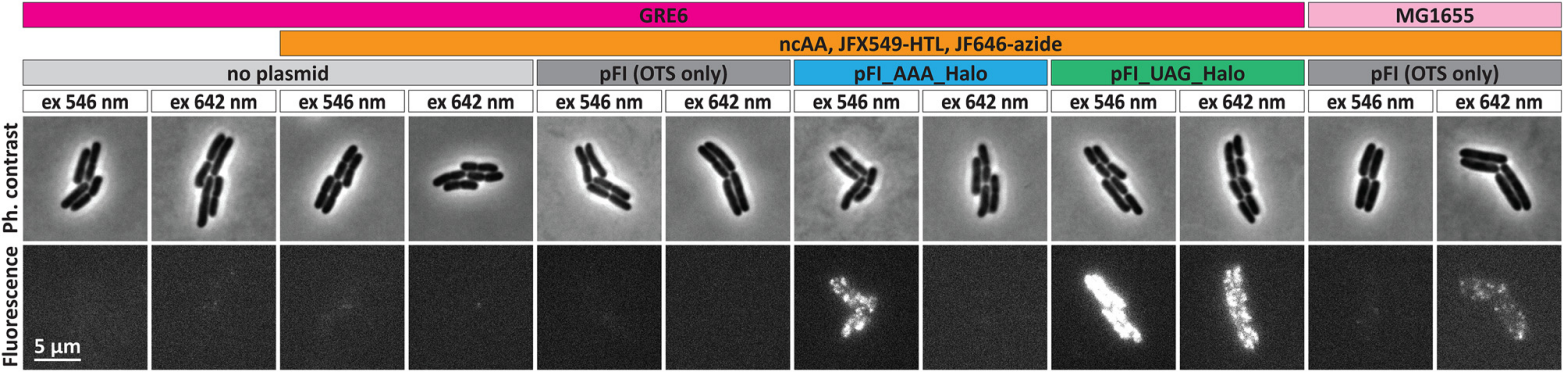
Phase-contrast and fluorescence images of cells expressing HaloTag with a UAG or AAA codon containing context linker fused to its N-terminus. The HaloTag constructs were expressed from the OTS containing pFI4 vector (see Fig. 4a). Empty cells (“no plasmid”) and GRE6 and MG1655 containing the pFI4 vector without the HaloTag gene (“OTS only”) were included as an additional control. Expression of HaloTag was induced by the addition of 1 mM IPTG and 1 mM of the ncAA BCN-exoK (ncAA3). Labelling was performed by adding 20 µM JF646-azide or 1 µM JFX549-HTL at final concentration. Images are representative of *n* colonies, from 2–4 repetitions, where (from left to right), *n* = 62, 63, 89, 77, 94. See the Data availability statement below for access to all raw data.

From the washing controls in GRE6 without any plasmid, we find that the JF646-azide (dye E) does not show any unspecific binding to any cellular component and that the unreacted dye is readily removed during the washing procedure (Fig. 7, “no plasmid”). In the presence of the OTS and the ncAA, without any reporter gene present, retention of JF646 fluorescence species is generally not observed (Fig. 7, “pFI4 (OTS only)”). However, in two of the four experimental replicates made, some of the colonies showed clear JF646 fluorophore retention (see the Data availability statement below for access to all microscopy raw data). Since JF646-azide fluorophore retention is only sporadic, but seemingly homogenous between cells within a colony, we speculate that dye retention in these colonies might be due to an acquired frameshift mutation in a parent cell during the labeling and washing procedure, resulting in a UAG codon expressed somewhere in the transcriptome. Alternatively, this unspecific retention might also be attributed to dye clicking to remaining ncAA-tRNA species, as observed by Arsic *et al.*^57^ in mammalian cells. The half-life of canonical aminoacylated tRNAs has been measured to range between 30 and 800 min.^58^ However, for the PylRS system these numbers remain unknown. While we cannot, with our current data, exclude or confirm either of these scenarios, we speculate that click-labeled ncAA-tRNAs, if present, should occur more homogeneously through all cells, not only in individual colonies.

In the wild-type MG1655 cells, with the OTS and the ncAA, but without any reporter gene present (Fig. 7, “MG1655, pFI4 (OTS only)”), we observed clear presence of JF646 fluorescence in the majority of cell colonies. This suggests off-target incorporation of ncAA, most probably due to the presence of UAG in highly expressed endogenous genes,^21^ thus underscoring the importance of using a recoded organism for site-specific ncAA incorporation.

Retention of JFX549-HTL (dye A) without expression of the reporter HaloTag protein was not observed in any of the above control experiments.

In the presence of expressed HaloTag, we see obvious labeling with both ligand types (JF646-azide and JFX549-HTL) when the UAG codon is present, but only labeling with JF549-HTL when UAG is changed to AAA (Fig. 7). The lack of the JF646 signal when the AAA-containing HaloTag is expressed, again suggesting that the presence of OTS is in general not enough for dye retention (*e.g.*, by dye attachment to ncAA-tRNA) and that dye retention in OTS-only cells occurs sporadically. Hence, overall, these results suggest specific incorporation of the ncAA into the HaloTag protein, with subsequent ncAA-specific click-labelling to JF646-azide.

**Fig. 8:**
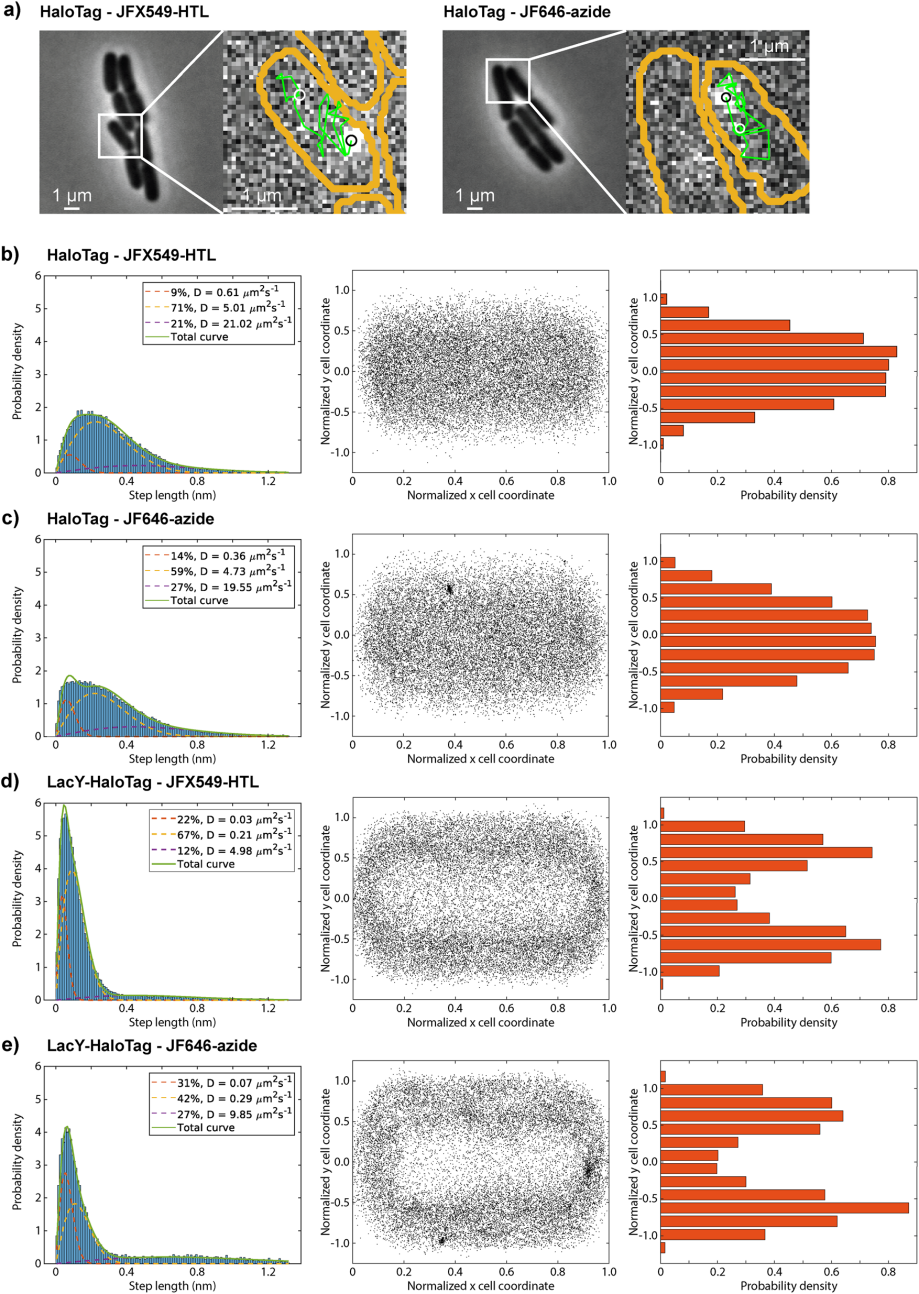
(a) Single-molecule tracking of HaloTag, simultaneously labelled with JFX549-HTL (left, 546 nm excitation), and JF646-azide (right, 642 nm excitation). Cell outlines, segmented from phase contrast images, are shown in orange, and detected diffusion trajectories are shown in green. Current frame localizations are shown as black circles, and the first detected localization as a white circle. (b)–(e) Diffusion and localization of detected fluorophores attached to HaloTag (b) and (c) or LacY-HaloTag (d) and (e) using conventional HaloTag labelling (JFX549-HTL, (b) and (d)) or click-labelling to the UAG codon (JF646-azide, (c) and (e)). Left panels show step-length distributions of tracked particles fitted to three diffusion states. Middle panels show detected particle localizations plotted on normalized cell coordinates (22 207 randomly selected localizations plotted per dataset, with the exception for LacY-HaloTag labelled with JF646-azide (d) where all localizations (22 207) were used). Right panels show the short-cell-axis radial distribution of the particles plotted in the middle panels, where particles in cell poles (0–0.15, 0.85–1) and cell middle (0.35–0.65) were excluded. Data in panels (b)–(e) are representative of *n* = 2–3 independent experiments. Results for all repetitions are shown in Fig. S6. See also Fig. S7 for trajectory steps fitted to 1-state and 2-state distributions, and Fig. S8 for HMM results of all cumulated data. See the Data availability statement below for access to all raw data.

We also performed SMT of HaloTag, simultaneously labelled with both JF646-azide and JFX549-HTL. In the fluorescence movies, we find a high signal-to-background ratio and photostability for the clicked JF646-azide, comparable with those of the JFX549-HTL dye, making it possible to construct single-molecule trajectories (Fig. 8a). From the step-length analysis of the diffusion trajectories (Fig. 8b and c, with individual repetitions in Fig. S6), best fitted to a 3-state model (see Fig. S7 for 1-state and 2-state distribution fittings), we find the diffusion properties of HaloTag to be similar irrespective of the labeling approach, with a main diffusion state at around 5 µm^2^ s^−1^, but with a higher proportion of slowly diffusing particles (∼0.5 µm^2^ s^−1^) for the azide-labelled JF646 dye relative to the HTL-labelled JFX549 dye (14% *vs*. 9%). We hypothesize that these slow-moving particles represent some form of protein aggregates or possible fragments of degraded labelled proteins. Indeed, when cells are allowed to grow and divide for longer periods of time, we find sporadic immobile dots, suggestive of increased protein degradation or aggregation with time. In line with previous SMT of electroporated tRNAs,^7^ SMT analysis and the washing controls were, hence, performed only on cell colonies with approximately 2–8 cells. By analyzing the diffusion trajectories using a more sophisticated Hidden-Markov Model (HMM) approach,^8,59^ we also find very good agreement between the two different labeling approaches (Fig. S8 and Supplementary Dataset 1), again with a slightly higher fraction of slowly diffusing particles for JF646-azide relative to JFX549-HTL (8% *vs*. 4%)).

Finally, as a separate control, we fused the UAG-containing codon-context-linker-HaloTag construct C-terminally to the inner membrane protein lactose permease (LacY) and repeated the dual labeling experiment. Again, based on both step-length analysis (Fig. 8d-e) and HMM analysis (Fig. S8 and Supplementary Dataset 1), we get similar diffusion parameters irrespective of the label tracked, now with a main diffusion state at around 0.2–0.3 µm^2^ s^−1^. The spatial distributions of the detected fluorophores attached to LacY-HaloTag, also show clear membrane localization for both dyes (Fig. 8d and e), which is not the case for HaloTag only (Fig. 8b and c), providing further evidence of site-specific click-labeling of a protein of our choice inside living *E. coli* cells.

## Discussion

We present an improved method for installing site-specific minimal fluorescent labels into proteins in re-coded *E. coli* cells, with high specificity compared to previous studies, to allow for user-friendly, live-cell single-molecule tracking studies. GCE is a complex technology that encompasses three fundamental parts: an orthogonal translation system, a model host, and the chemical nature of the ncAA and dyes. These parts have primarily been engineered as separate entities in order to increase protein production yield. While protein yield is the major focus in the context of therapeutics production, the requirements for the use of GCE for biological studies using SMT adds additional criteria. Firstly, biological studies require minimal disturbance to the system of study, *i.e.*, a living, dividing cell. Secondly, as clearly demonstrated by Kipper *et al.*,^15^ for SMT, incorporation of the ncAA and subsequent reaction to the clickable dye must be very specific and fast, and, perhaps most important, the labelling chemical entities (ncAA and dye) need to be washable. In the current study, we report a complete system for *in vivo* click-labeling and SMT of proteins, where these criteria have been fulfilled.

Based on the fact that aminoacylation of tRNA^Pyl^ is the first critical step for any ncAA incorporation, we investigated which of the currently available PylRS systems can be applied for insertion of clickable amino acids. Since we initially observed a rather low reporter signal for our three tested candidates (PylRS1–PylRS3, Fig. 2c), we first optimized the vector system. We hypothesized that low protein expression levels might be due to heterogeneity in the plasmid copy number, either for the reporter or for OTS plasmid. This is not uncommon for the reported plasmid used, pQE30lacIq.^60^ However, based on the clear signal in the positive control, this suggests that the PylRS expression is the limiting part. We therefore constructed two plasmid variants where the reporter protein gene was inserted on the OTS plasmid (Fig. 2d and Fig. S1a) and saw a marked difference in our protein expression level (Fig. 2e–g, and Fig. S1b–d). There was, however, a clear difference in reporter protein production, depending on which plasmid design was used. Our results, thus far, show that there is a large variability in gene expression depending on plasmid design, and that, at least for our system, expressing the reporter protein from the same plasmid as the OTS is beneficial.

Further, although PylRS3 does not significantly differ in ncAA incorporation *in vivo* as compared to the other tested PylRS (Fig. 2e–g), its reported higher solubility could potentially be beneficial in cases of overexpression of challenging proteins, decreasing the possibility of aggregate formation. We demonstrate that indeed, this more recently reported PylRS3 system is actually fully functional *in vivo* and performs well compared to the commonly used PylRS1.

In order to perform ncAA click-labelling, without risking labeling of additional proteins due to ncAA incorporation on endogenous UAG stop codons, a UAG-free strain is the desired host system. However, as our goal is to study the *in vivo* kinetics of proteins involved in physiological processes, this UAG-free strain should, preferentially, be as close in physiology and genotype as possible to the current lab model strains. The C321 lineage^20^ was constructed from an MG1655 strain, which is the most common model in microbiology and is a suitable candidate for this purpose. We, indeed, observed defects in growth and cellular morphology of the C321 progeny, GRE1 (C321.ΔA.M9.adapted) (Fig. 3b), and we addressed these by reverting some of the mutations in this strain as per literature findings. We observed significant improvement in cell morphology at 37 °C in LB after removing the lambda red prophage cassette and restoring the *mutS* gene, in order to decrease the rate of spontaneous mutations and obtain genome stability (Fig. S3a and b). This is particularly important for the application in physiological studies, where biological processes are optimized to operate best at 37 °C. Although GRE6 grew in RDM at 37 °C, we, however, did not find any significant improvement in its fitness compared to GRE2, *i.e.*, when reverting the mutations reported by Hemez *et al.*^32^ and Tyer *et al.*^36^ (Fig. S2, S4 and S5). Interestingly, the fact that the removal of the frameshift mutation in *ilvG* to B genotype, abolishing isoleucine starvation, did not result in significant decrease of the doubling time in RDM, suggests that there is a strong re-wiring of the metabolome for GRE1–GRE6, which requires further specialized studies.

When the single-plasmid construct that gave the highest reporter signal in MG1655 was moved to our optimized GRE6 strain, we were surprised to see no UAG-containing reporter signal upon induction (Fig. 4c). Since the positive control reporter, with UAG changed to AAA, still gave a very high signal (Fig. 4b), the problem had to be related to the OTS expression. The metabolome re-wiring in the C321 lineage has been reported to result in a change of carbohydrate catabolic pathways,^38^ such as that of arabinose. To investigate whether the lack of reporter expression was related to the arabinose dependent induction, also in line with recent findings from the Ellington lab,^42^ we constructed a number of additional plasmids where the expression of PylRS3 was either induced by anhydrotetracycline, IPTG, or constitutively expressed. Interestingly, no reporter signal was found from any of the inducible expression systems, suggesting that there are some regulatory elements in the current background that limit the usage of these systems for PylRS3 expression. The constitutive expression of PylRS3, however, resulted in clear reporter production (Fig. 4). Our observations, thus, complement previous works, suggesting that constitutive over inducible expression of PylRS shows improved ncAA-based protein production.^61^ Moreover, comparing the reporter protein expression from a construct containing a codon-context linker (Fig. 4f), with the UAG codon inserted at position 26 (Fig. 4i) or 45 (Fig. 4j) within the reporter gene, demonstrates that there is lower but still significant ncAA incorporation independent of the codon-context linker. These results demonstrate that the present system has applicability for site-specific labelling beyond the presence of a context linker.

Since the properties of the final click-product are influenced by the side-chain of the click-amino acid,^48^ we also wanted to test partners with different reactivities. Except for TCO4K (ncAA6), we find reporter protein production to varying extent with all ncAAs tested (Fig. 5), showing that PylRS3 has a broad substrate scope of commercially available clickable ncAAs, offering increased opportunities for encoding multiple clickable ncAAs with orthogonal handles. Our findings confirm that PylRS engineering is indeed one of the key segments of GCE for the development of rapid and efficient protein production. Moreover, we find that PylRS3 can discriminate between different diastereisomers of the ncAA's side chain, as reflected in the striking differences between reporter production in the presence of BCN-exoK (ncAA3) and BCN-endoK (ncAA4) (Fig. 5).

Before turning to microscopy, we also partly surveyed the brightest available and cell-permeable fluorescent dyes that would be compatible with the top three ncAAs incorporated most efficiently in our reporter assay. The tested tetrazine dyes (Fig. 5c and f) have been reported to demonstrate strong turn-on ratios in various buffers and inorganic solvents,^48,54^ potentially providing an advantage in our system. Surprisingly, however, after the 30 min click-reaction incubation in the phosphate based polymix buffer used in our experiments, very weak, if any, increase in fluorescence was observed (Fig. 5). As polymix includes DTT, thereby mimicking the highly reducing cytoplasm of *E. coli*, these observations might reflect actual differences in the chemical properties of the dyes depending on the buffer conditions used. Based on the very high fluorescence of the Janelia Fluor series of dyes (Fig. 5d and g), and our previous positive experience with these dyes for live-cell SMT,^2,27,33^ we decided to pursue SMT with those.

With all components in place, we benchmarked the system using dual labeling of the commonly used HaloTag protein, that is, with a chloroalkane ligand (HTL) for conventional HaloTag labeling, and a UAG codon for ncAA click-labeling using an azide dye, we confirmed specific *in vivo* protein labeling (Fig. 7). Compared to the work of Kipper *et al.*,^15^ with our optimized protocol (Fig. 6) for labeling and washing, and the use of the cell-permeable and bright JF dyes, we overcome the problem of unspecific retention of dyes inside the cells. It should also be noted that the present experimental protocol is significantly faster (10–12 h) compared to that reported by Kipper *et al.*^15^ (48 h). With the OTS present, however, we found in two out of four experiments colonies with apparent click-labeling of a protein, despite the lack of a UAG containing reporter protein. This was not observed in the experiments with an AAA codon reporter protein, where we would expect the absence of a click signal. Hence, we speculate that the sporadic dye retention observed in the “OTS only” experiments might be due to a frameshift mutation during the labeling and washing procedure, resulting in a UAG codon being present somewhere in the native transcriptome. Alternatively, this signal can result from remaining tRNA-ncAA complexes, as has been shown to occur in mammalian cells.^57^ Hence, for future use of the click-labeling approach for live-cell imaging, it will be important to keep such potential, although rare, unspecific labeling in mind. Application of the PylRS3 system in a UAG-containing wildtype strain (MG1655) resulted in more frequent presence of bright dots, suggesting genome-wide incorporation. This is indeed in line with observations by Mihaila *et al.*^62^ when using GCE labelling in a BL21-DE3 strain with a newly engineered OTS. Finally, we validated the experimental system by performing SMT of HaloTag and LacY-HaloTag, both dually labeled using HTL or azide dyes. Although minor differences exist in diffusion properties depending on label tracked (Fig. 8), these SMT experiments provide solid evidence of protein specific *in vivo* click-labeling. Furthermore, to our knowledge, we provide the first report that the strain-promoted azide–alkyne click-chemistry (SPAAC)^63^ reaction occurs as fast as 30 min in *E. coli* cells and allows tracking of intracellularly labelled proteins.

In summary, we report an improved method for *in vivo* protein labelling with fluorescent dyes bright and photostable enough for SMT. Compared to the HaloTag and SNAP-tag, which are typically fused N- or C-terminal to the POI, our improved system, FLORENCE, will allow site-specific protein labelling. We hope that the SMT community will adopt and develop this method further in the quest to answer some of the most challenging remaining biological questions that require real-time analysis of protein binding kinetics inside living cells.

## Methods

### Strain construction

Chromosomal gene replacements and insertions were done by lambda red recombineering, following the protocol from Knöppel *et al*.^64^ The selectable/counterselecatble markers used are *Acatsac1* (GenBank accession ID: MF124798.1). For chromosomal deletions and single-point mutations at non-essential genes, the protocol according to Näsvall^65^ was used. Oligonucleotides used for this part are reported in Table S3. The only modification of the reported protocol is the amplification of the *Acatsac1* templates using Q5 High-fidelity polymerase (New England Biolabs), with 1000 bp min^−1^ extension time. MAGE was performed for introducing single-point mutations in essential and multiple genes according to the protocol of Nygeres *et al*.^66^ The only deviation from that protocol is the temperature curable pSIM5-ORTMAGE2 plasmid constructed in this work. The genotypes of the constructed strains are described in Table S2. The oligonucleotides used for the CatSacB construction are found in Table S3. The oligonucleotides for MGE are reported in Table S4.

C321.ΔA.M9adapted (Addgene plasmid # 98568) was a gift from George Church. The pEVOL plasmid encoding the *Methanosarcina mazei* tRNA^Pyl^_CUA_/PylRS^AF^ pair was a gift from Johan Elf. The pET28 vector with genes encoding the *Methanomethylophilus alvus* PylRS^ALIP^ (PylRS3) and PylRS^AAIP^(PylRS2), and pUC19 tRNA^Pyl^ were a gift from Shigeyuki Yokoyama. pMEGA-MaPylRS (Addgene plasmid #200225) was a gift from Alanna Schepartz. pORTMAGE-2 was a gift from Csaba Pal (Addgene plasmid #72677) and was provided by Joakim Näsvall.

### Plasmid construction

For gene amplification of constructs, polymerase chain (PCR) reaction was performed using Q5 High-fidelity polymerase (New England Biolabs) according to the manufacturer's protocol. T4 ligase (NEB) was used to ligate linearized phosphorylated PCR products according to the manufacturer’ protocol. Table S1 contains all plasmids used in this study. Table S5 contains all primers used for the construction of the plasmids in this study. Table S6 describes the construction of the plasmids in this study.

### Chemicals

HD555 and HD653 were kindly provided by Richard Wombacher from Max Planck Institute for Medical Research Heidelberg. BCN-exoK, BCN-endoK, TCO*AK and and TCO-4K were purchased from Sirius fine chemicals (SiChem GmbH) Bremen, Germany. Norbornene-K was purchased from Iris Biotech Gmbh. JF646-azide and JF646 tetrazine were purchased from Tocris Bioscience. BODIPY650 azide and BODIPY630 tetrazine were provided by Lumiprobe. JFX549 Halo and JF646 Halo were a gift from Luke Lavis at Janelia Farm HHMI. Dyes Rhodamine 101 *N*-methyl tetrazine and ATTO657 *N*-methyl tetrazine were kindly provided by Peng Wu, Beijing University. All amino acids were dissolved in 0.2 M KOH to an initial concentration of 100 mM and aliquoted in 1 mL Eppendorf tubes and stored in single-use aliquots at −80 °C. Seaplaque GTG Agarose Lonza was used to prepare agarose pads at 2% weight/volume final concentration. RDM 0.2% glucose was purchased from TekNova. 10× polymix buffer was provided by Suparna Sanyal's lab. The Pluronic F-127 surfactant was purchased from Merck.

### Measurement of doubling time

A sample of glycerol stock stored at −80 °C was stroke on an LB plate and incubated at 37 °C, with the exception of GRE1, which was incubated at 30 °C overnight. The next day, a single colony was inoculated in 1 mL of LB or 1 mL of RDM (supplemented with a final concentration of 1 µg mL^−1^ of biotin for GRE1 only) and grown to OD_600_ ∼ 0.4. Only the cultures of GRE1 were grown at 30 °C. Cultures were diluted 1 : 500 in the same type of media and 150 µL were transferred to a Costar 96 well plate at a Biotek Synergy H1 plate reader with continuous double orbital shaking at 37 °C. OD_600_ was monitored every 5 min. Doubling time was calculated by fitting an exponential function of the first ten data points starting from OD_600_ ≥ 0.015 after blank subtraction.

### Fluorescence reporter plate reader experiments

All experiments were performed on a Synergy H1 microplate reader with the accompanying Gen5 software, with a double orbital shaking of 423 cpm, a reading height of 6.75 mm and gain set to 90. The fluorescence read was performed on top of a 96 dark side Costar plate. For data shown in Fig. 2c, 25 µg mL^−1^ kanamycin together with 12.5 µg mL^−1^ chloramphenicol was supplied. For the data in Fig. 2e–g and Fig. S1, cells were cultured with 12.5 µg mL^−1^ chloramphenicol. For the data in Fig. 4, cells were cultured with 34 µg mL^−1^ chloramphenicol from a single colony until OD_600_ ∼ 0.4 and were diluted in a 1 : 500 ratio in 1 mL of LB media. The sample from a −80 °C glycerol stock was stroke on either a 12.5 or a 24 µg mL^−1^ chloramphenicol plate (with the exception for Fig. 2c where a double antibiotic plate with the respective concentrations was used) and incubated overnight at 37 °C in LB media. A single colony was inoculated in a 10 mL polypropylene culture tube containing chloramphenicol at a final concentration of either 12.5 or 34 µg mL^−1^. Cultures were grown to OD_600_ ∼0.4 and were diluted to the same optical density in a 1 : 500 ratio in 6 mL containing media with the appropriate antibiotic and were grown until OD_600_ ∼ 0.2–0.4 after which, arabinose at a final concentration of 0.02%, IPTG 1 mM and ncAA 1 mM were added by using a multichannel pipette and left grown overnight. For the pASK plasmid, anhydrotetracycline was added at a final concentration of 0.2%.

### Determination of turn-on ratios

All measurements were performed at 37 °C in a black Co-star 96 well plate on a CLARIOstar Plus (BMG LABTECH, Ortenberg, Germany). Turn-on ratios were measured by preparation of final 1 µM solution of the designated fluorophore in polymix buffer. Fluorescence was measured at 546 nm excitation and 571 nm emission wavelengths for dyes A–C, at a focal height of 6.8 mm and gain set to 1119. Fluorescence was measured at 642 nm excitation and 680 nm emission wavelengths for dyes D–K at a focal height of 7 mm and gain set to 2202. A final concentration of 50 µM of the designated ncAA was added to each well prepared in MilliQ water and the fluorescence was measured at the appropriate wavelengths after 30 min of incubation at 37 °C.

### Cell phenotype evaluation and single-cell analysis

A microfluidic mother machine device was fabricated following the standard protocol according to Baltekin *et al*.^37^ Briefly, a single colony of GRE1 and GRE6 from an LB plate was inoculated in 3 mL of LB supplemented with 85 µg mL^−1^ final concentration of Pluronic and incubated at 37 °C for 2h. After OD_600_ ∼ 0.4, the culture was diluted and loaded on a previously prepared mother machine microfluidic device. 5 positions were randomly selected and imaged every 5 min in the course of 5 h. The same protocol was followed for determination of the growth rates of GRE2 and GRE6 in both RDM and LB with the exception that the imaging of the strains started immediately after loading and imaged every minute in the course of 2–4 h for RDM and 4 h for LB. The single-cell analysis was performed by using the models and scripts reported in Brandis *et al.*^67^

### Preparation of cells for microscopy

A glycerol stock of GRE6 with the appropriate construct was plated on an LA + 34 µg mL^−1^ chloramphenicol plate and incubated at 37 °C overnight. The next day, 10 colonies were inoculated in 2 mL of LB media supplied with 34 µg mL^−1^ final concentration of chloramphenicol in a 50 mL red-cap falcon tube. The culture was incubated at 37 °C at 200 rpm shaking for 2 h, until OD_600_ ∼ 0.4. For the HaloTag construct, BCN-exoK and IPTG both at final concentration of 1 mM were added into the tube and the cells were left to incubate for 2 additional hours. In the case of LacY, IPTG induction was omitted. The cell culture was separated in 2 1.5 mL Eppendorf tubes and spun on a benchtop centrifuge for 5 min at 8000×*g*. After discarding the supernatant, the pellet was washed with M9 1% glucose minimal media with 1.7 µg mL^−1^ final concentration of Pluronic. The cells were washed 5 times at 8000×*g*, by re-suspending the pellet in a new tube after each wash. Cells were then incubated in RDM 0.2% glucose media for 45 min at 37 °C in a 200 rpm incubator and the identical washing procedure was repeated. The pellet was re-suspended in 50 µL of RDM 0.2% glucose media and JF646-azide and the JFX549-HTL ligand were added at 20 µM and 1 µM final concentrations respectively. In a separate tube, the same culture was labelled with 1 µM final concentration of JF646-HTL. The cell suspension was incubated at 37 °C for 30 min, after which the same washing steps were applied. Cells were re-suspended in 100 µL RDM, after which 1 : 10 and 1 : 500 serial dilution was performed. 0.5 µL of cell dilution was applied on a previously prepared 2% weight percentage agarose pads surrounded by a double-sided sticky Gene Frame (ThermoFisher Scientific) on a glass microscope slide and covered with a precision cover slip #1.5H (Thorlabs). Cells were imaged after 90–180 min incubation on the microscope at 37 °C.

### Optical setup

Widefield epifluorescence microscopy was operated on an inverted Nikon Ti2-E microscope equipped with a CFI Plan Apo Lambda 100×/1.45 objective. The system was enclosed in an OKOlab incubation chamber (model H201-ENCLOSURE) with a temperature control unit (H201-TUNIT-BL), maintaining the sample experimental temperature at 37 ± 2 °C. Images, including phase contrast, bright-field, and fluorescence time-laps, were captured using an Orca Quest camera (Hamamatsu) mounted to the microscope *via* an additional demagnifying 0.7 ×  camera adapter. The JFX549-HTL label was excited using a 546 nm laser (2RU-VFL-P-2000-546-B1R, 2000 mW, MPB Communications) at a power density of 3 kW cm^−2^ at the sample plane, operated in stroboscopic mode with 3 ms laser pulses during each 5 ms camera exposure (20 ms for the LacY construct). The JF646-azide label was excited using a 642 nm laser (2RU-VFL-P-2000-642-B1R, 2000 mW, MPB Communications) at a power density of 6.7 kW cm^−2^, also in stroboscopic mode with 3 ms pulses per 5 ms exposure (20 ms for the LacY construct).

For SMT, each mini-colony was imaged in 800 to 2000 frames to generate time-lapse fluorescence movies. The microscope was operated *via* the μManager software, and multi-position imaging was achieved using custom-developed μManager plugins.

### Single-molecule tracking data analysis

SMT data analysis was performed with previously published MATLAB-based analysis pipelines. First, cell segmentation was performed using an algorithm which finds the boundaries of ellipse-shaped objects.^68^ The segmentation was manually curated to exclude any colonies with approximately more than 8 cells. Cells with an average fluorescence of 1.1× or lower than the average background for the first 10 frames were also excluded to reduce the detection of background noise. Fluorescent dots were detected using a radial symmetry method.^69^ The detected dots were then refined and the localization errors were taken into account by symmetric Gaussian modeling and maximum *a posteriori* fitting.^59^ Trajectory building was conducted with the uTrack algorithm,^70^ when maximum 3 dots per cell remained in the cell. Gap filling, *i.e.*, missing dots, for 2 consecutive frames was allowed to account for blinking effects. Trajectories shorter than 5 frames were excluded from the analysis. Dots were excluded if the amplitude was lower than 250 photons to reduce detection of noise. Dots more than 3 pixels outside of the cell boundaries were also not included in the analysis. Lastly a search radius of 20 pixels (1314 nm) was used to connect dots into diffusion trajectories. The trajectories were then analyzed to find the diffusional state coefficients by fitting the histogram of the experimental step lengths to the theoretical step length probability distribution function, by minimizing the absolute error in each bin. The trajectories were further analyzed using a Hidden Markov Model (HMM) approach, previously described,^3,8,27^ and all trajectories were fitted to 1 to 3 state models.

## Author contributions

M. J. and I. L. V. conceived the project. M. J. supervised the project with input from I. L. V. and G. B. F. I. and G. B. designed procedures for generation of plasmid constructs as well as generation of the GRE variants. F. I. performed all preparative and experimental work. L. W. assisted in optimization of the click-labeling protocol and analyzed single-molecule tracking data. A. B. and I. L. V. assisted in data analysis. F. I. and M. J. wrote the manuscript with input from all other authors.

## Figure licenses

Parts of figures were created using BioRender. Licenses will be provided upon final submission.

## Unique biological materials availability

All unique biological materials are available from the corresponding author upon request.

## Conflicts of interest

The authors declare no competing interests.

## Supplementary Material

CB-OLF-D5CB00221D-s001

CB-OLF-D5CB00221D-s002

CB-OLF-D5CB00221D-s003

## Data Availability

The data that support this study are available in the SciLifeLab Data Repository: https://doi.org/10.17044/scilifelab.29939984. Supplementary information (SI) is available. See DOI: https://doi.org/10.1039/d5cb00221d.
