# Supplementary material for: Optimization of the genetic code expansion technology for intracellular labelling and single-molecule tracking of proteins in genomically re-coded E. coli
Source: RSC Chem Biol. 2025 Nov 24;7(2):269–85. doi: 10.1039/d5cb00221d (PMC12684252; doi:10.1039/d5cb00221d)
Supplement: CB-007-D5CB00221D-s002 [file CB-007-D5CB00221D-s002.pdf]

Supplementary Information (SI) for RSC Chemical Biology.
This journal is © The Royal Society of Chemistry 2025

Supplementary Information to:

Optimization of the genetic code expansion technology for intracellular labelling and single-molecule tracking of proteins in genomically re-coded *E. coli*

Filip Ilievski, Linnea Wikström, Anneli Borg, Ivan L. Volkov, Gerrit Brandis, *Magnus Johansson

Department of Cell and Molecular Biology, Uppsala University

*Correspondence: m.johansson@icm.uu.se

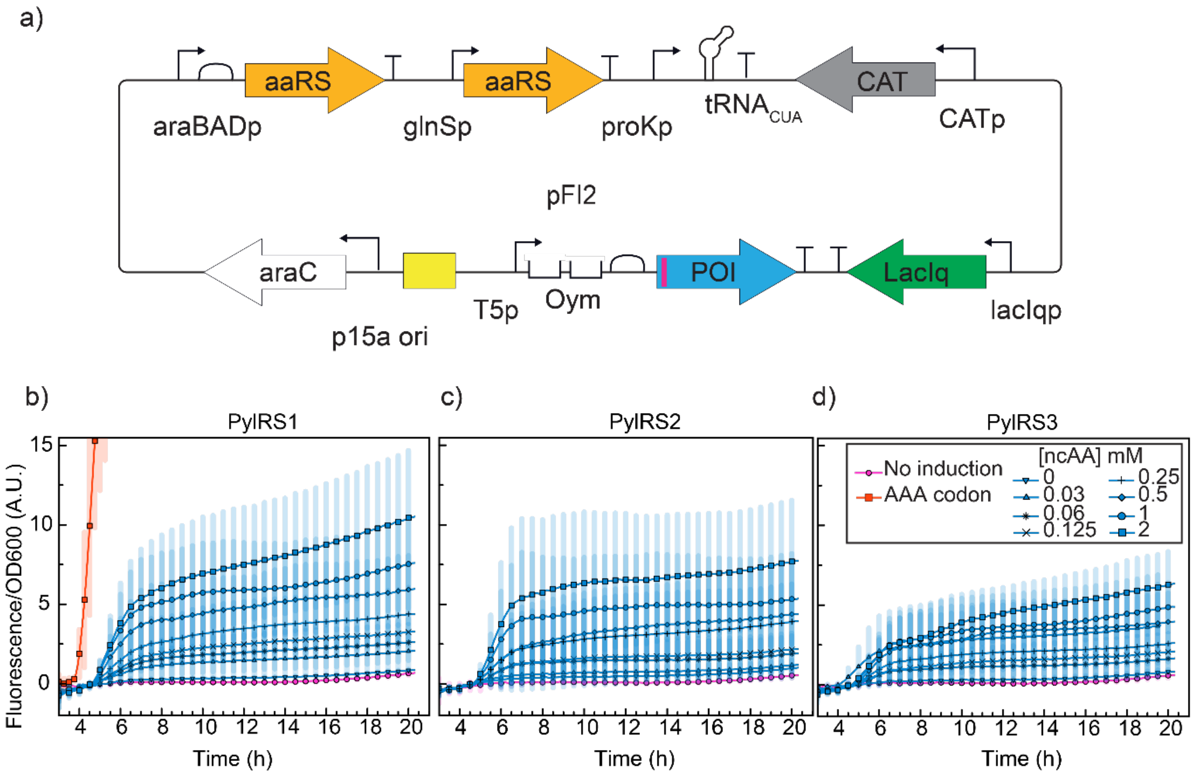

Supplementary Fig. 1. **a)** Design of a **pFI2** variant for ncAA incorporation, where the position of the reporter gene cassette is different. **b-d)** Reporter fluorescence signal normalized to cell density (OD_{600}) for the three PylRS tested on the variant plasmid. The experiments were performed in presence of increasing concentrations of TCO*AK, with co-induction of the OTS with 0.02% arabinose, and 1 mM IPTG of the reporter. A UAG-to-AAA mutation in a separate, identical construct is used as a positive control. Data is representative of three independent replicates.

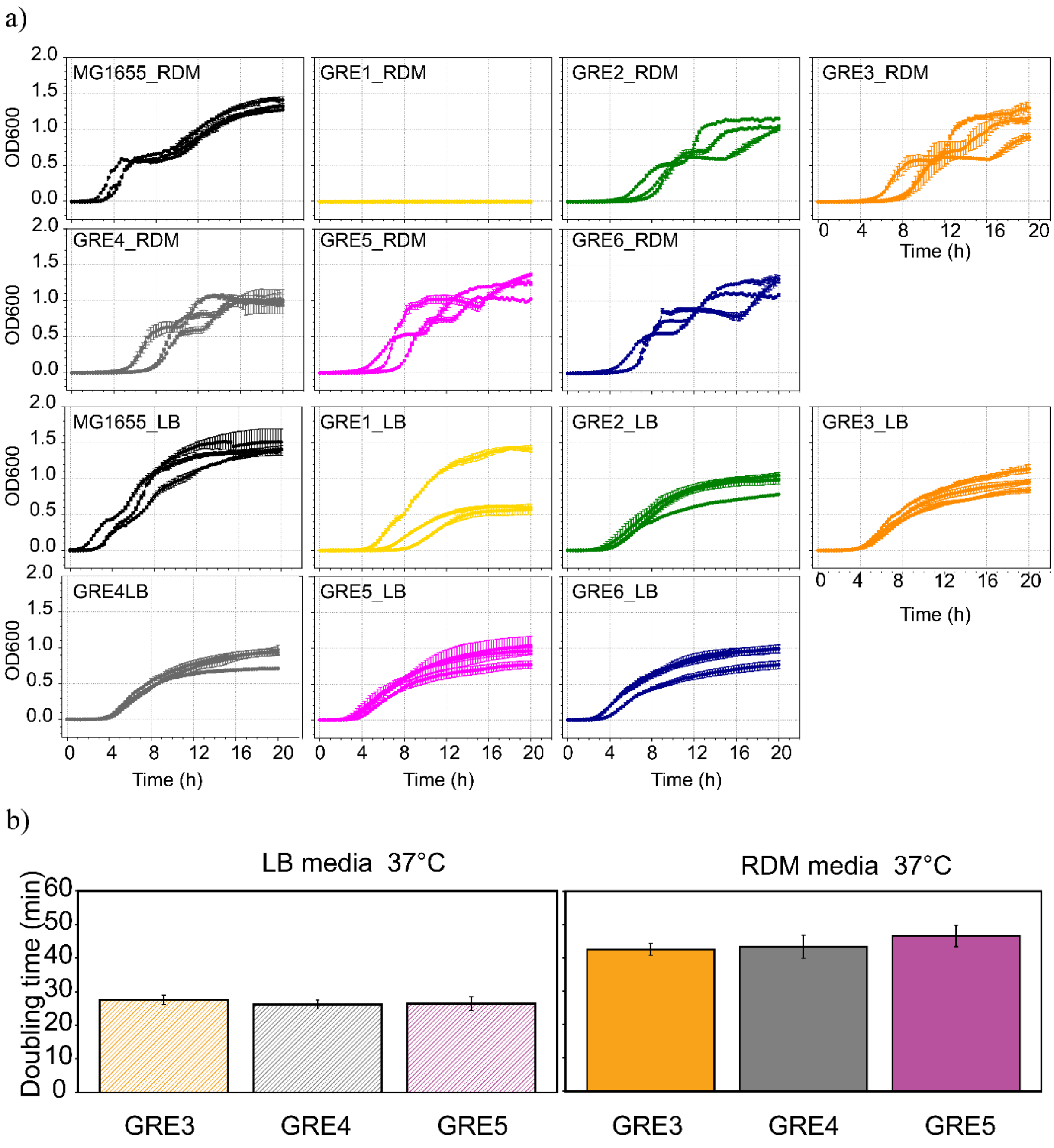

Supplementary Fig. 2. a) Growth curves of individual biological replicates for main **Fig. 3c** in RDM and LB media at 37ºC. Single colonies were inoculated in 1 mL LB or RDM media, grown to OD600 ~ 0.4, diluted 1:500 and grown for 20 h. b) Doubling times calculated for **GRE3-GRE5** based on the growth curves in panel a. Data is representative of n = 3 biological replicates.

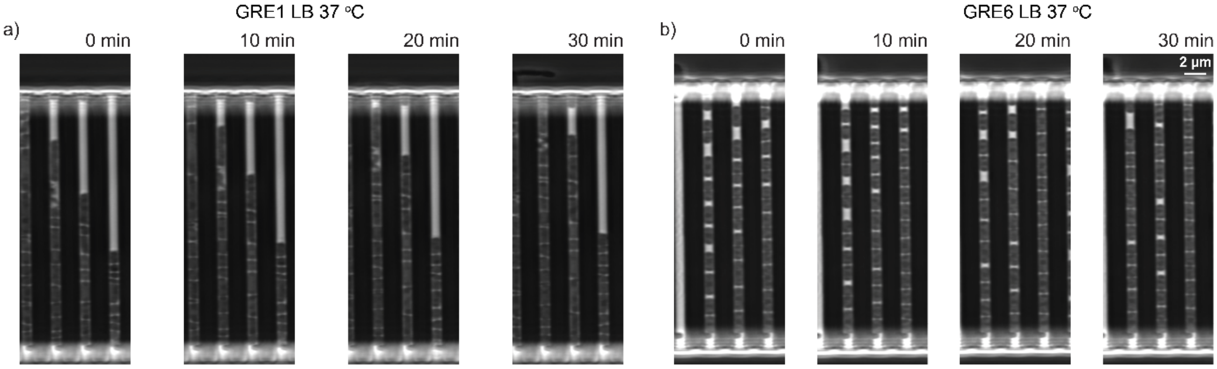

Supplementary Fig. 3. Phase-contrast images of **GRE1** (**a**) and **GRE6** (**b**) grown in mother-machine microfluidic cell traps. Cells were loaded and incubated for 60 min before imaging. Images are representative of n = 3 biological replicates.

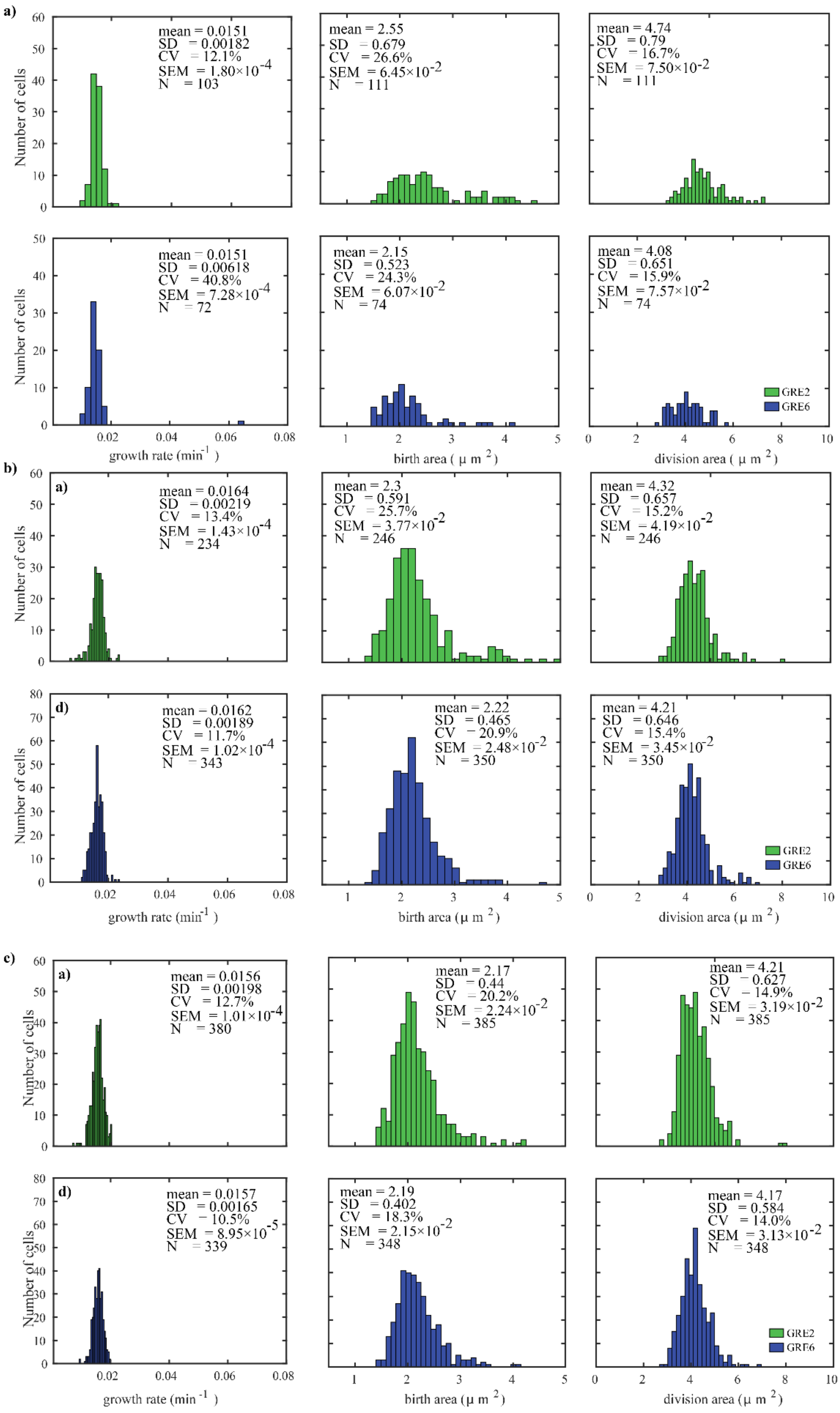

Supplementary Fig. 4. Determination of growth rate, area at birth, and division area for **GRE2** and **GRE6** in RDM at 37 °C on a mother-machine microfluidic device. Imaging was performed every minute in the course between 2h and 4h. The results show three individual replicates from 5 positions per replicate per strain. Individual statistics are shown in the respective panels. The model for segmentation and analysis script from calculation is adapted from *Brandis et al.*[1]

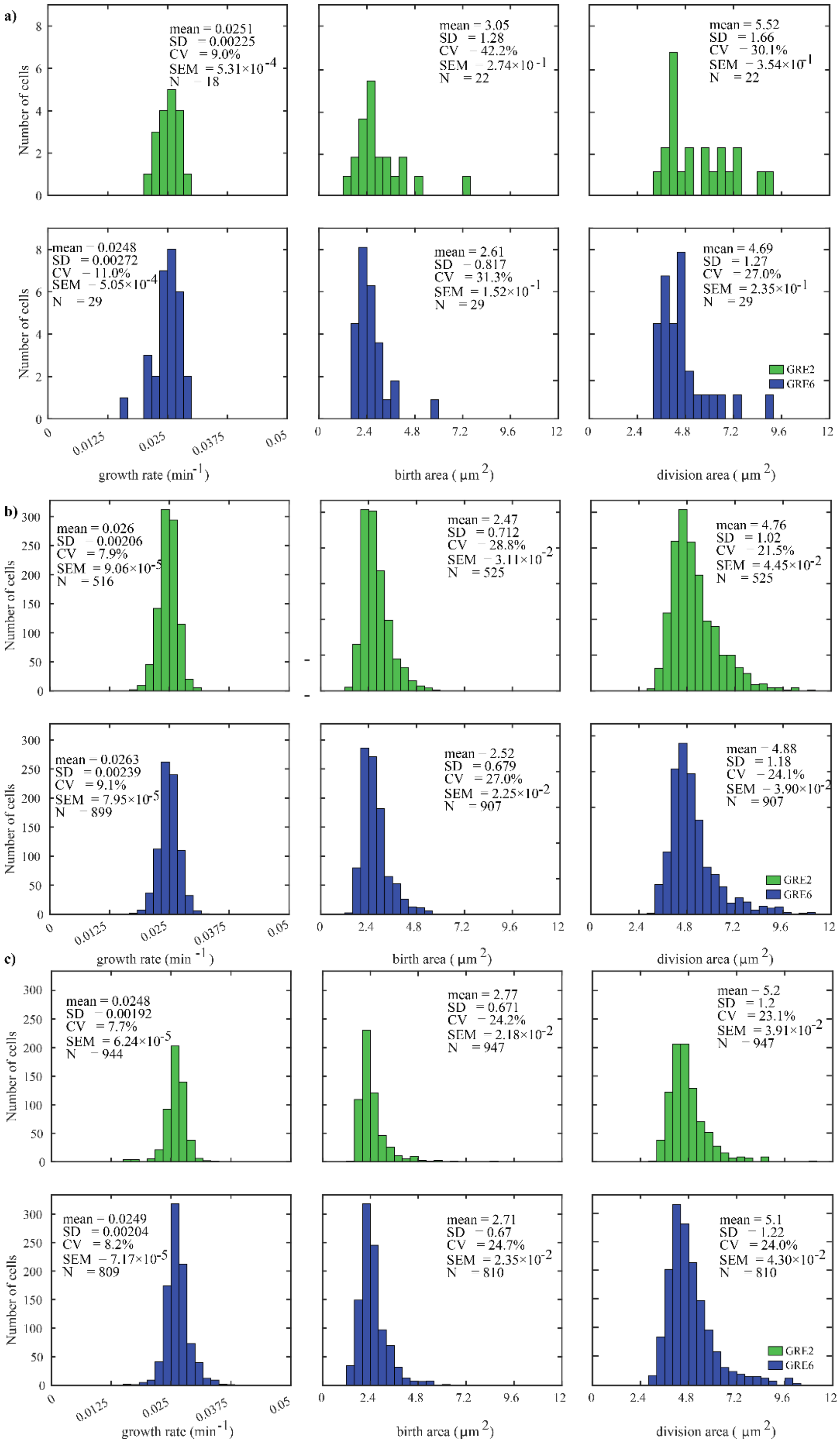

Supplementary Fig. 5. Determination of growth rate, area at birth, and division area for **GRE2** and **GRE6** in LB media at 37 °C on a mother machine microfluidic device. Imaging was performed every minute in the course of 4h. The results show three individual replicates from 5 positions per replicate per strain. Individual statistics are shown in the respective panel. The model for segmentation and analysis script from calculation is adapted from *Brandis et al.*[1]

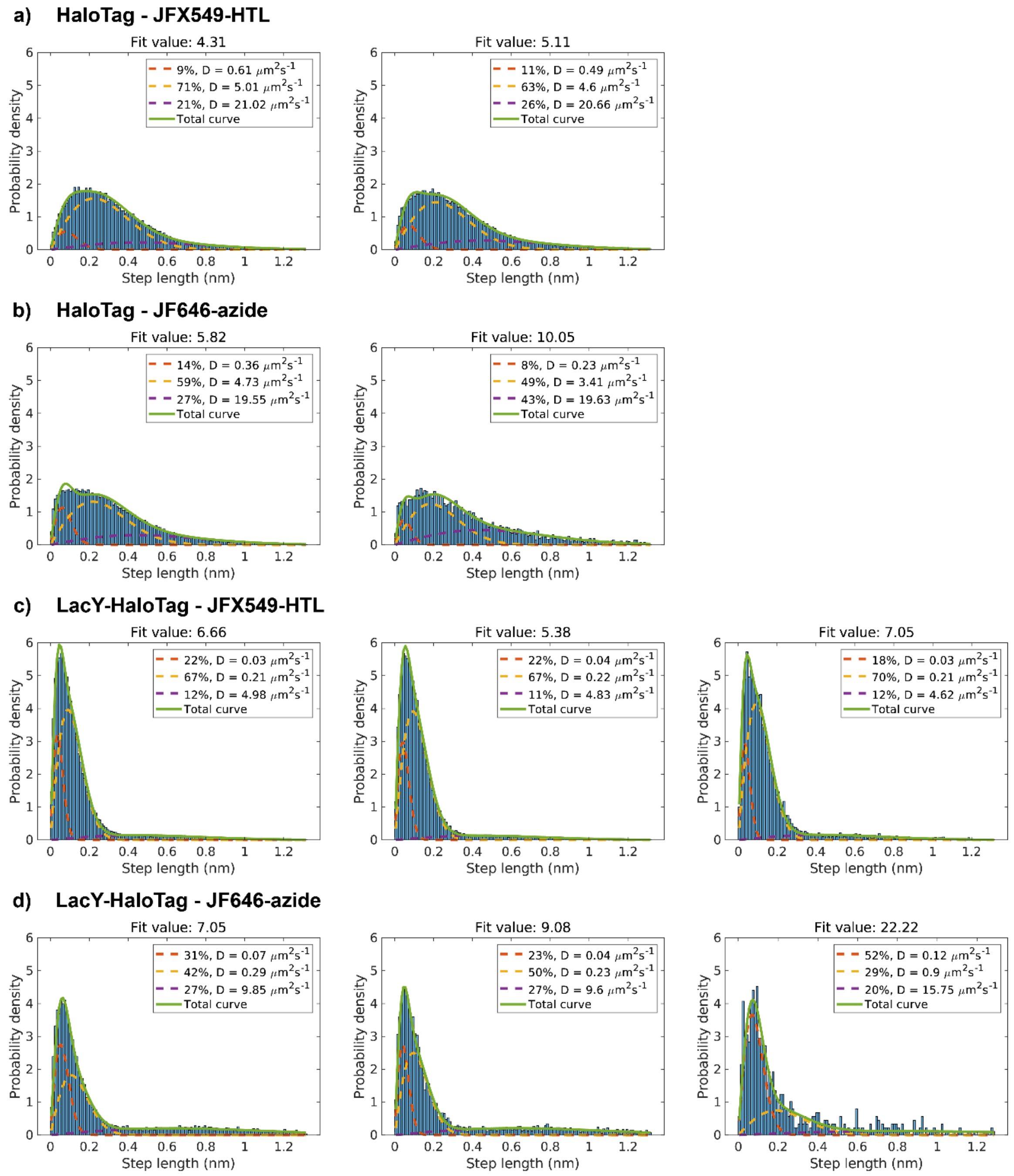

Supplementary Fig. 6. Step-length distributions of tracked particles in individual repetitions, fitted to three diffusion states. Left-most panels contain the same data as in main **Fig. 8**.

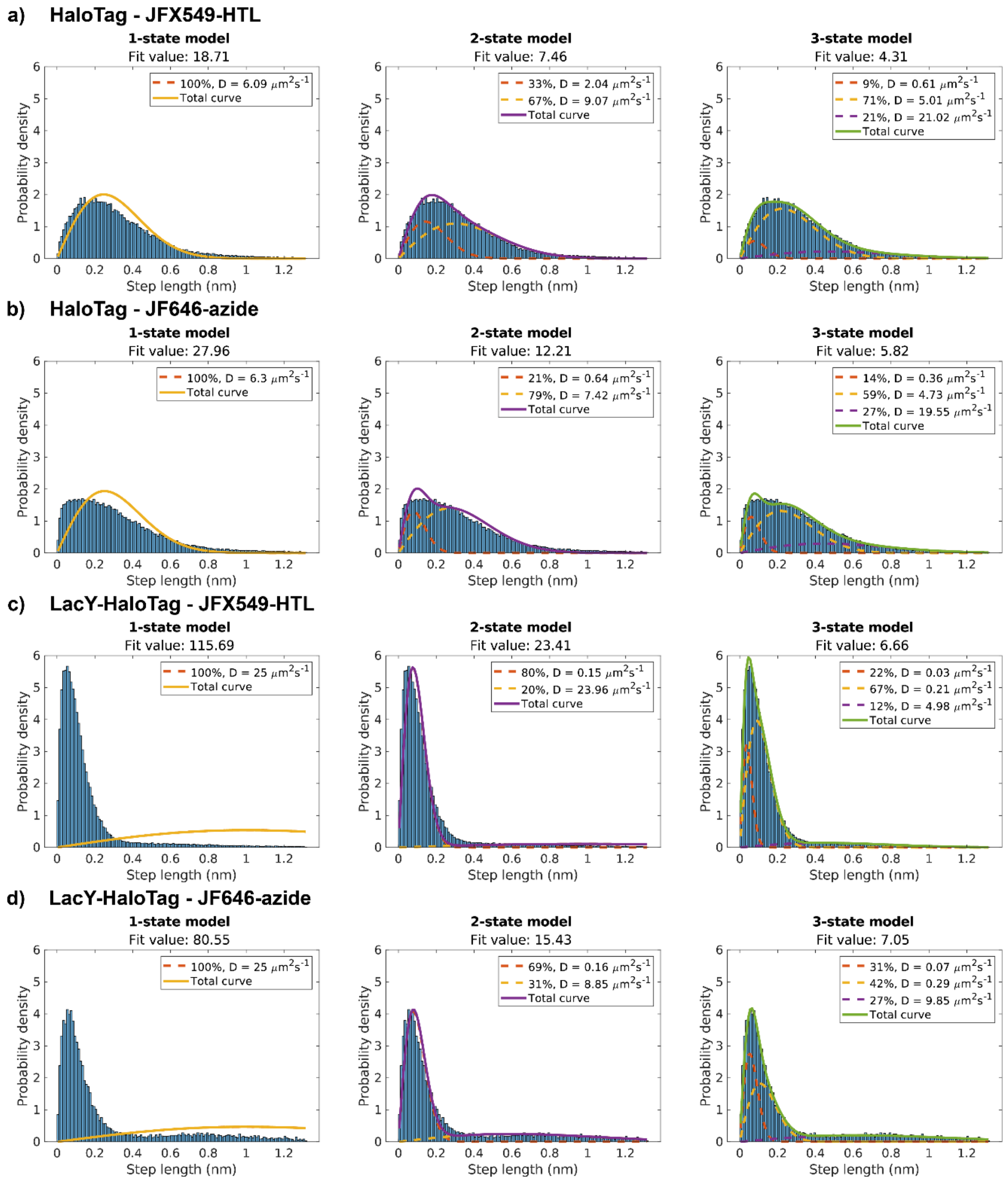

Supplementary Fig. 7. Step-length distributions from main **Fig. 8**, fitted to 1, 2, or 3 diffusion states. Data in the right-most panels are the same as in main **Fig. 8**.

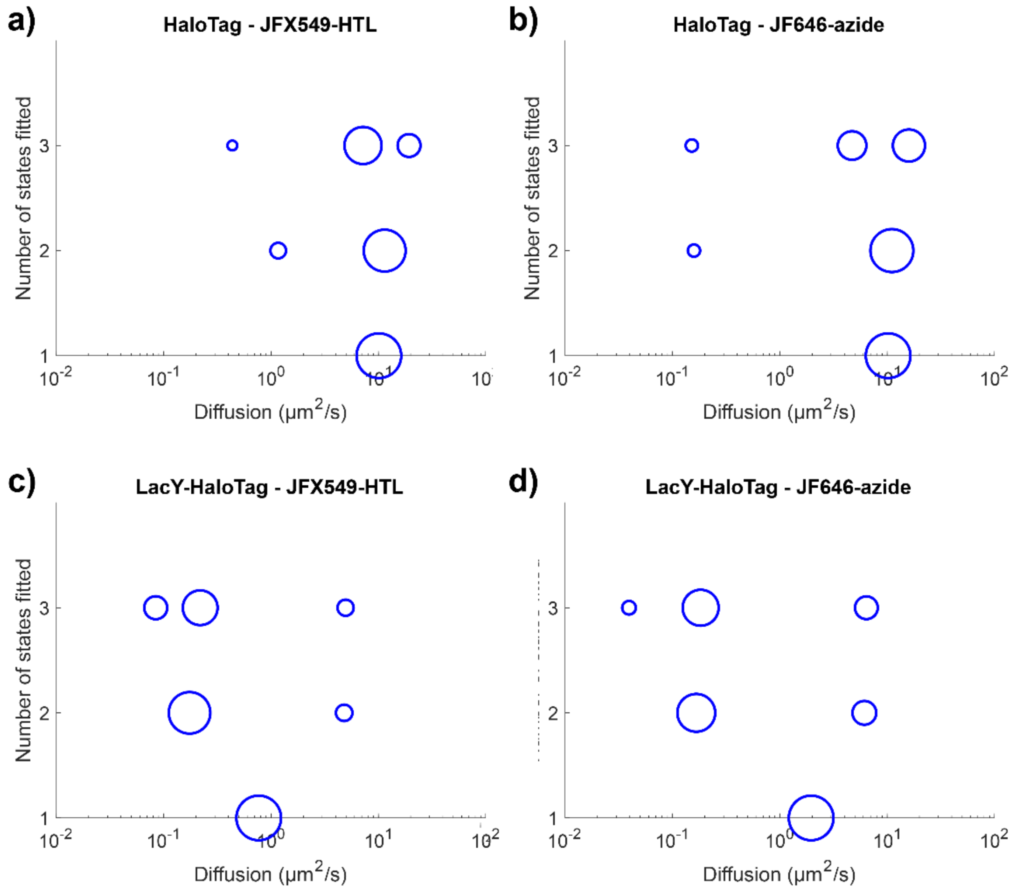

Supplementary Fig. 8. Graphical representation of HMM analysis results, showing the occupancy (area of circles) of each diffusion state when diffusion trajectories are fitted to 1, 2, or 3 discrete diffusion states. Diffusion trajectories for all repetitions in each experimental condition were combined and analyzed using an HMM algorithm that has previously been used extensively for analysis of binding kinetics in SMT data[2,31,2]. In the algorithm, the trajectories are fitted using global maximum-likelihood estimation, to a model with a pre-defined number of hidden diffusion states, where diffusion coefficients and transition frequencies between the diffusion states within trajectories are used as fitting parameters. The algorithm also accounts for motion blur, localization uncertainty, and potential missing positions in trajectories. Number of trajectory steps and repetitions (n) for each experimental condition are: (**a**) HaloTag labeled with JFX549-HTL, 99664 steps (n=2); (**b**) HaloTag labeled with JF646-azide, 99265 steps (n=2); (**c**) LacY-HaloTag labeled with JFX549-HTL, 141773 steps (n=3); (**d**) LacY-HaloTag labeled with JF646-azide, 22207 steps (n=3).

1. Brandis, G., Larsson, J. & Elf, J. Antibiotic perseverance increases the risk of resistance development. *Proc. Natl. Acad. Sci. U. S. A.* **120**, e2216216120 (2023).

2. Volkov, I. L. *et al.* tRNA tracking for direct measurements of protein synthesis kinetics in live cells. *Nat. Chem. Biol. 2018 146* **14**, 618–626 (2018).

3. Hävermark, T., Metelev, M., Lundin, E., Volkov, I. L. & Johansson, M. Dynamic binding of the bacterial chaperone Trigger factor to translating ribosomes in Escherichia coli. *Proc. Natl. Acad. Sci.* **122**, e2409536121 (2024).

Supplementary Table 1. Plasmid sequences of all constructs used in this study. Highlighted parts represent sequence-verified segments of the plasmid according to the following scheme:

Codon contest linker

Stop codon or lysine codon in tested proteins

PylRS1/PylRS2/PylRS3 sequences for OTS

tRNA[CUA] – the anticodon is underlined

<table>
<tr><th>Plasmid</th><th>Ori</th><th>Operator</th><th>Repressor</th><th>Promoter</th></tr>
<tr><td>pQE30lacIq-UAG-SCFP</td><td rowspan="2">ColE1</td><td rowspan="2">2xOsym</td><td rowspan="2">lacIq</td><td rowspan="2">T5</td></tr>
<tr><td>pQE30lacIq-AAA-SCFP</td></tr>
<tr><td>pEVOL_M.mazei-PylRS1</td><td rowspan="26">p15A</td><td rowspan="3">$AraO_1$ and $AraO_2$</td><td rowspan="3">araC</td><td rowspan="3">araBADp and glnS’</td></tr>
<tr><td>pEVOL_M.alvus-PylRS2</td></tr>
<tr><td>pEVOL_M.alvus-PylRS3</td></tr>
<tr><td>pFI1_UAG_SCFP_PylRS1</td><td rowspan="13">$AraO_1$ and $AraO_2$ for PylRS, 2 x Osym for SCFP3a</td><td rowspan="13">lacIq and araC</td><td rowspan="13">araBADp and glnS’ for PylRS, T5-Osym for SCFP3a</td></tr>
<tr><td>pFI1+</td></tr>
<tr><td>pEVOL_AAIP_G1_UAG_SCFP</td></tr>
<tr><td>pEVOL_AAIP_G1_AAA_SCFP</td></tr>
<tr><td>pEVOL_ALIP_G1_UAG_SCFP</td></tr>
<tr><td>pEVOL_ALIP_G1_AAA_SCFP</td></tr>
<tr><td>pEVOL_AF_G2_UAG_SCFP</td></tr>
<tr><td>pEVOL_AF_G2_AAA_SCFP</td></tr>
<tr><td>pEVOL_AAIP_G2_UAG_SCFP</td></tr>
<tr><td>pEVOL_AAIP_G2_AAA_SCFP</td></tr>
<tr><td>pEVOL_ALIP_G2_UAG_SCFP</td></tr>
<tr><td>pEVOL_ALIP_G2_AAA_SCFP</td></tr>
<tr><td>pEVOL_1ALIP_G1_UAG_SCFP</td></tr>
<tr><td>pMEGA_ALIP_UAG_SCFP</td><td>Osym</td><td>lacIq</td><td>Native</td></tr>
<tr><td>pASK_ALIP_UAG_SCFP</td><td>tetR/A, Osym</td><td>tetR and lacIq</td><td>Tet promoter for aaRS T5-Osym for SCFP3a</td></tr>
<tr><td>pFI4_UAG_SCFP</td><td rowspan="6">2xOsym</td><td rowspan="6">lacIq</td><td rowspan="6">aFab120 for aaRS, T5-Osym for SCFP3a</td></tr>
<tr><td>pFI4_26_UAG_SCFP</td></tr>
<tr><td>pFI4_45_UAG_SCFP</td></tr>
<tr><td>pFI4_UAG_Halo</td></tr>
<tr><td>pFI4_AAA_Halo</td></tr>
<tr><td>pFI4_LacY_UAG_SCFP</td></tr>
</table>

pQE30lacIq-UAG-SCFP and pQE30lacIq-UAG-AAA-SCFP

CTCGAGAAATCATAAAAAATTTATTTGCTTTGTGAGCGGATAACAATTATAATAGATT
CAATTGTGAGCGGATAACAATTTCACACAGAATTCATTAAAGAGGAGAAATTAACTA
TGGGCCATCATTAG/AAAAAGAATGGCGGTGCGAGCAGTAAAGGAGAAGAACTTTT
CACTGGAGTTGTCCCAATTCTTGTTGAATTAGATGGTGATGTTAATGGGCACAAATT
TTCTGTCAGTGGAGAGGGTGAAGGTGATGCAACATACGGAAAACTTACCCTTAAAT
TTATTTGCACTACTGGAAAACTACCTGTTCCATGGCCAACACTTGTCACTACTTTGA
CCTGGGGTGTTCAATGCTTTGCGAGATACCCAGATCATATGAAACAGCATGACTTTT
TCAAGAGTGCCATGCCCGAAGGTTATGTACAGGAAAGAACTATATTTTTCAAAGAT
GACGGGAACTACAAGACACGTGCTGAAGTCAAGTTTGAAGGTGATACCCTTGTTAA
TAGAATCGAGTTAAAAGGTATTGATTTTAAAGAAGATGGAAACATTCTTGGACACAA
ATTGGAATACAACTATATCTCAGACAATGTATACATCACGGCAGACAAACAAAAGA
ATGGAATCAAAGCGAACTTCAAAATTAGACACAACATTGAAGATGGAGGTGTTCAA
CTAGCAGACCATTATCAACAAAATACTCCAATTGGCGATGGCCCTGTCCTTTTACCA
GACAACCATTACCTGTCCACCCAATCTAAGCTCTCGAAAGATCCCAACGAAAAGAG
AGACCACATGGTCCTTCTTGAGTTTGTAACAGCTGCTGGGATTACACATGGCATGG
ATGAACTATACAAATAAGACTCCTGTTGATAGATCCAGTAATGACCTCAGAACTCCA
TCTGGATTTGTTCAGAACGCTCGGTTGCCGCCGGGCGTTTTTTATTGGTGAGAATCC
AAGCTAGCTTGGCGAGATTTTCAGGAGCTAAGGAAGCTAAATTTTTTTAAGGCAGTT
ATTGGTGCCCTTAAACGCCTGGGGTAATGACTCTCTAGCTTGAGGCATCAAATAAA
ACGAAAGGCTCAGTCGAAAGACTGGGCCTTTCGTTTTATCTGTTGTTTGTCGGTGAA
CGCTCTCCTGAGTAGGACAAATCCGCCCTCTAGACTGGGTTGAAGGCTCTCAAGGG
CATCGGTCGAGATCCCGGTGCCTAATGAGTGAGCTAACTTACATTAATTGCGTTGC
GCTCACTGCCCGCTTTCCAGTCGGGAAACCTGTCGTGCCAGCTGCATTAATGAATC
GGCCAACGCGCGGGGAGAGGCGGTTTGCGTATTGGGCGCCAGGGTGGTTTTTCTTT
TCACCAGTGAGACGGGCAACAGCTGATTGCCCTTCACCGCCTGGCCCTGAGAGAG
TTGCAGCAAGCGGTCCACGCTGGTTTGCCCCAGCAGGCGAAAATCCTGTTTGATGG
TGGTTAACGGCGGGATATAACATGAGCTGTCTTCGGTATCGTCGTATCCCACTACC
GAGATATCCGCACCAACGCGCAGCCCGGACTCGGTAATGGCGCGCATTGCGCCCA
GCGCCATCTGATCGTTGGCAACCAGCATCGCAGTGGGAACGATGCCCTCATTCAGC
ATTTGCATGGTTTGTTGAAAACCGGACATGGCACTCCAGTCGCCTTCCCGTTCCGCT
ATCGGCTGAATTTGATTGCGAGTGAGATATTTATGCCAGCCAGCCAGACGCAGACG
CGCCGAGACAGAACTTAATGGGCCCGCTAACAGCGCGATTTGCTGGTGACCCAAT
GCGACCAGATGCTCCACGCCCAGTCGCGTACCGTCTTCATGGGAGAAAATAATACT
GTTGATGGGTGTCTGGTCAGAGACATCAAGAAATAACGCCGGAACATTAGTGCAGG
CAGCTTCCACAGCAATGGCATCCTGGTCATCCAGCGGATAGTTAATGATCAGCCCA
CTGACGCGTTGCGCGAGAAGATTGTGCACCGCCGCTTTACAGGCTTCGACGCCGCT
TCGTTCTACCATCGACACCACCACGCTGGCACCCAGTTGATCGGCGCGAGATTTAA
TCGCCGCGACAATTTGCGACGGCGCGTGCAGGGCCAGACTGGAGGTGGCAACGCC
AATCAGCAACGACTGTTTGCCCGCCAGTTGTTGTGCCACGCGGTTGGGAATGTAAT
TCAGCTCCGCCATCGCCGCTTCCACTTTTTCCCGCGTTTTCGCAGAAACGTGGCTGG
CCTGGTTCACCACGCGGGAAACGGTCTGATAAGAGACACCGGCATACTCTGCGAC
ATCGTATAACGTTACTGGTTTCACATTCACCACCCTGAATTGACTCTCTTCCGGGCG
CTATCATGCCATACCGCGAAAGGTTTTGCACCATTCGATGGTGTCCGGGATCTCGA
CGCTCTCCCTTATGCGACTCCTGCATTAGGAAGCAGCCGCTGCCTCGCGCGTTTCG
GTGATGACGGTGAAAACCTCTGACACATGCAGCTCCCGGAGACGGTCACAGCTTGT
CTGTAAGCGGATGCCGGGAGCAGACAAGCCCGTCAGGGCGCGTCAGCGGGTGTTG
GCGGGTGTCGGGGCGCAGCCATGACCCAGTCACGTAGCGATAGCGGAGTGTATAC
TGGCTTAACTATGCGGCATCAGAGCAGATTGTACTGAGAGTGCACCATATGCGGTG
TGAAATACCGCACAGATGCGTAAGGAGAAAATACCGCATCAGGCGCTCTTCCGCTT
CCTCGCTCACTGACTCGCTGCGCTCGGTCGTTCGGCTGCGGCGAGCGGTATCAGCT
CACTCAAAGGCGGTAATACGGTTATCCACAGAATCAGGGGATAACGCAGGAAAGA
ACATGTGAGCAAAAGGCCAGCAAAAGGCCAGGAACCGTAAAAAGGCCGCGTTGCT
GGCGTTTTTCCATAGGCTCCGCCCCCCTGACGAGCATCACAAAAATCGACGCTCAA
GTCAGAGGTGGCGAAACCCGACAGGACTATAAAGATACCAGGCGTTTCCCCCTGG
AAGCTCCCTCGTGCGCTCTCCTGTTCCGACCCTGCCGCTTACCGGATACCTGTCCG

**CCTTTCTCCCTTCGGGAAGCGTGGCGCTTTCTCATAGCTCACGCTGTAGGTATCTCA
GTTCGGTGTAGGTCGTTCGCTCCAAGCTGGGCTGTGTGCACGAACCCCCCGTTCAG
CCCGACCGCTGCGCCTTATCCGGTAACTATCGTCTTGAGTCCAACCCGGTAAGACA
CGACTTATCGCCACTGGCAGCAGCCACTGGTAACAGGATTAGCAGAGCGAGGTAT
GTAGGCGGTGCTACAGAGTTCTTGAAGTGGTGGCCTAACTACGGCTACACTAGAAG
GACAGTATTTGGTATCTGCGCTCTGCTGAAGCCAGTTACCTTCGGAAAAAGAGTTG
GTAGCTCTTGATCCGGCAAACAAACCACCGCTGGTAGCGGTGGTTTTTTTGTTTGCA
AGCAGCAGATTACGCGCAGAAAAAAAGGATCTCAAGAAGATCCTTTGATCTTTTCT
ACGGGGTCTGACGCTCAGTGGAACGAAAACTCACGTTAAGGGATTTTGGTCATGAG
ATTATCAAAAAGGATCTTCACCTAGATCCTTTTAAATTAAAAATGAAGTTTTAAATCA
ATCTAAAGTATATATGAGTAAACTTGGTCTGACAGTTAGAAAAACTCATCGAGCATC
AAATGAAACTGCAATTTATTCATATCAGGATTATCAATACCATATTTTTGAAAAAGCC
GTTTCTGTAATGAAGGAGAAAACTCACCGAGGCAGTTCCATAGGATGGCAAGATCC
TGGTATCGGTCTGCGATTCCGACTCGTCCAACATCAATACAACCTATTAATTTCCCC
TCGTCAAAAATAAGGTTATCAAGTGAGAAATCACCATGAGTGACGACTGAATCCGG
TGAGAATGGCAAAAGTTTATGCATTTCTTTCCAGACTTGTTCAACAGGCCAGCCATT
ACGCTCGTCATCAAAATCACTCGCATCAACCAAACCGTTATTCATTCGTGATTGCGC
CTGAGCGAGACGAAATACGCGGTCGCTGTTAAAAGGACAATTACAAACAGGAATC
GAATGCAACCGGCGCAGGAACACTGCCAGCGCATCAACAATATTTTCACCTGAATC
AGGATATTCTTCTAATACCTGGAATGCTGTTTTCCCGGGGATCGCAGTGGTGAGTAA
CCATGCATCATCAGGAGTACGGATAAAATGCTTGATGGTCGGAAGAGGCATAAATT
CCGTCAGCCAGTTTAGTCTGACCATCTCATCTGTAACATCATTGGCAACGCTACCTT
TGCCATGTTTCAGAAACAACTCTGGCGCATCGGGCTTCCCATACAATCGATAGATTG
TCGCACCTGATTGCCCGACATTATCGCGAGCCCATTTATACCCATATAAATCAGCAT
CCATGTTGGAATTTAATCGCGGCCTAGAGCAAGACGTTTCCCGTTGAATATGGCTCA
TACTCTTCCTTTTTCAATATTATTGAAGCATTTATCAGGGTTATTGTCTCATGAGCGG
ATACATATTTGAATGTATTTAGAAAAATAAACAAATAGGGGTTCCGCGCACATTTCC
CCGAAAAGTGCCACCTGACGTCTAAGAAACCATTATTATCATGACATTAACCTATAA
AAATAGGCGTATCACGAGGCCCTTTCGTCTTCAC**

pQE30lacIq-26UAG-SCFP and pQE30lacIq-45UAG -SCFP

**CTCGAGAAATCATAAAAAATTTATTTGCTTTGTGAGCGGATAACAATTATAATAGA
TTCAATTGTGAGCGGATAACAATTTCACACAGAATTCATTAAAGAGGAGAAATTAA
CTATGAGTAAAGGAGAAGAACTTTTCACTGGAGTTGTCCCAATTCTTGTTGAATTA
GATGGTGATGTTAATGGGCAC<u>AAA/UAG</u>TTTTCTGTCAGTGGAGAGGGTGAAGGT
GATGCAACATACGGAAAACTTACCCTT<u>AAA/UAG</u>TTTATTTGCACTACTGGAAAAC
TACCTGTTCCATGGCCAACACTTGTCACTACTTTGACCTGGGGTGTTCAATGCTTT
GCGAGATACCCAGATCATATGAAACAGCATGACTTTTTCAAGAGTGCCATGCCCG
AAGGTTATGTACAGGAAAGAACTATATTTTTCAAAGATGACGGGAACTACAAGAC
ACGTGCTGAAGTCAAGTTTGAAGGTGATACCCTTGTTAATAGAATCGAGTTAAAA
GGTATTGATTTTAAAGAAGATGGAAACATTCTTGGACACAAATTGGAATACAACTA
TATCTCAGACAATGTATACATCACGGCAGACAAACAAAAGAATGGAATCAAAGCG
AACTTCAAAATTAGACACAACATTGAAGATGGAGGTGTTCAACTAGCAGACCATT
ATCAACAAAATACTCCAATTGGCGATGGCCCTGTCCTTTTACCAGACAACCATTAC
CTGTCCACCCAATCTAAGCTCTCGAAAGATCCCAACGAAAAGAGAGACCACATGG
TCCTTCTTGAGTTTGTAACAGCTGCTGGGATTACACATGGCATGGATGAACTATAC
AAATAAGACTCCTGTTGATAGATCCAGTAATGACCTCAGAACTCCATCTGGATTTG**

**TTCAGAACGCTCGGTTGCCGCCGGGCGTTTTTTATTGGTGAGAATCCAAGCTAGC
TTGGCGAGATTTTCAGGAGCTAAGGAAGCTAAATTTTTTTAAGGCAGTTATTGGT
GCCCTTAAACGCCTGGGGTAATGACTCTCTAGCTTGAGGCATCAAATAAAACGAA
AGGCTCAGTCGAAAGACTGGGCCTTTCGTTTTATCTGTTGTTTGTCGGTGAACGC
TCTCCTGAGTAGGACAAATCCGCCCTCTAGACTGGGTTGAAGGCTCTCAAGGGCA
TCGGTCGAGATCCCGGTGCCTAATGAGTGAGCTAACTTACATTAATTGCGTTGCG
CTCACTGCCCGCTTTCCAGTCGGGAAACCTGTCGTGCCAGCTGCATTAATGAATC
GGCCAACGCGCGGGGAGAGGCGGTTTGCGTATTGGGCGCCAGGGTGGTTTTTCT
TTTCACCAGTGAGACGGGCAACAGCTGATTGCCCTTCACCGCCTGGCCCTGAGAG
AGTTGCAGCAAGCGGTCCACGCTGGTTTGCCCCAGCAGGCGAAAATCCTGTTTGA
TGGTGGTTAACGGCGGGATATAACATGAGCTGTCTTCGGTATCGTCGTATCCCAC
TACCGAGATATCCGCACCAACGCGCAGCCCGGACTCGGTAATGGCGCGCATTGCG
CCCAGCGCCATCTGATCGTTGGCAACCAGCATCGCAGTGGGAACGATGCCCTCAT
TCAGCATTTGCATGGTTTGTTGAAAACCGGACATGGCACTCCAGTCGCCTTCCCG
TTCCGCTATCGGCTGAATTTGATTGCGAGTGAGATATTTATGCCAGCCAGCCAGA
CGCAGACGCGCCGAGACAGAACTTAATGGGCCCGCTAACAGCGCGATTTGCTGGT
GACCCAATGCGACCAGATGCTCCACGCCCAGTCGCGTACCGTCTTCATGGGAGAA
AATAATACTGTTGATGGGTGTCTGGTCAGAGACATCAAGAAATAACGCCGGAACA
TTAGTGCAGGCAGCTTCCACAGCAATGGCATCCTGGTCATCCAGCGGATAGTTAA
TGATCAGCCCACTGACGCGTTGCGCGAGAAGATTGTGCACCGCCGCTTTACAGGC
TTCGACGCCGCTTCGTTCTACCATCGACACCACCACGCTGGCACCCAGTTGATCG
GCGCGAGATTTAATCGCCGCGACAATTTGCGACGGCGCGTGCAGGGCCAGACTG
GAGGTGGCAACGCCAATCAGCAACGACTGTTTGCCCGCCAGTTGTTGTGCCACGC
GGTTGGGAATGTAATTCAGCTCCGCCATCGCCGCTTCCACTTTTTCCCGCGTTTTC
GCAGAAACGTGGCTGGCCTGGTTCACCACGCGGGAAACGGTCTGATAAGAGACA
CCGGCATACTCTGCGACATCGTATAACGTTACTGGTTTCACATTCACCACCCTGAA
TTGACTCTCTTCCGGGCGCTATCATGCCATACCGCGAAAGGTTTTGCACCATTCG
ATGGTGTCCGGGATCTCGACGCTCTCCCTTATGCGACTCCTGCATTAGGAAGCAG
CCGCTGCCTCGCGCGTTTCGGTGATGACGGTGAAAACCTCTGACACATGCAGCTC
CCGGAGACGGTCACAGCTTGTCTGTAAGCGGATGCCGGGAGCAGACAAGCCCGT
CAGGGCGCGTCAGCGGGTGTTGGCGGGTGTCGGGGCGCAGCCATGACCCAGTCA
CGTAGCGATAGCGGAGTGTATACTGGCTTAACTATGCGGCATCAGAGCAGATTGT
ACTGAGAGTGCACCATATGCGGTGTGAAATACCGCACAGATGCGTAAGGAGAAAA
TACCGCATCAGGCGCTCTTCCGCTTCCTCGCTCACTGACTCGCTGCGCTCGGTCG
TTCGGCTGCGGCGAGCGGTATCAGCTCACTCAAAGGCGGTAATACGGTTATCCAC
AGAATCAGGGGATAACGCAGGAAAGAACATGTGAGCAAAAGGCCAGCAAAAGGC
CAGGAACCGTAAAAAGGCCGCGTTGCTGGCGTTTTTCCATAGGCTCCGCCCCCCT
GACGAGCATCACAAAAATCGACGCTCAAGTCAGAGGTGGCGAAACCCGACAGGA
CTATAAAGATACCAGGCGTTTCCCCCTGGAAGCTCCCTCGTGCGCTCTCCTGTTC
CGACCCTGCCGCTTACCGGATACCTGTCCGCCTTTCTCCCTTCGGGAAGCGTGGC
GCTTTCTCATAGCTCACGCTGTAGGTATCTCAGTTCGGTGTAGGTCGTTCGCTCCA
AGCTGGGCTGTGTGCACGAACCCCCCGTTCAGCCCGACCGCTGCGCCTTATCCGG
TAACTATCGTCTTGAGTCCAACCCGGTAAGACACGACTTATCGCCACTGGCAGCA
GCCACTGGTAACAGGATTAGCAGAGCGAGGTATGTAGGCGGTGCTACAGAGTTCT
TGAAGTGGTGGCCTAACTACGGCTACACTAGAAGGACAGTATTTGGTATCTGCGC
TCTGCTGAAGCCAGTTACCTTCGGAAAAAGAGTTGGTAGCTCTTGATCCGGCAAA
CAAACCACCGCTGGTAGCGGTGGTTTTTTTGTTTGCAAGCAGCAGATTACGCGCA
GAAAAAAAGGATCTCAAGAAGATCCTTTGATCTTTTCTACGGGGTCTGACGCTCA
GTGGAACGAAAACTCACGTTAAGGGATTTTGGTCATGAGATTATCAAAAAGGATC
TTCACCTAGATCCTTTTAAATTAAAAATGAAGTTTTAAATCAATCTAAAGTATATAT
GAGTAAACTTGGTCTGACAGTTAGAAAAACTCATCGAGCATCAAATGAAACTGCA
ATTTATTCATATCAGGATTATCAATACCATATTTTTGAAAAAGCCGTTTCTGTAAT
GAAGGAGAAAACTCACCGAGGCAGTTCCATAGGATGGCAAGATCCTGGTATCGGT
CTGCGATTCCGACTCGTCCAACATCAATACAACCTATTAATTTCCCCTCGTCAAAA
ATAAGGTTATCAAGTGAGAAATCACCATGAGTGACGACTGAATCCGGTGAGAATG**

**GCAAAAGTTTATGCATTTCTTTCCAGACTTGTTCAACAGGCCAGCCATTACGCTCG**
**TCATCAAAATCACTCGCATCAACCAAACCGTTATTCATTCGTGATTGCGCCTGAGC**
**GAGACGAAATACGCGGTCGCTGTTAAAAGGACAATTACAAACAGGAATCGAATGC**
**AACCGGCGCAGGAACACTGCCAGCGCATCAACAATATTTTCACCTGAATCAGGAT**
**ATTCTTCTAATACCTGGAATGCTGTTTTCCCGGGGATCGCAGTGGTGAGTAACCA**
**TGCATCATCAGGAGTACGGATAAAATGCTTGATGGTCGGAAGAGGCATAAATTCC**
**GTCAGCCAGTTTAGTCTGACCATCTCATCTGTAACATCATTGGCAACGCTACCTTT**
**GCCATGTTTCAGAAACAACTCTGGCGCATCGGGCTTCCCATACAATCGATAGATT**
**GTCGCACCTGATTGCCCGACATTATCGCGAGCCCATTTATACCCATATAAATCAGC**
**ATCCATGTTGGAATTTAATCGCGGCCTAGAGCAAGACGTTTCCCGTTGAATATGG**
**CTCATACTCTTCCTTTTTCAATATTATTGAAGCATTTATCAGGGTTATTGTCTCATG**
**AGCGGATACATATTTGAATGTATTTAGAAAAATAAACAAATAGGGGTTCCGCGCA**
**CATTTCCCCGAAAAGTGCCACCTGACGTCTAAGAAACCATTATTATCATGACATTA**
**ACCTATAAAAATAGGCGTATCACGAGGCCCTTTCGTCTTCAC**

**pEVOL_M.mazei-PylRS1**
**TCCTGAAAATCTCGATAACTCAAAAAATACGCCCGGTAGTGATCTTATTTCATTAT**
**GGTGAAAGTTGGAACCTCTTACGTGCCGATCAACGTCTCATTTTCGCCAAAAGTT**
**GGCCCAGGGCTTCCCGGTATCAACAGGGACACCAGGATTTATTTATTCTGCGAAG**
**TGATCTTCCGTCACAGGTATTTATTCGGCGCAAAGTGCGTCGGGTGATGCTGCCA**
**ACTTACTGATTTAGTGTATGATGGTGTTTTTGAGGTGCTCCAGTGGCTTCTGTTTC**
**TATCAGCTGTCCCTCCTGTTCAGCTACTGACGGGGTGGTGCGTAACGGCAAAAGC**
**ACCGCCGGACATCAGCGCTAGCGGAGTGTATACTGGCTTACTATGTTGGCACTGA**
**TGAGGGTGTCAGTGAAGTGCTTCATGTGGCAGGAGAAAAAAGGCTGCACCGGTG**
**CGTCAGCAGAATATGTGATACAGGATATATTCCGCTTCCTCGCTCACTGACTCGCT**
**ACGCTCGGTCGTTCGACTGCGGCGAGCGGAAATGGCTTACGAACGGGGCGGAGA**
**TTTCCTGGAAGATGCCAGGAAGATACTTAACAGGGAAGTGAGAGGGCCGCGGCA**
**AAGCCGTTTTTCCATAGGCTCCGCCCCCCTGACAAGCATCACGAAATCTGACGCT**
**CAAATCAGTGGTGGCGAAACCCGACAGGACTATAAAGATACCAGGCGTTTCCCCC**
**TGGCGGCTCCCTCGTGCGCTCTCCTGTTCCTGCCTTTCGGTTTACCGGTGTCATTC**
**CGCTGTTATGGCCGCGTTTGTCTCATTCCACGCCTGACACTCAGTTCCGGGTAGG**
**CAGTTCGCTCCAAGCTGGACTGTATGCACGAACCCCCCGTTCAGTCCGACCGCTG**
**CGCCTTATCCGGTAACTATCGTCTTGAGTCCAACCCGGAAAGACATGCAAAAGCA**
**CCACTGGCAGCAGCCACTGGTAATTGATTTAGAGGAGTTAGTCTTGAAGTCATGC**
**GCCGGTTAAGGCTAAACTGAAAGGACAAGTTTTGGTGACTGCGCTCCTCCAAGCC**
**AGTTACCTCGGTTCAAAGAGTTGGTAGCTCAGAGAACCTTCGAAAAACCGCCCTG**
**CAAGGCGGTTTTTTCGTTTTCAGAGCAAGAGATTACGCGCAGACCAAAACGATCT**
**CAAGAAGATCATCTTATTAATCAGATAAAATATTTCTAGATTTCAGTGCAATTTAT**
**CTCTTCAAATGTAGCACCTGAAGTCAGCCCCATACGATATAAGTTGTAATTCTCAT**
**GTGGTACCCAATTATGACAACTTGACGGCTACATCATTCACTTTTTCTTCACAACC**
**GGCACGGAACTCGCTCGGGCTGGCCCCGGTGCATTTTTTAAATACCCGCGAGAAA**
**TAGAGTTGATCGTCAAAACCAACATTGCGACCGACGGTGGCGATAGGCATCCGGG**
**TGGTGCTCAAAAGCAGCTTCGCCTGGCTGATACGTTGGTCCTCGCGCCAGCTTAA**
**GACGCTAATCCCTAACTGCTGGCGGAAAAGATGTGACAGACGCGACGGCGACAA**
**GCAAACATGCTGTGCGACGCTGGCGATATCAAAATTGCTGTCTGCCAGGTGATCG**
**CTGATGTACTGACAAGCCTCGCGTACCCGATTATCCATCGGTGGATGGAGCGACT**
**CGTTAATCGCTTCCATGCGCCGCAGTAACAATTGCTCAAGCAGATTTATCGCCAG**
**CAGCTCCGAATAGCGCCCTTCCCCTTGCCCGGCGTTAATGATTTGCCCAAACAGG**
**TCGCTGAAATGCGGCTGGTGCGCTTCATCCGGGCGAAAGAACCCCGTATTGGCAA**
**ATATTGACGGCCAGTTAAGCCATTCATGCCAGTAGGCGCGCGGACGAAAGTAAAC**
**CCACTGGTGATACCATTCGCGAGCCTCCGGATGACGACCGTAGTGATGAATCTCT**
**CCTGGCGGGAACAGCAAAATATCACTCGGTCGGCAAACAAATTCTCGTCCCTGAT**
**TTTTCACCACCCCCTGACCGCGAATGGTGAGATTGAGAATATAACCTTTCATTCCC**
**AGCGGTCGGTCGATAAAAAAATCGAGATAACCGTTGGCCTCAATCGGCGTTAAAC**

CCGCCACCAGATGGGCATTAAACGAGTATCCCGGCAGCAGGGGATCATTTTGCGC
TTCAGCCATACTTTTCATACTCCCGCCATTCAGAGAAGAAACCAATTGTCCATATT
GCATCAGACATTGCCGTCACTGCGTCTTTTACTGGCTCTTCTCGCTAACCAAACCG
GTAACCCCGCTTATTAAAAGCATTCTGTAACAAAGCGGGACCAAAGCCATGACAA
AAACGCGTAACAAAAGTGTCTATAATCACGGCAGAAAAGTCCACATTGATTATTT
GCACGGCGTCACACTTTGCTATGCCATAGCATTTTTATCCATAAGATTAGCGGATC
CTACCTGACGCTTTTTATCGCAACTCTCTACTGTTTCTCCATACCCGTTTTTTTGG
GCTAACAGGAGGAATTAGATCTATGGACAAAAAACCGCTGAATACCCTGATCTCT
GCTACTGGTCTGTGGATGAGTCGTACCGGAACCATTCATAAAATCAAACACCACG
AGGTTAGCCGTTCGAAAATCTATATTGAGATGGCGTGTGGCGATCATCTGGTTGT
GAACAATAGCCGCTCTTCTCGTACAGCACGTGCACTGCGTCACCACAAATATCGT
AAAACCTGTAAACGTTGCCGTGTGTCCGATGAGGATCTGAACAAATTCCTGACAA
AAGCCAATGAGGACCAAACAAGCGTGAAAGTGAAAGTCGTTAGCGCTCCTACCCG
TACTAAAAAAGCAATGCCGAAATCCGTTGCTCGTGCCCCTAAACCACTGGAAAAC
ACTGAAGCAGCACAGGCACAGCCGTCTGGAAGCAAATTCTCTCCGGCCATTCCTG
TTTCTACCCAGGAGTCCGTTTCTGTTCCAGCAAGTGTGAGCACCAGCATTAGCAG
TATTAGCACCGGTGCCACCGCTAGCGCCCTGGTTAAAGGCAATACCAATCCGATT
ACAAGCATGTCTGCCCCGGTTCAAGCATCAGCTCCAGCACTGACAAAATCCCAAA
CCGATCGTCTGGAGGTTCTGCTGAATCCGAAAGACGAAATCAGCCTGAATTCCGG
CAAACCGTTTCGTGAACTGGAGAGCGAACTGCTGTCACGTCGTAAAAAAGACCTG
CAACAAATCTATGCCGAAGAACGTGAGAACTATCTGGGGAAACTGGAACGTGAAA
TCACCCGCTTTTTCGTGGATCGTGGCTTTCTGGAGATCAAATCCCCGATTCTGATT
CCTCTGGAGTATATCGAGCGTATGGGCATCGACAATGATACCGAACTGAGCAAAC
AAATTTTCCGTGTGGATAAAAACTTCTGTCTGCGCCCTATGCTGGCACCAAATCTG
GCTAACTATCTGCGCAAACTGGACCGTGCCCTGCCTGATCCTATCAAAATCTTCG
AGATCGGCCCGTGTTATCGTAAAGAGTCCGACGGTAAAGAACATCTGGAGGAGTT
TACCATGCTGAACTTTTGCCAAATGGGTTCAGGTTGTACTCGTGAGAACCTGGAA
AGCATCATCACCGATTTTCTGAACCACCTGGGCATTGACTTCAAAATTGTGGGCG
ACAGCTGTATGGTGTTTGGCGACACCCTGGATGTCATGCACGGCGACCTGGAACT
GTCTAGTGCCGTTGTTGGACCAATTCCGCTGGACCGTGAGTGGGGTATCGACAAA
CCGTGGATCGGAGCAGGATTCGGTCTGGAACGCCTGCTGAAAGTGAAACACGACT
TCAAAAACATCAAACGTGCCGCCCGTTCTGAATCGTATTATAACGGGATCTCTAC
GAACCTGTAAGTCGACCATCATCATCATCATCATTGAGTTTAAACGGTCTCCAGCT
TGGCTGTTTTGGCGGATGAGAGAAGATTTTCAGCCTGATACAGATTAAATCAGAA
CGCAGAAGCGGTCTGATAAAACAGAATTTGCCTGGCGGCAGTAGCGCGGTGGTC
CCACCTGACCCCATGCCGAACTCAGAAGTGAAACGCCGTAGCGCCGATGGTAGTG
TGGGGTCTCCCCATGCGAGAGTAGGGAACTGCCAGGCATCAAATAAAACGAAAG
GCTCAGTCGAAAGACTGGGCCTTGTTTGTGAGCTCCCGGTCATCAATCATCCCCA
TAATCCTTGTTAGATTATCAATTTTAAAAAACTAACAGTTGTCAGCCTGTCCCGCT
TTAATATCATACGCCGTTATACGTTGTTTACGCTTTGAGGAATCCCATATGGACAA
AAAACCGCTGAATACCCTGATCTCTGCTACTGGTCTGTGGATGAGTCGTACCGGA
ACCATTCATAAAATCAAACACCACGAGGTTAGCCGTTCGAAAATCTATATTGAGAT
GGCGTGTGGCGATCATCTGGTTGTGAACAATAGCCGCTCTTCTCGTACAGCACGT
GCACTGCGTCACCACAAATATCGTAAAACCTGTAAACGTTGCCGTGTGTCCGATG
AGGATCTGAACAAATTCCTGACAAAAGCCAATGAGGACCAAACAAGCGTGAAAGT
GAAAGTCGTTAGCGCTCCTACCCGTACTAAAAAAGCAATGCCGAAATCCGTTGCT
CGTGCCCCTAAACCACTGGAAAACACTGAAGCAGCACAGGCACAGCCGTCTGGAA
GCAAATTCTCTCCGGCCATTCCTGTTTCTACCCAGGAGTCCGTTTCTGTTCCAGCA
AGTGTGAGCACCAGCATTAGCAGTATTAGCACCGGTGCCACCGCTAGCGCCCTGG
TTAAAGGCAATACCAATCCGATTACAAGCATGTCTGCCCCGGTTCAAGCATCAGC
TCCAGCACTGACAAAATCCCAAACCGATCGTCTGGAGGTTCTGCTGAATCCGAAA
GACGAAATCAGCCTGAATTCCGGCAAACCGTTTCGTGAACTGGAGAGCGAACTGC
TGTCACGTCGTAAAAAAGACCTGCAACAAATCTATGCCGAAGAACGTGAGAACTA
TCTGGGGAAACTGGAACGTGAAATCACCCGCTTTTTCGTGGATCGTGGCTTTCTG
GAGATCAAATCCCCGATTCTGATTCCTCTGGAGTATATCGAGCGTATGGGCATCG

ACAATGATACCGAACTGAGCAAACAAATTTTCCGTGTGGATAAAAACTTCTGTCTG
CGCCCTATGCTGGCACCAAATCTGGCTAACTATCTGCGCAAACTGGACCGTGCCC
TGCCTGATCCTATCAAAATCTTCGAGATCGGCCCGTGTTATCGTAAAGAGTCCGA
CGGTAAAGAACATCTGGAGGAGTTTACCATGCTGAACTTTTGCCAAATGGGTTCA
GGTTGTACTCGTGAGAACCTGGAAAGCATCATCACCGATTTTCTGAACCACCTGG
GCATTGACTTCAAAATTGTGGGCGACAGCTGTATGGTGTTTGGCGACACCCTGGA
TGTCATGCACGGCGACCTGGAACTGTCTAGTGCCGTTGTTGGACCAATTCCGCTG
GACCGTGAGTGGGGTATCGACAAACCGTGGATCGGAGCAGGATTCGGTCTGGAA
CGCCTGCTGAAAGTGAAACACGACTTCAAAAACATCAAACGTGCCGCCCGTTCTG
AATCGTATTATAACGGGATCTCTACGAACCTGTAACTGCAGTTTCAAACGCTAAAT
TGCCTGATGCGCTACGCTTATCAGGCCTACATGATCTCTGCAATATATTGAGTTTG
CGTGCTTTTGTAGGCCGGATAAGGCGTTCACGCCGCATCCGGCAAGAAACAGCAA
ACAATCCAAAACGCCGCGTTCAGCGGCGTTTTTTCTGCTTTTCTTCGCGAATTAAT
TCCGCTTCGCAACATGTGAGCACCGGTTTATTGACTACCGGAAGCAGTGTGACCG
TGTGCTTCTCAAATGCCTGAGGCCAGTTTGCTCAGGCTCTCCCCGTGGAGGTAAT
AATTGACGATATGATCAGTGCACGGCTAACTAAGCGGCCTGCTGACTTTCTCGCC
GATCAAAAGGCATTTTGCTATTAAGGGATTGACGAGGGCGTATCTGCGCAGTAAG
ATGCGCCCCGCATTGGAAACCTGATCATGTAGATCGAATGGACTCTAAATCCGTT
CAGCCGGGTTAGATTCCCGGGGTTTCCGCCAAATTCGAAAAGCCTGCTCAACGAG
CAGGCTTTTTTGCATGCTCGAGCAGCTCAGGGTCGAATTTGCTTTCGAATTTCTGC
CATTCATCCGCTTATTATCACTTATTCAGGCGTAGCACCAGGCGTTTAAGGGCACC
AATAACTGCCTTAAAAAAATTACGCCCCGCCCTGCCACTCATCGCAGTACTGTTGT
AATTCATTAAGCATTCTGCCGACATGGAAGCCATCACAGACGGCATGATGAACCT
GAATCGCCAGCGGCATCAGCACCTTGTCGCCTTGCGTATAATATTTGCCCATGGT
GAAAACGGGGGCGAAGAAGTTGTCCATATTGGCCACGTTTAAATCAAAACTGGTG
AAACTCACCCAGGGATTGGCTGAGACGAAAAACATATTCTCAATAAACCCTTTAG
GGAAATAGGCCAGGTTTTCACCGTAACACGCCACATCTTGCGAATATATGTGTAG
AAACTGCCGGAAATCGTCGTGGTATTCACTCCAGAGCGATGAAAACGTTTCAGTT
TGCTCATGGAAAACGGTGTAACAAGGGTGAACACTATCCCATATCACCAGCTCAC
CGTCTTTCATTGCCATACGGAATTCCGGATGAGCATTCATCAGGCGGGCAAGAAT
GTGAATAAAGGCCGGATAAAACTTGTGCTTATTTTTCTTTACGGTCTTTAAAAAGG
CCGTAATATCCAGCTGAACGGTCTGGTTATAGGTACATTGAGCAACTGACTGAAA
TGCCTCAAAATGTTCTTTACGATGCCATTGGGATATATCAACGGTGGTATATCCAG
TGATTTTTTTCTCCATTTTAGCTTCCTTAGC

pEVOL_PylRS2

TCCTGAAAATCTCGATAACTCAAAAAATACGCCCGGTAGTGATCTTATTTCATTAT
GGTGAAAGTTGGAACCTCTTACGTGCCGATCAACGTCTCATTTTCGCCAAAAGTT
GGCCCAGGGCTTCCCGGTATCAACAGGGACACCAGGATTTATTTATTCTGCGAAG
TGATCTTCCGTCACAGGTATTTATTCGGCGCAAAGTGCGTCGGGTGATGCTGCCA
ACTTACTGATTTAGTGTATGATGGTGTTTTTGAGGTGCTCCAGTGGCTTCTGTTTC
TATCAGCTGTCCCTCCTGTTCAGCTACTGACGGGGTGGTGCGTAACGGCAAAAGC
ACCGCCGGACATCAGCGCTAGCGGAGTGTATACTGGCTTACTATGTTGGCACTGA
TGAGGGTGTCAGTGAAGTGCTTCATGTGGCAGGAGAAAAAAGGCTGCACCGGTG
CGTCAGCAGAATATGTGATACAGGATATATTCCGCTTCCTCGCTCACTGACTCGCT
ACGCTCGGTCGTTCGACTGCGGCGAGCGGAAATGGCTTACGAACGGGGCGGAGA
TTTCCTGGAAGATGCCAGGAAGATACTTAACAGGGAAGTGAGAGGGCCGCGGCA
AAGCCGTTTTTCCATAGGCTCCGCCCCCCTGACAAGCATCACGAAATCTGACGCT
CAAATCAGTGGTGGCGAAACCCGACAGGACTATAAAGATACCAGGCGTTTCCCCC
TGGCGGCTCCCTCGTGCGCTCTCCTGTTCCTGCCTTTCGGTTTACCGGTGTCATTC
CGCTGTTATGGCCGCGTTTGTCTCATTCCACGCCTGACACTCAGTTCCGGGTAGG
CAGTTCGCTCCAAGCTGGACTGTATGCACGAACCCCCCGTTCAGTCCGACCGCTG

**CGCCTTATCCGGTAACTATCGTCTTGAGTCCAACCCGGAAAGACATGCAAAAGCA
CCACTGGCAGCAGCCACTGGTAATTGATTTAGAGGAGTTAGTCTTGAAGTCATGC
GCCGGTTAAGGCTAAACTGAAAGGACAAGTTTTGGTGACTGCGCTCCTCCAAGCC
AGTTACCTCGGTTCAAAGAGTTGGTAGCTCAGAGAACCTTCGAAAAACCGCCCTG
CAAGGCGGTTTTTTCGTTTTCAGAGCAAGAGATTACGCGCAGACCAAAACGATCT
CAAGAAGATCATCTTATTAATCAGATAAAATATTTCTAGATTTCAGTGCAATTTAT
CTCTTCAAATGTAGCACCTGAAGTCAGCCCCATACGATATAAGTTGTAATTCTCAT
GTTTGACAGCTTATCATCGATAAGCTTGGTACCCAATTATGACAACTTGACGGCTA
CATCATTCACTTTTTCTTCACAACCGGCACGGAACTCGCTCGGGCTGGCCCCGGT
GCATTTTTTAAATACCCGCGAGAAATAGAGTTGATCGTCAAAACCAACATTGCGA
CCGACGGTGGCGATAGGCATCCGGGTGGTGCTCAAAAGCAGCTTCGCCTGGCTG
ATACGTTGGTCCTCGCGCCAGCTTAAGACGCTAATCCCTAACTGCTGGCGGAAAA
GATGTGACAGACGCGACGGCGACAAGCAAACATGCTGTGCGACGCTGGCGATAT
CAAAATTGCTGTCTGCCAGGTGATCGCTGATGTACTGACAAGCCTCGCGTACCCG
ATTATCCATCGGTGGATGGAGCGACTCGTTAATCGCTTCCATGCGCCGCAGTAAC
AATTGCTCAAGCAGATTTATCGCCAGCAGCTCCGAATAGCGCCCTTCCCCTTGCC
CGGCGTTAATGATTTGCCCAAACAGGTCGCTGAAATGCGGCTGGTGCGCTTCATC
CGGGCGAAAGAACCCCGTATTGGCAAATATTGACGGCCAGTTAAGCCATTCATGC
CAGTAGGCGCGCGGACGAAAGTAAACCCACTGGTGATACCATTCGCGAGCCTCCG
GATGACGACCGTAGTGATGAATCTCTCCTGGCGGGAACAGCAAAATATCACTCGG
TCGGCAAACAAATTCTCGTCCCTGATTTTTCACCACCCCCTGACCGCGAATGGTG
AGATTGAGAATATAACCTTTCATTCCCAGCGGTCGGTCGATAAAAAAATCGAGAT
AACCGTTGGCCTCAATCGGCGTTAAACCCGCCACCAGATGGGCATTAAACGAGTA
TCCCGGCAGCAGGGGATCATTTTGCGCTTCAGCCATACTTTTCATACTCCCGCCAT
TCAGAGAAGAAACCAATTGTCCATATTGCATCAGACATTGCCGTCACTGCGTCTTT
TACTGGCTCTTCTCGCTAACCAAACCGGTAACCCCGCTTATTAAAAGCATTCTGTA
ACAAAGCGGGACCAAAGCCATGACAAAAACGCGTAACAAAAGTGTCTATAATCAC
GGCAGAAAAGTCCACATTGATTATTTGCACGGCGTCACACTTTGCTATGCCATAG
CATTTTTATCCATAAGATTAGCGGATCCTACCTGACGCTTTTTATCGCAACTCTCT
ACTGTTTCTCCATACCCGTTTTTTTGGGCTAACAGGAGGAATTAGATCTATGACGG
TCAAGTACACCGATGCTCAAATTCAACGTCTTCGCGAATATGGAAACGGAACCTA
CGAACAAAAGGTTTTCGAGGACTTAGCATCTCGTGACGCGGCTTTCAGTAAAGAG
ATGAGCGTGGCGTCAACTGACAATGAAAAAAAGATTAAAGGGATGATTGCAAATC
CATCACGTCATGGTTTAACGCAGTTAATGAATGATATTGCAGACGCATTAGTGGC
AGAGGGCTTTATTGAAGTCCGTACGCCGATTTTCATCTCCAAAGATGCTTTGGCA
CGTATGACTATCACCGAAGACAAGCCCCTGTTTAAGCAAGTTTTCTGGATCGACG
AAAAGCGTGCACTTCGCCCTATGTTGGCACCAAACCTGGCCTCCGTAGCGCGCGA
CTTACGCGATCACACCGACGGACCCGTGAAGATTTTCGAAATGGGATCATGTTTT
CGCAAGGAATCACATTCAGGGATGCATCTGGAGGAATTCACCATGTTGAACTTAG
TTGATATGGGACCGCGCGGCGATGCCACAGAAGTATTAAAAAACTACATCAGTGT
CGTAATGAAGGCTGCTGGTTTGCCTGATTATGATTTAGTACAAGAAGAAAGTGAT
GTATACAAAGAAACAATTGATGTGGAAATCAACGGGCAAGAAGTATGCAGTGCCG
CAGTGGGGCCGATTCCGCTGGATGCGGCCCATGACGTGCATGAGCCTTGGTCTG
GTGCTGGCTTCGGGTTGGAGCGTCTTTTAACGATTCGTGAGAAATACTCAACGGT
CAAGAAAGGCGGCGCTTCCATCAGTTACTTGAACGGCGCCAAGATTAATTGAGAC
CATCATCATCATCATCATTGAGTTTAAACGGTCTCCAGCTTGGCTGTTTTGGCGGA
TGAGAGAAGATTTTCAGCCTGATACAGATTAAATCAGAACGCAGAAGCGGTCTGA
TAAAACAGAATTTGCCTGGCGGCAGTAGCGCGGTGGTCCCACCTGACCCCATGCC
GAACTCAGAAGTGAAACGCCGTAGCGCCGATGGTAGTGTGGGGTCTCCCCATGC
GAGAGTAGGGAACTGCCAGGCATCAAATAAAACGAAAGGCTCAGTCGAAAGACT
GGGCCTTGTTTGTGAGCTCCCGGTCATCAATCATCCCCATAATCCTTGTTAGATTA
TCAATTTTAAAAAACTAACAGTTGTCAGCCTGTCCCGCTTTAATATCATACGCCGT
TATACGTTGTTTACGCTTTGAGGAATCCCATATGACGGTCAAGTACACCGATGCTC
AAATTCAACGTCTTCGCGAATATGGAAACGGAACCTACGAACAAAAGGTTTTCGA
GGACTTAGCATCTCGTGACGCGGCTTTCAGTAAAGAGATGAGCGTGGCGTCAACT**

GACAATGAAAAAAGATTAAAGGGATGATTGCAAATCCATCACGTCATGGTTTAA
CGCAGTTAATGAATGATATTGCAGACGCATTAGTGGCAGAGGGCTTTATTGAAGT
CCGTACGCCGATTTTCATCTCCAAAGATGCTTTGGCACGTATGACTATCACCGAA
GACAAGCCCCTGTTTAAGCAAGTTTTCTGGATCGACGAAAAGCGTGCACTTCGCC
CTATGTTGGCACCAAACCTGGCCTCCGTAGCGCGCGACTTACGCGATCACACCGA
CGGACCCGTGAAGATTTTCGAAATGGGATCATGTTTTCGCAAGGAATCACATTCA
GGGATGCATCTGGAGGAATTCACCATGTTGAACTTAGTTGATATGGGACCGCGCG
GCGATGCCACAGAAGTATTAAAAAACTACATCAGTGTCGTAATGAAGGCTGCTGG
TTTGCCTGATTATGATTTAGTACAAGAAGAAAGTGATGTATACAAAGAAACAATTG
ATGTGGAAATCAACGGGCAAGAAGTATGCAGTGCCGCAGTGGGGCCGATTCCGC
TGGATGCGGCCCATGACGTGCATGAGCCTTGGTCTGGTGCTGGCTTCGGGTTGGA
GCGTCTTTTAACGATTCGTGAGAAATACTCAACGGTCAAGAAAGGCGGCGCTTCC
ATCAGTTACTTGAACGGCGCCAAGATTAATCTGCAGTTTCAAACGCTAAATTGCCT
GATGCGCTACGCTTATCAGGCCTACATGATCTCTGCAATATATTGAGTTTGCGTGC
TTTTGTAGGCCGGATAAGGCGTTCACGCCGCATCCGGCAAGAAACAGCAAACAAT
CCAAAACGCCGCGTTCAGCGGCGTTTTTTCTGCTTTTCTTCGCGAATTAATTCCGC
TTCGCAACATGTGAGCACCGGTTTATTGACTACCGGAAGCAGTGTGACCGTGTGC
TTCTCAAATGCCTGAGGCCAGTTTGCTCAGGCTCTCCCCGTGGAGGTAATAATTG
ACGATATGATCAGTGCACGGCTAACTAAGCGGCCTGCTGACTTTCTCGCCGATCA
AAAGGCATTTTGCTATTAAGGGATTGACGAGGGCGTATCTGCGCAGTAAGATGCG
CCCCGCATTGGAAACCTGATCATGTAGATCGAATGGACTCTAAATCCGTTCAGCC
GGGTTAGATTCCCGGGGTTTCCGCCAAATTCGAAAAGCCTGCTCAACGAGCAGGC
TTTTTTGCATGCTCGAGCAGCTCAGGGTCGAATTTGCTTTCGAATTTCTGCCATTC
ATCCGCTTATTATCACTTATTCAGGCGTAGCACCAGGCGTTTAAGGGCACCAATA
ACTGCCTTAAAAAAATTACGCCCCGCCCTGCCACTCATCGCAGTACTGTTGTAATT
CATTAAGCATTCTGCCGACATGGAAGCCATCACAGACGGCATGATGAACCTGAAT
CGCCAGCGGCATCAGCACCTTGTCGCCTTGCGTATAATATTTGCCCATGGTGAAA
ACGGGGGCGAAGAAGTTGTCCATATTGGCCACGTTTAAATCAAAACTGGTGAAAC
TCACCCAGGGATTGGCTGAGACGAAAAACATATTCTCAATAAACCCTTTAGGGAA
ATAGGCCAGGTTTTCACCGTAACACGCCACATCTTGCGAATATATGTGTAGAAAC
TGCCGGAAATCGTCGTGGTATTCACTCCAGAGCGATGAAAACGTTTCAGTTTGCT
CATGGAAAACGGTGTAACAAGGGTGAACACTATCCCATATCACCAGCTCACCGTC
TTTCATTGCCATACGGAATTCCGGATGAGCATTCATCAGGCGGGCAAGAATGTGA
ATAAAGGCCGGATAAAACTTGTGCTTATTTTTCTTTACGGTCTTTAAAAAGGCCGT
AATATCCAGCTGAACGGTCTGGTTATAGGTACATTGAGCAACTGACTGAAATGCC
TCAAAATGTTCTTTACGATGCCATTGGGATATATCAACGGTGGTATATCCAGTGAT
TTTTTTCTCCATTTTAGCTTCCTTAGC

pEVOL_PylRS3

TCCTGAAAATCTCGATAACTCAAAAAATACGCCCGGTAGTGATCTTATTTCATTAT
GGTGAAAGTTGGAACCTCTTACGTGCCGATCAACGTCTCATTTTCGCCAAAAGTT
GGCCCAGGGCTTCCCGGTATCAACAGGGACACCAGGATTTATTTATTCTGCGAAG
TGATCTTCCGTCACAGGTATTTATTCGGCGCAAAGTGCGTCGGGTGATGCTGCCA
ACTTACTGATTTAGTGTATGATGGTGTTTTTGAGGTGCTCCAGTGGCTTCTGTTTC
TATCAGCTGTCCCTCCTGTTCAGCTACTGACGGGGTGGTGCGTAACGGCAAAAGC
ACCGCCGGACATCAGCGCTAGCGGAGTGTATACTGGCTTACTATGTTGGCACTGA
TGAGGGTGTCAGTGAAGTGCTTCATGTGGCAGGAGAAAAAAGGCTGCACCGGTG
CGTCAGCAGAATATGTGATACAGGATATATTCCGCTTCCTCGCTCACTGACTCGCT
ACGCTCGGTCGTTCGACTGCGGCGAGCGGAAATGGCTTACGAACGGGGCGGAGA
TTTCCTGGAAGATGCCAGGAAGATACTTAACAGGGAAGTGAGAGGGCCGCGGCA
AAGCCGTTTTTCCATAGGCTCCGCCCCCCTGACAAGCATCACGAAATCTGACGCT
CAAATCAGTGGTGGCGAAACCCGACAGGACTATAAAGATACCAGGCGTTTCCCCC
TGGCGGCTCCCTCGTGCGCTCTCCTGTTCCTGCCTTTCGGTTTACCGGTGTCATTC
CGCTGTTATGGCCGCGTTTGTCTCATTCCACGCCTGACACTCAGTTCCGGGTAGG

CAGTTCGCTCCAAGCTGGACTGTATGCACGAACCCCCCGTTCAGTCCGACCGCTG
CGCCTTATCCGGTAACTATCGTCTTGAGTCCAACCCGGAAAGACATGCAAAAGCA
CCACTGGCAGCAGCCACTGGTAATTGATTTAGAGGAGTTAGTCTTGAAGTCATGC
GCCGGTTAAGGCTAAACTGAAAGGACAAGTTTTGGTGACTGCGCTCCTCCAAGCC
AGTTACCTCGGTTCAAAGAGTTGGTAGCTCAGAGAACCTTCGAAAAACCGCCCTG
CAAGGCGGTTTTTTCGTTTTCAGAGCAAGAGATTACGCGCAGACCAAAACGATCT
CAAGAAGATCATCTTATTAATCAGATAAAATATTTCTAGATTTCAGTGCAATTTAT
CTCTTCAAATGTAGCACCTGAAGTCAGCCCCATACGATATAAGTTGTAATTCTCAT
GTTTGACAGCTTATCATCGATAAGCTTGGTACCCAATTATGACAACTTGACGGCTA
CATCATTCACTTTTTCTTCACAACCGGCACGGAACTCGCTCGGGCTGGCCCCGGT
GCATTTTTTAAATACCCGCGAGAAATAGAGTTGATCGTCAAAACCAACATTGCGA
CCGACGGTGGCGATAGGCATCCGGGTGGTGCTCAAAAGCAGCTTCGCCTGGCTG
ATACGTTGGTCCTCGCGCCAGCTTAAGACGCTAATCCCTAACTGCTGGCGGAAAA
GATGTGACAGACGCGACGGCGACAAGCAAACATGCTGTGCGACGCTGGCGATAT
CAAAATTGCTGTCTGCCAGGTGATCGCTGATGTACTGACAAGCCTCGCGTACCCG
ATTATCCATCGGTGGATGGAGCGACTCGTTAATCGCTTCCATGCGCCGCAGTAAC
AATTGCTCAAGCAGATTTATCGCCAGCAGCTCCGAATAGCGCCCTTCCCCTTGCC
CGGCGTTAATGATTTGCCCAAACAGGTCGCTGAAATGCGGCTGGTGCGCTTCATC
CGGGCGAAAGAACCCCGTATTGGCAAATATTGACGGCCAGTTAAGCCATTCATGC
CAGTAGGCGCGCGGACGAAAGTAAACCCACTGGTGATACCATTCGCGAGCCTCCG
GATGACGACCGTAGTGATGAATCTCTCCTGGCGGGAACAGCAAAATATCACTCGG
TCGGCAAACAAATTCTCGTCCCTGATTTTTCACCACCCCCTGACCGCGAATGGTG
AGATTGAGAATATAACCTTTCATTCCCAGCGGTCGGTCGATAAAAAAATCGAGAT
AACCGTTGGCCTCAATCGGCGTTAAACCCGCCACCAGATGGGCATTAAACGAGTA
TCCCGGCAGCAGGGGATCATTTTGCGCTTCAGCCATACTTTTCATACTCCCGCCAT
TCAGAGAAGAAACCAATTGTCCATATTGCATCAGACATTGCCGTCACTGCGTCTTT
TACTGGCTCTTCTCGCTAACCAAACCGGTAACCCCGCTTATTAAAAGCATTCTGTA
ACAAAGCGGGACCAAAGCCATGACAAAAACGCGTAACAAAAGTGTCTATAATCAC
GGCAGAAAAGTCCACATTGATTATTTGCACGGCGTCACACTTTGCTATGCCATAG
CATTTTTATCCATAAGATTAGCGGATCCTACCTGACGCTTTTTATCGCAACTCTCT
ACTGTTTCTCCATACCCGTTTTTTTGGGCTAACAGGAGGAATTAGATCATGACGGT
CAAGTACACCGATGCTCAAATTCAACGTCTTCGCGAATATGGAAACGGAACCTAC
GAACAAAAGGTTTTCGAGGACTTAGCATCTCGTGACGCGGCTTTCAGTAAAGAGA
TGAGCGTGGCGTCAACTGACAATGAAAAAAAGATTAAAGGGATGATTGCAAATCC
ATCACGTCATGGTTTAACGCAGTTAATGAATGATATTGCAGACGCATTAGTGGCA
GAGGGCTTTATTGAAGTCCGTACGCCGATTTTCATCTCCAAAGATGCTTTGGCAC
GTATGACTATCACCGAAGACAAGCCCCTGTTTAAGCAAGTTTTCTGGATCGACGA
AAAGCGTGCACTTCGCCCTATGTTGGCACCAAACCTGGCCTCCGTACTGCGCGAC
TTACGCGATCACACCGACGGACCCGTGAAGATTTTCGAAATGGGATCATGTTTTC
GCAAGGAATCACATTCAGGGATGCATCTGGAGGAATTCACCATGTTGAACTTAGT
TGATATGGGACCGCGCGGCGATGCCACAGAAGTATTAAAAAACTACATCAGTGTC
GTAATGAAGGCTGCTGGTTTGCCTGATTATGATTTAGTACAAGAAGAAAGTGATG
TATACAAAGAAACAATTGATGTGGAAATCAACGGGCAAGAAGTATGCAGTGCCGC
AGTGGGGCCGATTCCGCTGGATGCGGCCCATGACGTGCATGAGCCTTGGTCTGGT
GCTGGCTTCGGGTTGGAGCGTCTTTTAACGATTCGTGAGAAATACTCAACGGTCA
AGAAAGGCGGCGCTTCCATCAGTTACTTGAACGGCGCCAAGATTAATGTCGACCA
TCATCATCATCATCATTGAGTTTAAACGGTCTCCAGCTTGGCTGTTTTGGCGGATG
AGAGAAGATTTTCAGCCTGATACAGATTAAATCAGAACGCAGAAGCGGTCTGATA
AAACAGAATTTGCCTGGCGGCAGTAGCGCGGTGGTCCCACCTGACCCCATGCCGA
ACTCAGAAGTGAAACGCCGTAGCGCCGATGGTAGTGTGGGGTCTCCCCATGCGA
GAGTAGGGAACTGCCAGGCATCAAATAAAACGAAAGGCTCAGTCGAAAGACTGG
GCCTTGTTTGTGAGCTCCCGGTCATCAATCATCCCCATAATCCTTGTTAGATTATC
AATTTTAAAAAACTAACAGTTGTCAGCCTGTCCCGCTTTAATATCATACGCCGTTA
TACGTTGTTTACGCTTTGAGGAATCCCATATGACGGTCAAGTACACCGATGCTCA
AATTCAACGTCTTCGCGAATATGGAAACGGAACCTACGAACAAAAGGTTTTCGAG

**GACTTAGCATCTCGTGACGCGGCTTTCAGTAAAGAGATGAGCGTGGCGTCAACTG
ACAATGAAAAAAAGATTAAAGGGATGATTGCAAATCCATCACGTCATGGTTTAAC
GCAGTTAATGAATGATATTGCAGACGCATTAGTGGCAGAGGGCTTTATTGAAGTC
CGTACGCCGATTTTCATCTCCAAAGATGCTTTGGCACGTATGACTATCACCGAAG
ACAAGCCCCTGTTTAAGCAAGTTTTCTGGATCGACGAAAAGCGTGCACTTCGCCC
TATGTTGGCACCAAACCTGGCCTCCGTACTGCGCGACTTACGCGATCACACCGAC
GGACCCGTGAAGATTTTCGAAATGGGATCATGTTTTCGCAAGGAATCACATTCAG
GGATGCATCTGGAGGAATTCACCATGTTGAACTTAGTTGATATGGGACCGCGCGG
CGATGCCACAGAAGTATTAAAAAACTACATCAGTGTCGTAATGAAGGCTGCTGGT
TTGCCTGATTATGATTTAGTACAAGAAGAAAGTGATGTATACAAAGAAACAATTGA
TGTGGAAATCAACGGGCAAGAAGTATGCAGTGCCGCAGTGGGGCCGATTCCGCT
GGATGCGGCCCATGACGTGCATGAGCCTTGGTCTGGTGCTGGCTTCGGGTTGGA
GCGTCTTTTAACGATTCGTGAGAAATACTCAACGGTCAAGAAAGGCGGCGCTTCC
ATCAGTTACTTGAACGGCGCCAAGATTAATTGACAGTTTCAAACGCTAAATTGCCT
GATGCGCTACGCTTATCAGGCCTACATGATCTCTGCAATATATTGAGTTTGCGTGC
TTTTGTAGGCCGGATAAGGCGTTCACGCCGCATCCGGCAAGAAACAGCAAACAAT
CCAAAACGCCGCGTTCAGCGGCGTTTTTTCTGCTTTTCTTCGCGAATTAATTCCGC
TTCGCAACATGTGAGCACCGGTTTATTGACTACCGGAAGCAGTGTGACCGTGTGC
TTCTCAAATGCCTGAGGCCAGTTTGCTCAGGCTCTCCCCGTGGAGGTAATAATTG
ACGATATGATCAGTGCACGGCTAACTAAGCGGCCTGCTGACTTTCTCGCCGATCA
AAAGGCATTTTGCTATTAAGGGATTGACGAGGGCGTATCTGCGCAGTAAGATGCG
CCCCGCATTGGGGGACGGTCCGGCGACCAGCGGGTCTCTAAAAC<u>CTA</u>GCCAGCG
GGGTTCGACGCCCCGGTCTCTCGCCAAATTCGAAAAGCCTGCTCAACGAGCAGGC
TTTTTTGCATGCTCGAGCAGCTCAGGGTCGAATTTGCTTTCGAATTTCTGCCATTC
ATCCGCTTATTATCACTTATTCAGGCGTAGCACCAGGCGTTTAAGGGCACCAATA
ACTGCCTTAAAAAAATTACGCCCCGCCCTGCCACTCATCGCAGTACTGTTGTAATT
CATTAAGCATTCTGCCGACATGGAAGCCATCACAGACGGCATGATGAACCTGAAT
CGCCAGCGGCATCAGCACCTTGTCGCCTTGCGTATAATATTTGCCCATGGTGAAA
ACGGGGGCGAAGAAGTTGTCCATATTGGCCACGTTTAAATCAAAACTGGTGAAAC
TCACCCAGGGATTGGCTGAGACGAAAAACATATTCTCAATAAACCCTTTAGGGAA
ATAGGCCAGGTTTTCACCGTAACACGCCACATCTTGCGAATATATGTGTAGAAAC
TGCCGGAAATCGTCGTGGTATTCACTCCAGAGCGATGAAAACGTTTCAGTTTGCT
CATGGAAAACGGTGTAACAAGGGTGAACACTATCCCATATCACCAGCTCACCGTC
TTTCATTGCCATACGGAATTCCGGATGAGCATTCATCAGGCGGGCAAGAATGTGA
ATAAAGGCCGGATAAAACTTGTGCTTATTTTTCTTTACGGTCTTTAAAAAGGCCGT
AATATCCAGCTGAACGGTCTGGTTATAGGTACATTGAGCAACTGACTGAAATGCC
TCAAAATGTTCTTTACGATGCCATTGGGATATATCAACGGTGGTATATCCAGTGAT
TTTTTTCTCCATTTTAGCTTCCTTAGC**

pFI1_PylRS1_UAG_SCFP (pFI1) and pFI1_PylRS1_AAA_SCFP (pFI1+)
TCCTGAAAATCTCGATAACTCAAAAAATACGCCCGGTAGTGATCTTATTTCATTAT<br>GGTGAAAGTTGGAACCTCTTACGTGCCGATCAACGTCTCATTTTCGCCAAAAGTT<br>GGCCCAGGGCTTCCCGGTATCAACAGGGACACCAGGATTTATTTATTCTGCGAAG<br>TGATCTTCCGTCACAGGTATTTATTCGGCGCAAAGTGCGTCGGGTGATGCTGCCA<br>ACTTACTGATTTAGTGTATGATGGTGTTTTTGAGGTGCTCCAGTGGCTTCTGTTTC<br>TATCAGCTGTCCCTCCTGTTCAGCTACTGACGGGGTGGTGCGTAACGGCAAAAGC<br>ACCGCCGGACATCAGCGCTAGCGGAGTGTATACTGGCTTACTATGTTGGCACTGA<br>TGAGGGTGTCAGTGAAGTGCTTCATGTGGCAGGAGAAAAAAGGCTGCACCGGTG<br>CGTCAGCAGAATATGTGATACAGGATATATTCCGCTTCCTCGCTCACTGACTCGCT<br>ACGCTCGGTCGTTCGACTGCGGCGAGCGGAAATGGCTTACGAACGGGGCGGAGA<br>TTTCCTGGAAGATGCCAGGAAGATACTTAACAGGGAAGTGAGAGGGCCGCGGCA<br>AAGCCGTTTTTCCATAGGCTCCGCCCCCCTGACAAGCATCACGAAATCTGACGCT<br>CAAATCAGTGGTGGCGAAACCCGACAGGACTATAAAGATACCAGGCGTTTCCCCC<br>TGGCGGCTCCCTCGTGCGCTCTCCTGTTCCTGCCTTTCGGTTTACCGGTGTCATTC

**CGCTGTTATGGCCGCGTTTGTCTCATTCCACGCCTGACACTCAGTTCCGGGTAGG
CAGTTCGCTCCAAGCTGGACTGTATGCACGAACCCCCCGTTCAGTCCGACCGCTG
CGCCTTATCCGGTAACTATCGTCTTGAGTCCAACCCGGAAAGACATGCAAAAGCA
CCACTGGCAGCAGCCACTGGTAATTGATTTAGAGGAGTTAGTCTTGAAGTCATGC
GCCGGTTAAGGCTAAACTGAAAGGACAAGTTTTGGTGACTGCGCTCCTCCAAGCC
AGTTACCTCGGTTCAAAGAGTTGGTAGCTCAGAGAACCTTCGAAAAACCGCCCTG
CAAGGCGGTTTTTTCGTTTTCAGAGCAAGAGATTACGCGCAGACCAAAACGATCT
CAAGAAGATCATCTTATTAATCAGATAAAATATTTCTAGATTTCAGTGCAATTTAT
CTCTTCAAATGTAGCACCTGAAGTCAGCCCCATACGATATAAGTTGTAATTCTCAT
GTTTGACAGCTTATCATCGATAAGCTTGGTACCCAATTATGACAACTTGACGGCTA
CATCATTCACTTTTTCTTCACAACCGGCACGGAACTCGCTCGGGCTGGCCCCGGT
GCATTTTTTAAATACCCGCGAGAAATAGAGTTGATCGTCAAAACCAACATTGCGA
CCGACGGTGGCGATAGGCATCCGGGTGGTGCTCAAAAGCAGCTTCGCCTGGCTG
ATACGTTGGTCCTCGCGCCAGCTTAAGACGCTAATCCCTAACTGCTGGCGGAAAA
GATGTGACAGACGCGACGGCGACAAGCAAACATGCTGTGCGACGCTGGCGATAT
CAAAATTGCTGTCTGCCAGGTGATCGCTGATGTACTGACAAGCCTCGCGTACCCG
ATTATCCATCGGTGGATGGAGCGACTCGTTAATCGCTTCCATGCGCCGCAGTAAC
AATTGCTCAAGCAGATTTATCGCCAGCAGCTCCGAATAGCGCCCTTCCCCTTGCC
CGGCGTTAATGATTTGCCCAAACAGGTCGCTGAAATGCGGCTGGTGCGCTTCATC
CGGGCGAAAGAACCCCGTATTGGCAAATATTGACGGCCAGTTAAGCCATTCATGC
CAGTAGGCGCGCGGACGAAAGTAAACCCACTGGTGATACCATTCGCGAGCCTCCG
GATGACGACCGTAGTGATGAATCTCTCCTGGCGGGAACAGCAAAATATCACTCGG
TCGGCAAACAAATTCTCGTCCCTGATTTTTCACCACCCCCTGACCGCGAATGGTG
AGATTGAGAATATAACCTTTCATTCCCAGCGGTCGGTCGATAAAAAAATCGAGAT
AACCGTTGGCCTCAATCGGCGTTAAACCCGCCACCAGATGGGCATTAAACGAGTA
TCCCGGCAGCAGGGGATCATTTTGCGCTTCAGCCATACTTTTCATACTCCCGCCAT
TCAGAGAAGAAACCAATTGTCCATATTGCATCAGACATTGCCGTCACTGCGTCTTT
TACTGGCTCTTCTCGCTAACCAAACCGGTAACCCCGCTTATTAAAAGCATTCTGTA
ACAAAGCGGGACCAAAGCCATGACAAAAACGCGTAACAAAAGTGTCTATAATCAC
GGCAGAAAAGTCCACATTGATTATTTGCACGGCGTCACACTTTGCTATGCCATAG
CATTTTTATCCATAAGATTAGCGGATCCTACCTGACGCTTTTTATCGCAACTCTCT
ACTGTTTCTCCATACCCGTTTTTTTGGGCTAACAGGAGGAATTAGATCATGACGGT
CAAGTACACCGATGCTCAAATTCAACGTCTTCGCGAATATGGAAACGGAACCTAC
GAACAAAAGGTTTTCGAGGACTTAGCATCTCGTGACGCGGCTTTCAGTAAAGAGA
TGAGCGTGGCGTCAACTGACAATGAAAAAAAGATTAAAGGGATGATTGCAAATCC
ATCACGTCATGGTTTAACGCAGTTAATGAATGATATTGCAGACGCATTAGTGGCA
GAGGGCTTTATTGAAGTCCGTACGCCGATTTTCATCTCCAAAGATGCTTTGGCAC
GTATGACTATCACCGAAGACAAGCCCCTGTTTAAGCAAGTTTTCTGGATCGACGA
AAAGCGTGCACTTCGCCCTATGTTGGCACCAAACCTGGCCTCCGTACTGCGCGAC
TTACGCGATCACACCGACGGACCCGTGAAGATTTTCGAAATGGGATCATGTTTTC
GCAAGGAATCACATTCAGGGATGCATCTGGAGGAATTCACCATGTTGAACTTAGT
TGATATGGGACCGCGCGGCGATGCCACAGAAGTATTAAAAAACTACATCAGTGTC
GTAATGAAGGCTGCTGGTTTGCCTGATTATGATTTAGTACAAGAAGAAAGTGATG
TATACAAAGAAACAATTGATGTGGAAATCAACGGGCAAGAAGTATGCAGTGCCGC
AGTGGGGCCGATTCCGCTGGATGCGGCCCATGACGTGCATGAGCCTTGGTCTGGT
GCTGGCTTCGGGTTGGAGCGTCTTTTAACGATTCGTGAGAAATACTCAACGGTCA
AGAAAGGCGGCGCTTCCATCAGTTACTTGAACGGCGCCAAGATTAATGTCGACCA
TCATCATCATCATCATTGAGTTTAAACGGTCTCCAGCTTGGCTGTTTTGGCGGATG
AGAGAAGATTTTCAGCCTGATACAGATTAAATCAGAACGCAGAAGCGGTCTGATA
AAACAGAATTTGCCTGGCGGCAGTAGCGCGGTGGTCCCACCTGACCCCATGCCGA
ACTCAGAAGTGAAACGCCGTAGCGCCGATGGTAGTGTGGGGTCTCCCCATGCGA
GAGTAGGGAACTGCCAGGCATCAAATAAAACGAAAGGCTCAGTCGAAAGACTGG
GCCTTGTTTGTGAGCTCCCGGTCATCAATCATCCCCATAATCCTTGTTAGATTATC
AATTTTAAAAAACTAACAGTTGTCAGCCTGTCCCGCTTTAATATCATACGCCGTTA
TACGTTGTTTACGCTTTGAGGAATCCCATATGACGGTCAAGTACACCGATGCTCA**

AATTCAACGTCTTCGCGAATATGGAAACGGAACCTACGAACAAAAGGTTTTCGAG
GACTTAGCATCTCGTGACGCGGCTTTCAGTAAAGAGATGAGCGTGGCGTCAACTG
ACAATGAAAAAAAGATTAAAGGGATGATTGCAAATCCATCACGTCATGGTTTAAC
GCAGTTAATGAATGATATTGCAGACGCATTAGTGGCAGAGGGCTTTATTGAAGTC
CGTACGCCGATTTTCATCTCCAAAGATGCTTTGGCACGTATGACTATCACCGAAG
ACAAGCCCCTGTTTAAGCAAGTTTTCTGGATCGACGAAAAGCGTGCACTTCGCCC
TATGTTGGCACCAAACCTGGCCTCCGTACTGCGCGACTTACGCGATCACACCGAC
GGACCCGTGAAGATTTTCGAAATGGGATCATGTTTTCGCAAGGAATCACATTCAG
GGATGCATCTGGAGGAATTCACCATGTTGAACTTAGTTGATATGGGACCGCGCGG
CGATGCCACAGAAGTATTAAAAAACTACATCAGTGTCGTAATGAAGGCTGCTGGT
TTGCCTGATTATGATTTAGTACAAGAAGAAAGTGATGTATACAAAGAAACAATTGA
TGTGGAAATCAACGGGCAAGAAGTATGCAGTGCCGCAGTGGGGCCGATTCCGCT
GGATGCGGCCCATGACGTGCATGAGCCTTGGTCTGGTGCTGGCTTCGGGTTGGA
GCGTCTTTTAACGATTCGTGAGAAATACTCAACGGTCAAGAAAGGCGGCGCTTCC
ATCAGTTACTTGAACGGCGCCAAGATTAATTGACAGTTTCAAACGCTAAATTGCCT
GATGCGCTACGCTTATCAGGCCTACATGATCTCTGCAATATATTGAGTTTGCGTGC
TTTTGTAGGCCGGATAAGGCGTTCACGCCGCATCCGGCAAGAAACAGCAAACAAT
CCAAAACGCCGCGTTCAGCGGCGTTTTTTCTGCTTTTCTTCGCGAATTAATTCCGC
TTCGCAACATGTGAGCACCGGTTTATTGACTACCGGAAGCAGTGTGACCGTGTGC
TTCTCAAATGCCTGAGGCCAGTTTGCTCAGGCTCTCCCCGTGGAGGTAATAATTG
ACGATATGATCAGTGCACGGCTAACTAAGCGGCCTGCTGACTTTCTCGCCGATCA
AAAGGCATTTTGCTATTAAGGGATTGACGAGGGCGTATCTGCGCAGTAAGATGCG
CCCCGCATTGGGGGACGGTCCGGCGACCAGCGGGTCTCTAAAACCTAGCCAGCG
GGGTTCGACGCCCCGGTCTCTCGCCAAATTCGAAAAGCCTGCTCAACGAGCAGGC
TTTTTTGCATGCTCGAGCAGCTCAGTCATAAAAAATTTATTTGCTTTGTGAGCGGA
TAACAATTATAATAGATTCAATTGTGAGCGGATAACAATTTCACACAGAATTCATT
AAAGAGGAGAAATTAACTATGGGCCATCATTAG/AAAAAGAATGGCGGTGCGAGC
AGTAAAGGAGAAGAACTTTTCACTGGAGTTGTCCCAATTCTTGTTGAATTAGATG
GTGATGTTAATGGGCACTAGTTTTCTGTCAGTGGAGAGGGTGAAGGTGATGCAAC
ATACGGAAAACTTACCCTTAAATTTATTTGCACTACTGGAAAACTACCTGTTCCAT
GGCCAACACTTGTCACTACTTTGACCTGGGGTGTTCAATGCTTTGCGAGATACCC
AGATCATATGAAACAGCATGACTTTTTCAAGAGTGCCATGCCCGAAGGTTATGTA
CAGGAAAGAACTATATTTTTCAAAGATGACGGGAACTACAAGACACGTGCTGAAG
TCAAGTTTGAAGGTGATACCCTTGTTAATAGAATCGAGTTAAAAGGTATTGATTTT
AAAGAAGATGGAAACATTCTTGGACACAAATTGGAATACAACTATATCTCAGACA
ATGTATACATCACGGCAGACAAACAAAAGAATGGAATCAAAGCGAACTTCAAAAT
TAGACACAACATTGAAGATGGAGGTGTTCAACTAGCAGACCATTATCAACAAAAT
ACTCCAATTGGCGATGGCCCTGTCCTTTTACCAGACAACCATTACCTGTCCACCCA
ATCTAAGCTCTCGAAAGATCCCAACGAAAAGAGAGACCACATGGTCCTTCTTGAG
TTTGTAACAGCTGCTGGGATTACACATGGCATGGATGAACTATACAAATAAGACT
CCTGTTGATAGATCCAGTAATGACCTCAGAACTCCATCTGGATTTGTTCAGAACGC
TCGGTTGCCGCCGGGCGTTTTTTATTGGTGAGAATCCAAGCTAGCTTGGCGAGAT
TTTCAGGAGCTAAGGAAGCTAAATTTTTTTAAGGCAGTTATTGGTGCCCTTAAACG
CCTGGGGTAATGACTCTCTAGCTTGAGGCATCAAATAAAACGAAAGGCTCAGTCG
AAAGACTGGGCCTTTCGTTTTATCTGTTGTTTGTCGGTGAACGCTCTCCTGAGTAG
GACAAATCCGCCCTCTAGACTGGGTTGAAGGCTCTCAAGGGCATCGGTCGAGATC
CCGGTGCCTAATGAGTGAGCTAACTTACATTAATTGCGTTGCGCTCACTGCCCGC
TTTCCAGTCGGGAAACCTGTCGTGCCAGCTGCATTAATGAATCGGCCAACGCGCG
GGGAGAGGCGGTTTGCGTATTGGGCGCCAGGGTGGTTTTTCTTTTCACCAGTGAG
ACGGGCAACAGCTGATTGCCCTTCACCGCCTGGCCCTGAGAGAGTTGCAGCAAGC
GGTCCACGCTGGTTTGCCCCAGCAGGCGAAAATCCTGTTTGATGGTGGTTAACGG
CGGGATATAACATGAGCTGTCTTCGGTATCGTCGTATCCCACTACCGAGATATCC
GCACCAACGCGCAGCCCGGACTCGGTAATGGCGCGCATTGCGCCCAGCGCCATC
TGATCGTTGGCAACCAGCATCGCAGTGGGAACGATGCCCTCATTCAGCATTTGCA
TGGTTTGTTGAAAACCGGACATGGCACTCCAGTCGCCTTCCCGTTCCGCTATCGG

CTGAATTTGATTGCGAGTGAGATATTTATGCCAGCCAGCCAGACGCAGACGCGCC
GAGACAGAACTTAATGGGCCCGCTAACAGCGCGATTTGCTGGTGACCCAATGCGA
CCAGATGCTCCACGCCCAGTCGCGTACCGTCTTCATGGGAGAAAATAATACTGTT
GATGGGTGTCTGGTCAGAGACATCAAGAAATAACGCCGGAACATTAGTGCAGGCA
GCTTCCACAGCAATGGCATCCTGGTCATCCAGCGGATAGTTAATGATCAGCCCAC
TGACGCGTTGCGCGAGAAGATTGTGCACCGCCGCTTTACAGGCTTCGACGCCGCT
TCGTTCTACCATCGACACCACCACGCTGGCACCCAGTTGATCGGCGCGAGATTTA
ATCGCCGCGACAATTTGCGACGGCGCGTGCAGGGCCAGACTGGAGGTGGCAACG
CCAATCAGCAACGACTGTTTGCCCGCCAGTTGTTGTGCCACGCGGTTGGGAATGT
AATTCAGCTCCGCCATCGCCGCTTCCACTTTTTCCCGCGTTTTCGCAGAAACGTGG
CTGGCCTGGTTCACCACGCGGGAAACGGTCTGATAAGAGACACCGGCATACTCTG
CGACATCGTATAACGTTACTGGTTTCACATTCACCACCCTGAATTGACTCTCTTCC
GGGCGCTATCATGCCATACCGCGAAAGGTTTTGCACCATTCGATGGTGTCCAATA
ACTGCCTTAAAAAATTACGCCCCGCCCTGCCACTCATCGCAGTACTGTTGTAATT
CATTAAGCATTCTGCCGACATGGAAGCCATCACAGACGGCATGATGAACCTGAAT
CGCCAGCGGCATCAGCACCTTGTCGCCTTGCGTATAATATTTGCCCATGGTGAAA
ACGGGGGCGAAGAAGTTGTCCATATTGGCCACGTTTAAATCAAAACTGGTGAAAC
TCACCCAGGGATTGGCTGAGACGAAAAACATATTCTCAATAAACCCTTTAGGGAA
ATAGGCCAGGTTTTCACCGTAACACGCCACATCTTGCGAATATATGTGTAGAAAC
TGCCGGAAATCGTCGTGGTATTCACTCCAGAGCGATGAAAACGTTTCAGTTTGCT
CATGGAAAACGGTGTAACAAGGGTGAACACTATCCCATATCACCAGCTCACCGTC
TTTCATTGCCATACGGAATTCCGGATGAGCATTCATCAGGCGGGCAAGAATGTGA
ATAAAGGCCGGATAAAACTTGTGCTTATTTTTCTTTACGGTCTTTAAAAAGGCCGT
AATATCCAGCTGAACGGTCTGGTTATAGGTACATTGAGCAACTGACTGAAATGCC
TCAAAATGTTCTTTACGATGCCATTGGGATATATCAACGGTGGTATATCCAGTGAT
TTTTTTCTCCATTTTAGCTTCCTTAGC

pFI1_PylRS2_UAG_SCFP (pFI1) and pFI1_PylRS2_AAA_SCFP (pFI1+)

TCCTGAAAATCTCGATAACTCAAAAAATACGCCCGGTAGTGATCTTATTTCATTAT
GGTGAAAGTTGGAACCTCTTACGTGCCGATCAACGTCTCATTTTCGCCAAAAGTT
GGCCCAGGGCTTCCCGGTATCAACAGGGACACCAGGATTTATTTATTCTGCGAAG
TGATCTTCCGTCACAGGTATTTATTCGGCGCAAAGTGCGTCGGGTGATGCTGCCA
ACTTACTGATTTAGTGTATGATGGTGTTTTTGAGGTGCTCCAGTGGCTTCTGTTTC
TATCAGCTGTCCCTCCTGTTCAGCTACTGACGGGGTGGTGCGTAACGGCAAAAGC
ACCGCCGGACATCAGCGCTAGCGGAGTGTATACTGGCTTACTATGTTGGCACTGA
TGAGGGTGTCAGTGAAGTGCTTCATGTGGCAGGAGAAAAAAGGCTGCACCGGTG
CGTCAGCAGAATATGTGATACAGGATATATTCCGCTTCCTCGCTCACTGACTCGCT
ACGCTCGGTCGTTCGACTGCGGCGAGCGGAAATGGCTTACGAACGGGGCGGAGA
TTTCCTGGAAGATGCCAGGAAGATACTTAACAGGGAAGTGAGAGGGCCGCGGCA
AAGCCGTTTTTCCATAGGCTCCGCCCCCCTGACAAGCATCACGAAATCTGACGCT
CAAATCAGTGGTGGCGAAACCCGACAGGACTATAAAGATACCAGGCGTTTCCCCC
TGGCGGCTCCCTCGTGCGCTCTCCTGTTCCTGCCTTTCGGTTTACCGGTGTCATTC
CGCTGTTATGGCCGCGTTTGTCTCATTCCACGCCTGACACTCAGTTCCGGGTAGG
CAGTTCGCTCCAAGCTGGACTGTATGCACGAACCCCCCGTTCAGTCCGACCGCTG
CGCCTTATCCGGTAACTATCGTCTTGAGTCCAACCCGGAAAGACATGCAAAAGCA
CCACTGGCAGCAGCCACTGGTAATTGATTTAGAGGAGTTAGTCTTGAAGTCATGC
GCCGGTTAAGGCTAAACTGAAAGGACAAGTTTTGGTGACTGCGCTCCTCCAAGCC
AGTTACCTCGGTTCAAAGAGTTGGTAGCTCAGAGAACCTTCGAAAAACCGCCCTG
CAAGGCGGTTTTTTCGTTTTCAGAGCAAGAGATTACGCGCAGACCAAAACGATCT
CAAGAAGATCATCTTATTAATCAGATAAAATATTTCTAGATTTCAGTGCAATTTAT
CTCTTCAAATGTAGCACCTGAAGTCAGCCCCATACGATATAAGTTGTAATTCTCAT
GTTTGACAGCTTATCATCGATAAGCTTGGTACCCAATTATGACAACTTGACGGCTA
CATCATTCACTTTTTCTTCACAACCGGCACGGAACTCGCTCGGGCTGGCCCCGGT
GCATTTTTAAATACCCGCGAGAAATAGAGTTGATCGTCAAAACCAACATTGCGA

**CCGACGGTGGCGATAGGCATCCGGGTGGTGCTCAAAAGCAGCTTCGCCTGGCTG
ATACGTTGGTCCTCGCGCCAGCTTAAGACGCTAATCCCTAACTGCTGGCGGAAAA
GATGTGACAGACGCGACGGCGACAAGCAAACATGCTGTGCGACGCTGGCGATAT
CAAAATTGCTGTCTGCCAGGTGATCGCTGATGTACTGACAAGCCTCGCGTACCCG
ATTATCCATCGGTGGATGGAGCGACTCGTTAATCGCTTCCATGCGCCGCAGTAAC
AATTGCTCAAGCAGATTTATCGCCAGCAGCTCCGAATAGCGCCCTTCCCCTTGCC
CGGCGTTAATGATTTGCCCAAACAGGTCGCTGAAATGCGGCTGGTGCGCTTCATC
CGGGCGAAAGAACCCCGTATTGGCAAATATTGACGGCCAGTTAAGCCATTCATGC
CAGTAGGCGCGCGGACGAAAGTAAACCCACTGGTGATACCATTCGCGAGCCTCCG
GATGACGACCGTAGTGATGAATCTCTCCTGGCGGGAACAGCAAAATATCACTCGG
TCGGCAAACAAATTCTCGTCCCTGATTTTTCACCACCCCCTGACCGCGAATGGTG
AGATTGAGAATATAACCTTTCATTCCCAGCGGTCGGTCGATAAAAAAATCGAGAT
AACCGTTGGCCTCAATCGGCGTTAAACCCGCCACCAGATGGGCATTAAACGAGTA
TCCCGGCAGCAGGGGATCATTTTGCGCTTCAGCCATACTTTTCATACTCCCGCCAT
TCAGAGAAGAAACCAATTGTCCATATTGCATCAGACATTGCCGTCACTGCGTCTTT
TACTGGCTCTTCTCGCTAACCAAACCGGTAACCCCGCTTATTAAAAGCATTCTGTA
ACAAAGCGGGACCAAAGCCATGACAAAAACGCGTAACAAAAGTGTCTATAATCAC
GGCAGAAAAGTCCACATTGATTATTTGCACGGCGTCACACTTTGCTATGCCATAG
CATTTTTATCCATAAGATTAGCGGATCCTACCTGACGCTTTTTATCGCAACTCTCT
ACTGTTTCTCCATACCCGTTTTTTTGGGCTAACAGGAGGAATTAGATCATGACGGT
CAAGTACACCGATGCTCAAATTCAACGTCTTCGCGAATATGGAAACGGAACCTAC
GAACAAAAGGTTTTCGAGGACTTAGCATCTCGTGACGCGGCTTTCAGTAAAGAGA
TGAGCGTGGCGTCAACTGACAATGAAAAAAAGATTAAAGGGATGATTGCAAATCC
ATCACGTCATGGTTTAACGCAGTTAATGAATGATATTGCAGACGCATTAGTGGCA
GAGGGCTTTATTGAAGTCCGTACGCCGATTTTCATCTCCAAAGATGCTTTGGCAC
GTATGACTATCACCGAAGACAAGCCCCTGTTTAAGCAAGTTTTCTGGATCGACGA
AAAGCGTGCACTTCGCCCTATGTTGGCACCAAACCTGGCCTCCGTACTGCGCGAC
TTACGCGATCACACCGACGGACCCGTGAAGATTTTCGAAATGGGATCATGTTTTC
GCAAGGAATCACATTCAGGGATGCATCTGGAGGAATTCACCATGTTGAACTTAGT
TGATATGGGACCGCGCGGCGATGCCACAGAAGTATTAAAAAACTACATCAGTGTC
GTAATGAAGGCTGCTGGTTTGCCTGATTATGATTTAGTACAAGAAGAAAGTGATG
TATACAAAGAAACAATTGATGTGGAAATCAACGGGCAAGAAGTATGCAGTGCCGC
AGTGGGGCCGATTCCGCTGGATGCGGCCCATGACGTGCATGAGCCTTGGTCTGGT
GCTGGCTTCGGGTTGGAGCGTCTTTTAACGATTCGTGAGAAATACTCAACGGTCA
AGAAAGGCGGCGCTTCCATCAGTTACTTGAACGGCGCCAAGATTAATGTCGACCA
TCATCATCATCATCATTGAGTTTAAACGGTCTCCAGCTTGGCTGTTTTGGCGGATG
AGAGAAGATTTTCAGCCTGATACAGATTAAATCAGAACGCAGAAGCGGTCTGATA
AAACAGAATTTGCCTGGCGGCAGTAGCGCGGTGGTCCCACCTGACCCCATGCCGA
ACTCAGAAGTGAAACGCCGTAGCGCCGATGGTAGTGTGGGGTCTCCCCATGCGA
GAGTAGGGAACTGCCAGGCATCAAATAAAACGAAAGGCTCAGTCGAAAGACTGG
GCCTTGTTTGTGAGCTCCCGGTCATCAATCATCCCCATAATCCTTGTTAGATTATC
AATTTTAAAAAACTAACAGTTGTCAGCCTGTCCCGCTTTAATATCATACGCCGTTA
TACGTTGTTTACGCTTTGAGGAATCCCATATGACGGTCAAGTACACCGATGCTCA
AATTCAACGTCTTCGCGAATATGGAAACGGAACCTACGAACAAAAGGTTTTCGAG
GACTTAGCATCTCGTGACGCGGCTTTCAGTAAAGAGATGAGCGTGGCGTCAACTG
ACAATGAAAAAAAGATTAAAGGGATGATTGCAAATCCATCACGTCATGGTTTAAC
GCAGTTAATGAATGATATTGCAGACGCATTAGTGGCAGAGGGCTTTATTGAAGTC
CGTACGCCGATTTTCATCTCCAAAGATGCTTTGGCACGTATGACTATCACCGAAG
ACAAGCCCCTGTTTAAGCAAGTTTTCTGGATCGACGAAAAGCGTGCACTTCGCCC
TATGTTGGCACCAAACCTGGCCTCCGTACTGCGCGACTTACGCGATCACACCGAC
GGACCCGTGAAGATTTTCGAAATGGGATCATGTTTTCGCAAGGAATCACATTCAG
GGATGCATCTGGAGGAATTCACCATGTTGAACTTAGTTGATATGGGACCGCGCGG
CGATGCCACAGAAGTATTAAAAAACTACATCAGTGTCGTAATGAAGGCTGCTGGT
TTGCCTGATTATGATTTAGTACAAGAAGAAAGTGATGTATACAAAGAAACAATTGA
TGTGGAAATCAACGGGCAAGAAGTATGCAGTGCCGCAGTGGGGCCGATTCCGCT**

**GGATGCGGCCCATGACGTGCATGAGCCTTGGTCTGGTGCTGGCTTCGGGTTGGA
GCGTCTTTTAACGATTCGTGAGAAATACTCAACGGTCAAGAAAGGCGGCGCTTCC
ATCAGTTACTTGAACGGCGCCAAGATTAATTGACAGTTTCAAACGCTAAATTGCCT
GATGCGCTACGCTTATCAGGCCTACATGATCTCTGCAATATATTGAGTTTGCGTGC
TTTTGTAGGCCGGATAAGGCGTTCACGCCGCATCCGGCAAGAAACAGCAAACAAT
CCAAAACGCCGCGTTCAGCGGCGTTTTTTCTGCTTTTCTTCGCGAATTAATTCCGC
TTCGCAACATGTGAGCACCGGTTTATTGACTACCGGAAGCAGTGTGACCGTGTGC
TTCTCAAATGCCTGAGGCCAGTTTGCTCAGGCTCTCCCCGTGGAGGTAATAATTG
ACGATATGATCAGTGCACGGCTAACTAAGCGGCCTGCTGACTTTCTCGCCGATCA
AAAGGCATTTTGCTATTAAGGGATTGACGAGGGCGTATCTGCGCAGTAAGATGCG
CCCCGCATTGGGGGACGGTCCGGCGACCAGCGGGTCTCTAAAACCTAGCCAGCG
GGGTTCGACGCCCCGGTCTCTCGCCAAATTCGAAAAGCCTGCTCAACGAGCAGGC
TTTTTTGCATGCTCGAGCAGCTCAGTCATAAAAAATTTATTTGCTTTGTGAGCGGA
TAACAATTATAATAGATTCAATTGTGAGCGGATAACAATTTCACACAGAATTCATT
AAAGAGGAGAAATTAACTATGGGCCATCATTAG/AAAAAGAATGGCGGTGCGAGC
AGTAAAGGAGAAGAACTTTTCACTGGAGTTGTCCCAATTCTTGTTGAATTAGATG
GTGATGTTAATGGGCACTAGTTTTCTGTCAGTGGAGAGGGTGAAGGTGATGCAAC
ATACGGAAAACTTACCCTTAAATTTATTTGCACTACTGGAAAACTACCTGTTCCAT
GGCCAACACTTGTCACTACTTTGACCTGGGGTGTTCAATGCTTTGCGAGATACCC
AGATCATATGAAACAGCATGACTTTTTCAAGAGTGCCATGCCCGAAGGTTATGTA
CAGGAAAGAACTATATTTTTCAAAGATGACGGGAACTACAAGACACGTGCTGAAG
TCAAGTTTGAAGGTGATACCCTTGTTAATAGAATCGAGTTAAAAGGTATTGATTTT
AAAGAAGATGGAAACATTCTTGGACACAAATTGGAATACAACTATATCTCAGACA
ATGTATACATCACGGCAGACAAACAAAAGAATGGAATCAAAGCGAACTTCAAAAT
TAGACACAACATTGAAGATGGAGGTGTTCAACTAGCAGACCATTATCAACAAAAT
ACTCCAATTGGCGATGGCCCTGTCCTTTTACCAGACAACCATTACCTGTCCACCCA
ATCTAAGCTCTCGAAAGATCCCAACGAAAAGAGAGACCACATGGTCCTTCTTGAG
TTTGTAACAGCTGCTGGGATTACACATGGCATGGATGAACTATACAAATAAGACT
CCTGTTGATAGATCCAGTAATGACCTCAGAACTCCATCTGGATTTGTTCAGAACGC
TCGGTTGCCGCCGGGCGTTTTTTATTGGTGAGAATCCAAGCTAGCTTGGCGAGAT
TTTCAGGAGCTAAGGAAGCTAAATTTTTTTAAGGCAGTTATTGGTGCCCTTAAACG
CCTGGGGTAATGACTCTCTAGCTTGAGGCATCAAATAAAACGAAAGGCTCAGTCG
AAAGACTGGGCCTTTCGTTTTATCTGTTGTTTGTCGGTGAACGCTCTCCTGAGTAG
GACAAATCCGCCCTCTAGACTGGGTTGAAGGCTCTCAAGGGCATCGGTCGAGATC
CCGGTGCCTAATGAGTGAGCTAACTTACATTAATTGCGTTGCGCTCACTGCCCGC
TTTCCAGTCGGGAAACCTGTCGTGCCAGCTGCATTAATGAATCGGCCAACGCGCG
GGGAGAGGCGGTTTGCGTATTGGGCGCCAGGGTGGTTTTTCTTTTCACCAGTGAG
ACGGGCAACAGCTGATTGCCCTTCACCGCCTGGCCCTGAGAGAGTTGCAGCAAGC
GGTCCACGCTGGTTTGCCCCAGCAGGCGAAAATCCTGTTTGATGGTGGTTAACGG
CGGGATATAACATGAGCTGTCTTCGGTATCGTCGTATCCCACTACCGAGATATCC
GCACCAACGCGCAGCCCGGACTCGGTAATGGCGCGCATTGCGCCCAGCGCCATC
TGATCGTTGGCAACCAGCATCGCAGTGGGAACGATGCCCTCATTCAGCATTTGCA
TGGTTTGTTGAAAACCGGACATGGCACTCCAGTCGCCTTCCCGTTCCGCTATCGG
CTGAATTTGATTGCGAGTGAGATATTTATGCCAGCCAGCCAGACGCAGACGCGCC
GAGACAGAACTTAATGGGCCCGCTAACAGCGCGATTTGCTGGTGACCCAATGCGA
CCAGATGCTCCACGCCCAGTCGCGTACCGTCTTCATGGGAGAAAATAATACTGTT
GATGGGTGTCTGGTCAGAGACATCAAGAAATAACGCCGGAACATTAGTGCAGGCA
GCTTCCACAGCAATGGCATCCTGGTCATCCAGCGGATAGTTAATGATCAGCCCAC
TGACGCGTTGCGCGAGAAGATTGTGCACCGCCGCTTTACAGGCTTCGACGCCGCT
TCGTTCTACCATCGACACCACCACGCTGGCACCCAGTTGATCGGCGCGAGATTTA
ATCGCCGCGACAATTTGCGACGGCGCGTGCAGGGCCAGACTGGAGGTGGCAACG
CCAATCAGCAACGACTGTTTGCCCGCCAGTTGTTGTGCCACGCGGTTGGGAATGT
AATTCAGCTCCGCCATCGCCGCTTCCACTTTTTCCCGCGTTTTCGCAGAAACGTGG
CTGGCCTGGTTCACCACGCGGGAAACGGTCTGATAAGAGACACCGGCATACTCTG
CGACATCGTATAACGTTACTGGTTTCACATTCACCACCCTGAATTGACTCTCTTCC**

GGGCGCTATCATGCCATACCGCGAAAGGTTTTGCACCATTCGATGGTGTCCAATA<br>ACTGCCTTAAAAAAATTACGCCCCGCCCTGCCACTCATCGCAGTACTGTTGTAATT<br>CATTAAGCATTCTGCCGACATGGAAGCCATCACAGACGGCATGATGAACCTGAAT<br>CGCCAGCGGCATCAGCACCTTGTCGCCTTGCGTATAATATTTGCCCATGGTGAAA<br>ACGGGGGCGAAGAAGTTGTCCATATTGGCCACGTTTAAATCAAAACTGGTGAAAC<br>TCACCCAGGGATTGGCTGAGACGAAAAACATATTCTCAATAAACCCTTTAGGGAA<br>ATAGGCCAGGTTTTCACCGTAACACGCCACATCTTGCGAATATATGTGTAGAAAC<br>TGCCGGAAATCGTCGTGGTATTCACTCCAGAGCGATGAAAACGTTTCAGTTTGCT<br>CATGGAAAACGGTGTAACAAGGGTGAACACTATCCCATATCACCAGCTCACCGTC<br>TTTCATTGCCATACGGAATTCCGGATGAGCATTCATCAGGCGGGCAAGAATGTGA<br>ATAAAGGCCGGATAAAACTTGTGCTTATTTTTCTTTACGGTCTTTAAAAAGGCCGT<br>AATATCCAGCTGAACGGTCTGGTTATAGGTACATTGAGCAACTGACTGAAATGCC<br>TCAAAATGTTCTTTACGATGCCATTGGGATATATCAACGGTGGTATATCCAGTGAT<br>TTTTTTCTCCATTTTAGCTTCCTTAGC

pFI1_PylRS3_UAG_SCFP (pFI1) and pFI1_PylRS3_AAA_SCFP (pFI1+)
TCCTGAAAATCTCGATAACTCAAAAAATACGCCCGGTAGTGATCTTATTTCATTAT<br>GGTGAAAGTTGGAACCTCTTACGTGCCGATCAACGTCTCATTTTCGCCAAAAGTT<br>GGCCCAGGGCTTCCCGGTATCAACAGGGACACCAGGATTTATTTATTCTGCGAAG<br>TGATCTTCCGTCACAGGTATTTATTCGGCGCAAAGTGCGTCGGGTGATGCTGCCA<br>ACTTACTGATTTAGTGTATGATGGTGTTTTTGAGGTGCTCCAGTGGCTTCTGTTTC<br>TATCAGCTGTCCCTCCTGTTCAGCTACTGACGGGGTGGTGCGTAACGGCAAAAGC<br>ACCGCCGGACATCAGCGCTAGCGGAGTGTATACTGGCTTACTATGTTGGCACTGA<br>TGAGGGTGTCAGTGAAGTGCTTCATGTGGCAGGAGAAAAAAGGCTGCACCGGTG<br>CGTCAGCAGAATATGTGATACAGGATATATTCCGCTTCCTCGCTCACTGACTCGCT<br>ACGCTCGGTCGTTCGACTGCGGCGAGCGGAAATGGCTTACGAACGGGGCGGAGA<br>TTTCCTGGAAGATGCCAGGAAGATACTTAACAGGGAAGTGAGAGGGCCGCGGCA<br>AAGCCGTTTTTCCATAGGCTCCGCCCCCCTGACAAGCATCACGAAATCTGACGCT<br>CAAATCAGTGGTGGCGAAACCCGACAGGACTATAAAGATACCAGGCGTTTCCCCC<br>TGGCGGCTCCCTCGTGCGCTCTCCTGTTCCTGCCTTTCGGTTTACCGGTGTCATTC<br>CGCTGTTATGGCCGCGTTTGTCTCATTCCACGCCTGACACTCAGTTCCGGGTAGG<br>CAGTTCGCTCCAAGCTGGACTGTATGCACGAACCCCCCGTTCAGTCCGACCGCTG<br>CGCCTTATCCGGTAACTATCGTCTTGAGTCCAACCCGGAAAGACATGCAAAAGCA<br>CCACTGGCAGCAGCCACTGGTAATTGATTTAGAGGAGTTAGTCTTGAAGTCATGC<br>GCCGGTTAAGGCTAAACTGAAAGGACAAGTTTTGGTGACTGCGCTCCTCCAAGCC<br>AGTTACCTCGGTTCAAAGAGTTGGTAGCTCAGAGAACCTTCGAAAAACCGCCCTG<br>CAAGGCGGTTTTTTCGTTTTCAGAGCAAGAGATTACGCGCAGACCAAAACGATCT<br>CAAGAAGATCATCTTATTAATCAGATAAAATATTTCTAGATTTCAGTGCAATTTAT<br>CTCTTCAAATGTAGCACCTGAAGTCAGCCCCATACGATATAAGTTGTAATTCTCAT<br>GTTTGACAGCTTATCATCGATAAGCTTGGTACCCAATTATGACAACTTGACGGCTA<br>CATCATTCACTTTTTCTTCACAACCGGCACGGAACTCGCTCGGGCTGGCCCCGGT<br>GCATTTTTTAAATACCCGCGAGAAATAGAGTTGATCGTCAAAACCAACATTGCGA<br>CCGACGGTGGCGATAGGCATCCGGGTGGTGCTCAAAAGCAGCTTCGCCTGGCTG<br>ATACGTTGGTCCTCGCGCCAGCTTAAGACGCTAATCCCTAACTGCTGGCGGAAAA<br>GATGTGACAGACGCGACGGCGACAAGCAAACATGCTGTGCGACGCTGGCGATAT<br>CAAAATTGCTGTCTGCCAGGTGATCGCTGATGTACTGACAAGCCTCGCGTACCCG<br>ATTATCCATCGGTGGATGGAGCGACTCGTTAATCGCTTCCATGCGCCGCAGTAAC<br>AATTGCTCAAGCAGATTTATCGCCAGCAGCTCCGAATAGCGCCCTTCCCCTTGCC<br>CGGCGTTAATGATTTGCCCAAACAGGTCGCTGAAATGCGGCTGGTGCGCTTCATC<br>CGGGCGAAAGAACCCCGTATTGGCAAATATTGACGGCCAGTTAAGCCATTCATGC<br>CAGTAGGCGCGCGGACGAAAGTAAACCCACTGGTGATACCATTCGCGAGCCTCCG<br>GATGACGACCGTAGTGATGAATCTCTCCTGGCGGGAACAGCAAAATATCACTCGG<br>TCGGCAAACAAATTCTCGTCCCTGATTTTTCACCACCCCCTGACCGCGAATGGTG<br>AGATTGAGAATATAACCTTTCATTCCCAGCGGTCGGTCGATAAAAAAATCGAGAT

AACCGTTGGCCTCAATCGGCGTTAAACCCGCCACCAGATGGGCATTAAACGAGTA
TCCCGGCAGCAGGGGATCATTTTGCGCTTCAGCCATACTTTTCATACTCCCGCCAT
TCAGAGAAGAAACCAATTGTCCATATTGCATCAGACATTGCCGTCACTGCGTCTTT
TACTGGCTCTTCTCGCTAACCAAACCGGTAACCCCGCTTATTAAAAGCATTCTGTA
ACAAAGCGGGACCAAAGCCATGACAAAAACGCGTAACAAAAGTGTCTATAATCAC
GGCAGAAAAGTCCACATTGATTATTTGCACGGCGTCACACTTTGCTATGCCATAG
CATTTTTATCCATAAGATTAGCGGATCCTACCTGACGCTTTTTATCGCAACTCTCT
ACTGTTTCTCCATACCCGTTTTTTTGGGCTAACAGGAGGAATTAGATCATGACGGT
CAAGTACACCGATGCTCAAATTCAACGTCTTCGCGAATATGGAAACGGAACCTAC
GAACAAAAGGTTTTCGAGGACTTAGCATCTCGTGACGCGGCTTTCAGTAAAGAGA
TGAGCGTGGCGTCAACTGACAATGAAAAAAAGATTAAAGGGATGATTGCAAATCC
ATCACGTCATGGTTTAACGCAGTTAATGAATGATATTGCAGACGCATTAGTGGCA
GAGGGCTTTATTGAAGTCCGTACGCCGATTTTCATCTCCAAAGATGCTTTGGCAC
GTATGACTATCACCGAAGACAAGCCCCTGTTTAAGCAAGTTTTCTGGATCGACGA
AAAGCGTGCACTTCGCCCTATGTTGGCACCAAACCTGGCCTCCGTACTGCGCGAC
TTACGCGATCACACCGACGGACCCGTGAAGATTTTCGAAATGGGATCATGTTTTC
GCAAGGAATCACATTCAGGGATGCATCTGGAGGAATTCACCATGTTGAACTTAGT
TGATATGGGACCGCGCGGCGATGCCACAGAAGTATTAAAAAACTACATCAGTGTC
GTAATGAAGGCTGCTGGTTTGCCTGATTATGATTTAGTACAAGAAGAAAGTGATG
TATACAAAGAAACAATTGATGTGGAAATCAACGGGCAAGAAGTATGCAGTGCCGC
AGTGGGGCCGATTCCGCTGGATGCGGCCCATGACGTGCATGAGCCTTGGTCTGGT
GCTGGCTTCGGGTTGGAGCGTCTTTTAACGATTCGTGAGAAATACTCAACGGTCA
AGAAAGGCGGCGCTTCCATCAGTTACTTGAACGGCGCCAAGATTAATGTCGACCA
TCATCATCATCATCATTGAGTTTAAACGGTCTCCAGCTTGGCTGTTTTGGCGGATG
AGAGAAGATTTTCAGCCTGATACAGATTAAATCAGAACGCAGAAGCGGTCTGATA
AAACAGAATTTGCCTGGCGGCAGTAGCGCGGTGGTCCCACCTGACCCCATGCCGA
ACTCAGAAGTGAAACGCCGTAGCGCCGATGGTAGTGTGGGGTCTCCCCATGCGA
GAGTAGGGAACTGCCAGGCATCAAATAAAACGAAAGGCTCAGTCGAAAGACTGG
GCCTTGTTTGTGAGCTCCCGGTCATCAATCATCCCCATAATCCTTGTTAGATTATC
AATTTTAAAAAACTAACAGTTGTCAGCCTGTCCCGCTTTAATATCATACGCCGTTA
TACGTTGTTTACGCTTTGAGGAATCCCATATGACGGTCAAGTACACCGATGCTCA
AATTCAACGTCTTCGCGAATATGGAAACGGAACCTACGAACAAAAGGTTTTCGAG
GACTTAGCATCTCGTGACGCGGCTTTCAGTAAAGAGATGAGCGTGGCGTCAACTG
ACAATGAAAAAAAGATTAAAGGGATGATTGCAAATCCATCACGTCATGGTTTAAC
GCAGTTAATGAATGATATTGCAGACGCATTAGTGGCAGAGGGCTTTATTGAAGTC
CGTACGCCGATTTTCATCTCCAAAGATGCTTTGGCACGTATGACTATCACCGAAG
ACAAGCCCCTGTTTAAGCAAGTTTTCTGGATCGACGAAAAGCGTGCACTTCGCCC
TATGTTGGCACCAAACCTGGCCTCCGTACTGCGCGACTTACGCGATCACACCGAC
GGACCCGTGAAGATTTTCGAAATGGGATCATGTTTTCGCAAGGAATCACATTCAG
GGATGCATCTGGAGGAATTCACCATGTTGAACTTAGTTGATATGGGACCGCGCGG
CGATGCCACAGAAGTATTAAAAAACTACATCAGTGTCGTAATGAAGGCTGCTGGT
TTGCCTGATTATGATTTAGTACAAGAAGAAAGTGATGTATACAAAGAAACAATTGA
TGTGGAAATCAACGGGCAAGAAGTATGCAGTGCCGCAGTGGGGCCGATTCCGCT
GGATGCGGCCCATGACGTGCATGAGCCTTGGTCTGGTGCTGGCTTCGGGTTGGA
GCGTCTTTTAACGATTCGTGAGAAATACTCAACGGTCAAGAAAGGCGGCGCTTCC
ATCAGTTACTTGAACGGCGCCAAGATTAATTGACAGTTTCAAACGCTAAATTGCCT
GATGCGCTACGCTTATCAGGCCTACATGATCTCTGCAATATATTGAGTTTGCGTGC
TTTTGTAGGCCGGATAAGGCGTTCACGCCGCATCCGGCAAGAAACAGCAAACAAT
CCAAAACGCCGCGTTCAGCGGCGTTTTTTCTGCTTTTCTTCGCGAATTAATTCCGC
TTCGCAACATGTGAGCACCGGTTTATTGACTACCGGAAGCAGTGTGACCGTGTGC
TTCTCAAATGCCTGAGGCCAGTTTGCTCAGGCTCTCCCCGTGGAGGTAATAATTG
ACGATATGATCAGTGCACGGCTAACTAAGCGGCCTGCTGACTTTCTCGCCGATCA
AAAGGCATTTTGCTATTAAGGGATTGACGAGGGCGTATCTGCGCAGTAAGATGCG
CCCCGCATTGGGGGACGGTCCGGCGACCAGCGGGTCTCTAAAACCTAGCCAGCG
GGGTTCGACGCCCCGGTCTCTCGCCAAATTCGAAAAGCCTGCTCAACGAGCAGGC

TTTTTTGCATGCTCGAGCAGCTCAGTCATAAAAAATTTATTTGCTTTGTGAGCGGA
TAACAATTATAATAGATTCAATTGTGAGCGGATAACAATTTCACACAGAATTCATT
AAAGAGGAGAAATTAACTATGGGCCATCATTAG/AAAAAGAATGGCGGTGCGAGC
AGTAAAGGAGAAGAACTTTTCACTGGAGTTGTCCCAATTCTTGTTGAATTAGATG
GTGATGTTAATGGGCACTAGTTTTCTGTCAGTGGAGAGGGTGAAGGTGATGCAAC
ATACGGAAAACTTACCCTTAAATTTATTTGCACTACTGGAAAACTACCTGTTCCAT
GGCCAACACTTGTCACTACTTTGACCTGGGGTGTTCAATGCTTTGCGAGATACCC
AGATCATATGAAACAGCATGACTTTTTCAAGAGTGCCATGCCCGAAGGTTATGTA
CAGGAAAGAACTATATTTTTCAAAGATGACGGGAACTACAAGACACGTGCTGAAG
TCAAGTTTGAAGGTGATACCCTTGTTAATAGAATCGAGTTAAAAGGTATTGATTTT
AAAGAAGATGGAAACATTCTTGGACACAAATTGGAATACAACTATATCTCAGACA
ATGTATACATCACGGCAGACAAACAAAAGAATGGAATCAAAGCGAACTTCAAAAT
TAGACACAACATTGAAGATGGAGGTGTTCAACTAGCAGACCATTATCAACAAAAT
ACTCCAATTGGCGATGGCCCTGTCCTTTTACCAGACAACCATTACCTGTCCACCCA
ATCTAAGCTCTCGAAAGATCCCAACGAAAAGAGAGACCACATGGTCCTTCTTGAG
TTTGTAACAGCTGCTGGGATTACACATGGCATGGATGAACTATACAAATAAGACT
CCTGTTGATAGATCCAGTAATGACCTCAGAACTCCATCTGGATTTGTTCAGAACGC
TCGGTTGCCGCCGGGCGTTTTTTATTGGTGAGAATCCAAGCTAGCTTGGCGAGAT
TTTCAGGAGCTAAGGAAGCTAAATTTTTTTAAGGCAGTTATTGGTGCCCTTAAACG
CCTGGGGTAATGACTCTCTAGCTTGAGGCATCAAATAAAACGAAAGGCTCAGTCG
AAAGACTGGGCCTTTCGTTTTATCTGTTGTTTGTCGGTGAACGCTCTCCTGAGTAG
GACAAATCCGCCCTCTAGACTGGGTTGAAGGCTCTCAAGGGCATCGGTCGAGATC
CCGGTGCCTAATGAGTGAGCTAACTTACATTAATTGCGTTGCGCTCACTGCCCGC
TTTCCAGTCGGGAAACCTGTCGTGCCAGCTGCATTAATGAATCGGCCAACGCGCG
GGGAGAGGCGGTTTGCGTATTGGGCGCCAGGGTGGTTTTTCTTTTCACCAGTGAG
ACGGGCAACAGCTGATTGCCCTTCACCGCCTGGCCCTGAGAGAGTTGCAGCAAGC
GGTCCACGCTGGTTTGCCCCAGCAGGCGAAAATCCTGTTTGATGGTGGTTAACGG
CGGGATATAACATGAGCTGTCTTCGGTATCGTCGTATCCCACTACCGAGATATCC
GCACCAACGCGCAGCCCGGACTCGGTAATGGCGCGCATTGCGCCCAGCGCCATC
TGATCGTTGGCAACCAGCATCGCAGTGGGAACGATGCCCTCATTCAGCATTTGCA
TGGTTTGTTGAAAACCGGACATGGCACTCCAGTCGCCTTCCCGTTCCGCTATCGG
CTGAATTTGATTGCGAGTGAGATATTTATGCCAGCCAGCCAGACGCAGACGCGCC
GAGACAGAACTTAATGGGCCCGCTAACAGCGCGATTTGCTGGTGACCCAATGCGA
CCAGATGCTCCACGCCCAGTCGCGTACCGTCTTCATGGGAGAAAATAATACTGTT
GATGGGTGTCTGGTCAGAGACATCAAGAAATAACGCCGGAACATTAGTGCAGGCA
GCTTCCACAGCAATGGCATCCTGGTCATCCAGCGGATAGTTAATGATCAGCCCAC
TGACGCGTTGCGCGAGAAGATTGTGCACCGCCGCTTTACAGGCTTCGACGCCGCT
TCGTTCTACCATCGACACCACCACGCTGGCACCCAGTTGATCGGCGCGAGATTTA
ATCGCCGCGACAATTTGCGACGGCGCGTGCAGGGCCAGACTGGAGGTGGCAACG
CCAATCAGCAACGACTGTTTGCCCGCCAGTTGTTGTGCCACGCGGTTGGGAATGT
AATTCAGCTCCGCCATCGCCGCTTCCACTTTTTCCCGCGTTTTCGCAGAAACGTGG
CTGGCCTGGTTCACCACGCGGGAAACGGTCTGATAAGAGACACCGGCATACTCTG
CGACATCGTATAACGTTACTGGTTTCACATTCACCACCCTGAATTGACTCTCTTCC
GGGCGCTATCATGCCATACCGCGAAAGGTTTTGCACCATTCGATGGTGTCCAATA
ACTGCCTTAAAAAAATTACGCCCCGCCCTGCCACTCATCGCAGTACTGTTGTAATT
CATTAAGCATTCTGCCGACATGGAAGCCATCACAGACGGCATGATGAACCTGAAT
CGCCAGCGGCATCAGCACCTTGTCGCCTTGCGTATAATATTTGCCCATGGTGAAA
ACGGGGGCGAAGAAGTTGTCCATATTGGCCACGTTTAAATCAAAACTGGTGAAAC
TCACCCAGGGATTGGCTGAGACGAAAAACATATTCTCAATAAACCCTTTAGGGAA
ATAGGCCAGGTTTTCACCGTAACACGCCACATCTTGCGAATATATGTGTAGAAAC
TGCCGGAAATCGTCGTGGTATTCACTCCAGAGCGATGAAAACGTTTCAGTTTGCT
CATGGAAAACGGTGTAACAAGGGTGAACACTATCCCATATCACCAGCTCACCGTC
TTTCATTGCCATACGGAATTCCGGATGAGCATTCATCAGGCGGGCAAGAATGTGA
ATAAAGGCCGGATAAAACTTGTGCTTATTTTTCTTTACGGTCTTTAAAAAGGCCGT
AATATCCAGCTGAACGGTCTGGTTATAGGTACATTGAGCAACTGACTGAAATGCC

TCAAAATGTTCTTTACGATGCCATTGGGATATATCAACGGTGGTATATCCAGTGAT
TTTTTTCTCCATTTTAGCTTCCTTAGC

pFI2 - tac promoter, lac operator

**TCCTGAAAATCTCGATAACTCAAAAAATACGCCCGGTAGTGATCTTATTTCATTAT
GGTGAAAGTTGGAACCTCTTACGTGCCGATCAACGTCTCATTTTCGCCAAAAGTT
GGCCCAGGGCTTCCCGGTATCAACAGGGACACCAGGATTTATTTATTCTGCGAAG
TGATCTTCCGTCACAGGTATTTATTCGGCGCAAAGTGCGTCGGGTGATGCTGCCA
ACTTACTGATTTAGTGTATGATGGTGTTTTTGAGGTGCTCCAGTGGCTTCTGTTTC
TATCAGCTGTCCCTCCTGTTCAGCTACTGACGGGGTGGTGCGTAACGGCAAAAGC
ACCGCCGGACATCAGCGCTAGCGGAGTGTATACTGGCTTACTATGTTGGCACTGA
TGAGGGTGTCAGTGAAGTGCTTCATGTGGCAGGAGAAAAAAGGCTGCACCGGTG
CGTCAGCAGAATATGTGATACAGGATATATTCCGCTTCCTCGCTCACTGACTCGCT
ACGCTCGGTCGTTCGACTGCGGCGAGCGGAAATGGCTTACGAACGGGGCGGAGA
TTTCCTGGAAGATGCCAGGAAGATACTTAACAGGGAAGTGAGAGGGCCGCGGCA
AAGCCGTTTTTCCATAGGCTCCGCCCCCCTGACAAGCATCACGAAATCTGACGCT
CAAATCAGTGGTGGCGAAACCCGACAGGACTATAAAGATACCAGGCGTTTCCCCC
TGGCGGCTCCCTCGTGCGCTCTCCTGTTCCTGCCTTTCGGTTTACCGGTGTCATTC
CGCTGTTATGGCCGCGTTTGTCTCATTCCACGCCTGACACTCAGTTCCGGGTAGG
CAGTTCGCTCCAAGCTGGACTGTATGCACGAACCCCCCGTTCAGTCCGACCGCTG
CGCCTTATCCGGTAACTATCGTCTTGAGTCCAACCCGGAAAGACATGCAAAAGCA
CCACTGGCAGCAGCCACTGGTAATTGATTTAGAGGAGTTAGTCTTGAAGTCATGC
GCCGGTTAAGGCTAAACTGAAAGGACAAGTTTTGGTGACTGCGCTCCTCCAAGCC
AGTTACCTCGGTTCAAAGAGTTGGTAGCTCAGAGAACCTTCGAAAAACCGCCCTG
CAAGGCGGTTTTTTCGTTTTCAGAGCAAGAGATTACGCGCAGACCAAAACGATCT
CAAGAAGATCATCTTATTAATCAGATAAAATATTTCTAGATTTCAGTGCAATTTAT
CTCTTCAAATGTAGCACCTGAAGTCAGCCCCATACGATATAAGTTGTAATTCTCAT
GTTTGACAGCTTATCATCGATAAGCTTGGTACCCAATTGACAATTAATCATCGGCT
CGTATAATGTGTGGAATTGTGAGCGGATAACAAACCCGTTTTTTTGGGCTAACAG
GAGGAATTATGACGGTCAAGTACACCGATGCTCAAATTCAACGTCTTCGCGAATA
TGGAAACGGAACCTACGAACAAAAGGTTTTCGAGGACTTAGCATCTCGTGACGCG
GCTTTCAGTAAAGAGATGAGCGTGGCGTCAACTGACAATGAAAAAAAGATTAAAG
GGATGATTGCAAATCCATCACGTCATGGTTTAACGCAGTTAATGAATGATATTGCA
GACGCATTAGTGGCAGAGGGCTTTATTGAAGTCCGTACGCCGATTTTCATCTCCA
AAGATGCTTTGGCACGTATGACTATCACCGAAGACAAGCCCCTGTTTAAGCAAGT
TTTCTGGATCGACGAAAAGCGTGCACTTCGCCCTATGTTGGCACCAAACCTGGCC
TCCGTACTGCGCGACTTACGCGATCACACCGACGGACCCGTGAAGATTTTCGAAA
TGGGATCATGTTTTCGCAAGGAATCACATTCAGGGATGCATCTGGAGGAATTCAC
CATGTTGAACTTAGTTGATATGGGACCGCGCGGCGATGCCACAGAAGTATTAAAA
AACTACATCAGTGTCGTAATGAAGGCTGCTGGTTTGCCTGATTATGATTTAGTACA
AGAAGAAAGTGATGTATACAAAGAAACAATTGATGTGGAAATCAACGGGCAAGAA
GTATGCAGTGCCGCAGTGGGGCCGATTCCGCTGGATGCGGCCCATGACGTGCAT
GAGCCTTGGTCTGGTGCTGGCTTCGGGTTGGAGCGTCTTTTAACGATTCGTGAGA
AATACTCAACGGTCAAGAAAGGCGGCGCTTCCATCAGTTACTTGAACGGCGCCAA
GATTAATTGAGCTTCCATCAGTTACTTGAACGGCGCCAAGATTAATGTCGACCATC
ATCATCATCATCATTGAGTTTAAACGGTCTCCAGCTTGGCTGTTTTGGCGGATGAG
AGAAGATTTTCAGCCTGATACAGATTAAATCAGAACGCAGAAGCGGTCTGATAAA
ACAGAATTTGCCTGGCGGCAGTAGCGCGGTGGTCCCACCTGACCCCATGCCGAAC
TCAGAAGTGAAACGCCGTAGCGCCGATGGTAGTGTGGGGTCTCCCCATGCGAGA
GTAGGGAACTGCCAGGCATCAAATAAAACGAAAGGCTCAGTCGAAAGACTGGGC
CTTGTTTGTGAGCTCCCGGTCATCAATCATCCCCATAATCCTTGTTAGATTATCAA
TTTTAAAAAACTAACAGTTGTCAGCCTGTCCCGCTTTAATATCATACGCCGTTATA**

CGTTGTTTACGCTTTGAGGAATCCCATATGACGGTCAAGTACACCGATGCTCAAA
TTCAACGTCTTCGCGAATATGGAAACGGAACCTACGAACAAAAGGTTTTCGAGGA
CTTAGCATCTCGTGACGCGGCTTTCAGTAAAGAGATGAGCGTGGCGTCAACTGAC
AATGAAAAAAAGATTAAAGGGATGATTGCAAATCCATCACGTCATGGTTTAACGC
AGTTAATGAATGATATTGCAGACGCATTAGTGGCAGAGGGCTTTATTGAAGTCCG
TACGCCGATTTTCATCTCCAAAGATGCTTTGGCACGTATGACTATCACCGAAGACA
AGCCCCTGTTTAAGCAAGTTTTCTGGATCGACGAAAAGCGTGCACTTCGCCCTAT
GTTGGCACCAAACCTGGCCTCCGTACTGCGCGACTTACGCGATCACACCGACGGA
CCCGTGAAGATTTTCGAAATGGGATCATGTTTTCGCAAGGAATCACATTCAGGGA
TGCATCTGGAGGAATTCACCATGTTGAACTTAGTTGATATGGGACCGCGCGGCGA
TGCCACAGAAGTATTAAAAAACTACATCAGTGTCGTAATGAAGGCTGCTGGTTTG
CCTGATTATGATTTAGTACAAGAAGAAAGTGATGTATACAAAGAAACAATTGATGT
GGAAATCAACGGGCAAGAAGTATGCAGTGCCGCAGTGGGGCCGATTCCGCTGGA
TGCGGCCCATGACGTGCATGAGCCTTGGTCTGGTGCTGGCTTCGGGTTGGAGCGT
CTTTTAACGATTCGTGAGAAATACTCAACGGTCAAGAAAGGCGGCGCTTCCATCA
GTTACTTGAACGGCGCCAAGATTAATTGACAGTTTCAAACGCTAAATTGCCTGAT
GCGCTACGCTTATCAGGCCTACATGATCTCTGCAATATATTGAGTTTGCGTGCTTT
TGTAGGCCGGATAAGGCGTTCACGCCGCATCCGGCAAGAAACAGCAAACAATCCA
AAACGCCGCGTTCAGCGGCGTTTTTTCTGCTTTTCTTCGCGAATTAATTCCGCTTC
GCAACATGTGAGCACCGGTTTATTGACTACCGGAAGCAGTGTGACCGTGTGCTTC
TCAAATGCCTGAGGCCAGTTTGCTCAGGCTCTCCCCGTGGAGGTAATAATTGACG
ATATGATCAGTGCACGGCTAACTAAGCGGCCTGCTGACTTTCTCGCCGATCAAAA
GGCATTTTGCTATTAAGGGATTGACGAGGGCGTATCTGCGCAGTAAGATGCGCCC
CGCATTGGGGGACGGTCCGGCGACCAGCGGGTCTCTAAAACCTAGCCAGCGGGG
TTCGACGCCCCGGTCTCTCGCCAAATTCGAAAAGCCTGCTCAACGAGCAGGCTTT
TTTGCATGCTCGAGCAGCTCAGTCATAAAAAATTTATTTGCTTTGTGAGCGGATAA
CAATTATAATAGATTCAATTGTGAGCGGATAACAATTTCACACAGAATTCATTAAA
GAGGAGAAATTAACTATGGGCCATCATTAGAAGAATGGCGGTGCGAGCAGTAAAG
GAGAAGAACTTTTCACTGGAGTTGTCCCAATTCTTGTTGAATTAGATGGTGATGTT
AATGGGCACTAGTTTTCTGTCAGTGGAGAGGGTGAAGGTGATGCAACATACGGAA
AACTTACCCTTAAATTTATTTGCACTACTGGAAAACTACCTGTTCCATGGCCAACA
CTTGTCACTACTTTGACCTGGGGTGTTCAATGCTTTGCGAGATACCCAGATCATAT
GAAACAGCATGACTTTTTCAAGAGTGCCATGCCCGAAGGTTATGTACAGGAAAGA
ACTATATTTTTCAAAGATGACGGGAACTACAAGACACGTGCTGAAGTCAAGTTTG
AAGGTGATACCCTTGTTAATAGAATCGAGTTAAAAGGTATTGATTTTAAAGAAGAT
GGAAACATTCTTGGACACAAATTGGAATACAACTATATCTCAGACAATGTATACAT
CACGGCAGACAAACAAAAGAATGGAATCAAAGCGAACTTCAAAATTAGACACAAC
ATTGAAGATGGAGGTGTTCAACTAGCAGACCATTATCAACAAAATACTCCAATTG
GCGATGGCCCTGTCCTTTTACCAGACAACCATTACCTGTCCACCCAATCTAAGCTC
TCGAAAGATCCCAACGAAAAGAGAGACCACATGGTCCTTCTTGAGTTTGTAACAG
CTGCTGGGATTACACATGGCATGGATGAACTATACAAATAAGACTCCTGTTGATA
GATCCAGTAATGACCTCAGAACTCCATCTGGATTTGTTCAGAACGCTCGGTTGCC
GCCGGGCGTTTTTTATTGGTGAGAATCCAAGCTAGCTTGGCGAGATTTTCAGGAG
CTAAGGAAGCTAAATTTTTTTAAGGCAGTTATTGGTGCCCTTAAACGCCTGGGGT
AATGACTCTCTAGCTTGAGGCATCAAATAAAACGAAAGGCTCAGTCGAAAGACTG
GGCCTTTCGTTTTATCTGTTGTTTGTCGGTGAACGCTCTCCTGAGTAGGACAAATC
CGCCCTCTAGACTGGGTTGAAGGCTCTCAAGGGCATCGGTCGAGATCCCGGTGCC
TAATGAGTGAGCTAACTTACATTAATTGCGTTGCGCTCACTGCCCGCTTTCCAGTC
GGGAAACCTGTCGTGCCAGCTGCATTAATGAATCGGCCAACGCGCGGGGAGAGG
CGGTTTGCGTATTGGGCGCCAGGGTGGTTTTTCTTTTCACCAGTGAGACGGGCAA
CAGCTGATTGCCCTTCACCGCCTGGCCCTGAGAGAGTTGCAGCAAGCGGTCCACG
CTGGTTTGCCCCAGCAGGCGAAAATCCTGTTTGATGGTGGTTAACGGCGGGATAT
AACATGAGCTGTCTTCGGTATCGTCGTATCCCACTACCGAGATATCCGCACCAAC
GCGCAGCCCGGACTCGGTAATGGCGCGCATTGCGCCCAGCGCCATCTGATCGTTG
GCAACCAGCATCGCAGTGGGAACGATGCCCTCATTCAGCATTTGCATGGTTTGTT

GAAAACCGGACATGGCACTCCAGTCGCCTTCCCGTTCCGCTATCGGCTGAATTTG
ATTGCGAGTGAGATATTTATGCCAGCCAGCCAGACGCAGACGCGCCGAGACAGAA
CTTAATGGGCCCGCTAACAGCGCGATTTGCTGGTGACCCAATGCGACCAGATGCT
CCACGCCCAGTCGCGTACCGTCTTCATGGGAGAAAATAATACTGTTGATGGGTGT
CTGGTCAGAGACATCAAGAAATAACGCCGGAACATTAGTGCAGGCAGCTTCCACA
GCAATGGCATCCTGGTCATCCAGCGGATAGTTAATGATCAGCCCACTGACGCGTT
GCGCGAGAAGATTGTGCACCGCCGCTTTACAGGCTTCGACGCCGCTTCGTTCTAC
CATCGACACCACCACGCTGGCACCCAGTTGATCGGCGCGAGATTTAATCGCCGCG
ACAATTTGCGACGGCGCGTGCAGGGCCAGACTGGAGGTGGCAACGCCAATCAGC
AACGACTGTTTGCCCGCCAGTTGTTGTGCCACGCGGTTGGGAATGTAATTCAGCT
CCGCCATCGCCGCTTCCACTTTTTCCCGCGTTTTCGCAGAAACGTGGCTGGCCTG
GTTCACCACGCGGGAAACGGTCTGATAAGAGACACCGGCATACTCTGCGACATCG
TATAACGTTACTGGTTTCACATTCACCACCCTGAATTGACTCTCTTCCGGGCGCTA
TCATGCCATACCGCGAAAGGTTTTGCACCATTCGATGGTGTCCAATAACTGCCTTA
AAAAAATTACGCCCCGCCCTGCCACTCATCGCAGTACTGTTGTAATTCATTAAGCA
TTCTGCCGACATGGAAGCCATCACAGACGGCATGATGAACCTGAATCGCCAGCGG
CATCAGCACCTTGTCGCCTTGCGTATAATATTTGCCCATGGTGAAAACGGGGGCG
AAGAAGTTGTCCATATTGGCCACGTTTAAATCAAAACTGGTGAAACTCACCCAGG
GATTGGCTGAGACGAAAAACATATTCTCAATAAACCCTTTAGGGAAATAGGCCAG
GTTTTCACCGTAACACGCCACATCTTGCGAATATATGTGTAGAAACTGCCGGAAA
TCGTCGTGGTATTCACTCCAGAGCGATGAAAACGTTTCAGTTTGCTCATGGAAAA
CGGTGTAACAAGGGTGAACACTATCCCATATCACCAGCTCACCGTCTTTCATTGC
CATACGGAATTCCGGATGAGCATTCATCAGGCGGGCAAGAATGTGAATAAAGGCC
GGATAAAACTTGTGCTTATTTTTCTTTACGGTCTTTAAAAAGGCCGTAATATCCAG
CTGAACGGTCTGGTTATAGGTACATTGAGCAACTGACTGAAATGCCTCAAAATGT
TCTTTACGATGCCATTGGGATATATCAACGGTGGTATATCCAGTGATTTTTTTCTC
CATTTTAGCTTCCTTAGC

pFI3 -

TCCTGAAAATCTCGATAACTCAAAAAATACGCCCGGTAGTGATCTTATTTCATTAT
GGTGAAAGTTGGAACCTCTTACGTGCCGATCAACGTCTCATTTTCGCCAAAAGTT
GGCCCAGGGCTTCCCGGTATCAACAGGGACACCAGGATTTATTTATTCTGCGAAG
TGATCTTCCGTCACAGGTATTTATTCGGCGCAAAGTGCGTCGGGTGATGCTGCCA
ACTTACTGATTTAGTGTATGATGGTGTTTTTGAGGTGCTCCAGTGGCTTCTGTTTC
TATCAGCTGTCCCTCCTGTTCAGCTACTGACGGGGTGGTGCGTAACGGCAAAAGC
ACCGCCGGACATCAGCGCTAGCGGAGTGTATACTGGCTTACTATGTTGGCACTGA
TGAGGGTGTCAGTGAAGTGCTTCATGTGGCAGGAGAAAAAAGGCTGCACCGGTG
CGTCAGCAGAATATGTGATACAGGATATATTCCGCTTCCTCGCTCACTGACTCGCT
ACGCTCGGTCGTTCGACTGCGGCGAGCGGAAATGGCTTACGAACGGGGCGGAGA
TTTCCTGGAAGATGCCAGGAAGATACTTAACAGGGAAGTGAGAGGGCCGCGGCA
AAGCCGTTTTTCCATAGGCTCCGCCCCCCTGACAAGCATCACGAAATCTGACGCT
CAAATCAGTGGTGGCGAAACCCGACAGGACTATAAAGATACCAGGCGTTTCCCCC
TGGCGGCTCCCTCGTGCGCTCTCCTGTTCCTGCCTTTCGGTTTACCGGTGTCATTC
CGCTGTTATGGCCGCGTTTGTCTCATTCCACGCCTGACACTCAGTTCCGGGTAGG
CAGTTCGCTCCAAGCTGGACTGTATGCACGAACCCCCCGTTCAGTCCGACCGCTG
CGCCTTATCCGGTAACTATCGTCTTGAGTCCAACCCGGAAAGACATGCAAAAGCA
CCACTGGCAGCAGCCACTGGTAATTGATTTAGAGGAGTTAGTCTTGAAGTCATGC
GCCGGTTAAGGCTAAACTGAAAGGACAAGTTTTGGTGACTGCGCTCCTCCAAGCC
AGTTACCTCGGTTCAAAGAGTTGGTAGCTCAGAGAACCTTCGAAAAACCGCCCTG
CAAGGCGGTTTTTTCGTTTTCAGAGCAAGAGATTACGCGCAGACCAAAACGATCT
CAAGAAGATCATCTTATTAATCAGATAAAATATTTCTAGATTTCAGTGCAATTTAT
CTCTTCAAATGTAGCACCTGAAGTCAGCCCCATACGATATAAGTTGTAATTCTCAT
GTTTGACAGCTTATCATCGATAAGCTTGGTACCCAATTATGACAACTTGACGGCTA
CATCATTCACTTTTTCTTCACAACCGGCACGGAACTCGCTCGGGCTGGCCCCGGT

GCATTTTTTAAATACCCGCGAGAAATAGAGTTGATCGTCAAAACCAACATTGCGA
CCGACGGTGGCGATAGGCATCCGGGTGGTGCTCAAAAGCAGCTTCGCCTGGCTG
ATACGTTGGTCCTCGCGCCAGCTTAAGACGCTAATCCCTAACTGCTGGCGGAAAA
GATGTGACAGACGCGACGGCGACAAGCAAACATGCTGTGCGACGCTGGCGATAT
CAAAATTGCTGTCTGCCAGGTGATCGCTGATGTACTGACAAGCCTCGCGTACCCG
ATTATCCATCGGTGGATGGAGCGACTCGTTAATCGCTTCCATGCGCCGCAGTAAC
AATTGCTCAAGCAGATTTATCGCCAGCAGCTCCGAATAGCGCCCTTCCCCTTGCC
CGGCGTTAATGATTTGCCCAAACAGGTCGCTGAAATGCGGCTGGTGCGCTTCATC
CGGGCGAAAGAACCCCGTATTGGCAAATATTGACGGCCAGTTAAGCCATTCATGC
CAGTAGGCGCGCGGACGAAAGTAAACCCACTGGTGATACCATTCGCGAGCCTCCG
GATGACGACCGTAGTGATGAATCTCTCCTGGCGGGAACAGCAAAATATCACTCGG
TCGGCAAACAAATTCTCGTCCCTGATTTTTCACCACCCCCTGACCGCGAATGGTG
AGATTGAGAATATAACCTTTCATTCCCAGCGGTCGGTCGATAAAAAAATCGAGAT
AACCGTTGGCCTCAATCGGCGTTAAACCCGCCACCAGATGGGCATTAAACGAGTA
TCCCGGCAGCAGGGGATCATTTTGCGCTTCAGCCATACTTTTCATACTCCCGCCAT
TCAGAGAAGAAACCAATTGTCCATATTGCATCAGACATTGCCGTCACTGCGTCTTT
TACTGGCTCTTCTCGCTAACCAAACCGGTAACCCCGCTTATTAAAAGCATTCTGTA
ACAAAGCGGGACCAAAGCCATGACAAAAACGCGTAACAAAAGTGTCTATAATCAC
GGCAGAAAAGTCCACATTGATTATTTGCACGGCGTCACACTTTGCTATGCCATAG
CATTTTTATCCATAAGATTAGCGGATCCTACCTGACGCTTTTTATCGCAACTCTCT
ACTGTTTCTCCATACCCGTTTTTTTGGGCTAACAGGAGGAATTAGATCATGACGGT
CAAGTACACCGATGCTCAAATTCAACGTCTTCGCGAATATGGAAACGGAACCTAC
GAACAAAAGGTTTTCGAGGACTTAGCATCTCGTGACGCGGCTTTCAGTAAAGAGA
TGAGCGTGGCGTCAACTGACAATGAAAAAAAGATTAAAGGGATGATTGCAAATCC
ATCACGTCATGGTTTAACGCAGTTAATGAATGATATTGCAGACGCATTAGTGGCA
GAGGGCTTTATTGAAGTCCGTACGCCGATTTTCATCTCCAAAGATGCTTTGGCAC
GTATGACTATCACCGAAGACAAGCCCCTGTTTAAGCAAGTTTTCTGGATCGACGA
AAAGCGTGCACTTCGCCCTATGTTGGCACCAAACCTGGCCTCCGTACTGCGCGAC
TTACGCGATCACACCGACGGACCCGTGAAGATTTTCGAAATGGGATCATGTTTTC
GCAAGGAATCACATTCAGGGATGCATCTGGAGGAATTCACCATGTTGAACTTAGT
TGATATGGGACCGCGCGGCGATGCCACAGAAGTATTAAAAAACTACATCAGTGTC
GTAATGAAGGCTGCTGGTTTGCCTGATTATGATTTAGTACAAGAAGAAAGTGATG
TATACAAAGAAACAATTGATGTGGAAATCAACGGGCAAGAAGTATGCAGTGCCGC
AGTGGGGCCGATTCCGCTGGATGCGGCCCATGACGTGCATGAGCCTTGGTCTGGT
GCTGGCTTCGGGTTGGAGCGTCTTTTAACGATTCGTGAGAAATACTCAACGGTCA
AGAAAGGCGGCGCTTCCATCAGTTACTTGAACGGCGCCAAGATTAATGTCGACCA
TCATCATCATCATCATTGAGTTTAAACGGTCTCCAGCTTGGCTGTTTTGGCGGATG
AGAGAAGATTTTCAGCCTGATACAGATTAAATCAGAACGCAGAAGCGGTCTGATA
AAACAGAATTTGCCTGGCGGCAGTAGCGCGGTGGTCCCACCTGACCCCATGCCGA
ACTCAGAAGTGAAACGCCGTAGCGCCGATGGTAGTGTGGGGTCTCCCCATGCGA
GAGTAGGGAACTGCCAGGCATCAAATAAAACGAAAGGCTCAGTCGAAAGACTGG
GCCTTGTTTGTGAGCTCCCGGTCATCAATCATCCCCATAATCCTTGTTAGATTATC
AATTTTAAAAAACTAACAGTTGTCAGCCTGTCCCGCTTTAATATCATGTGAGCACC
GGTTTATTGACTACCGGAAGCAGTGTGACCGTGTGCTTCTCAAATGCCTGAGGCC
AGTTTGCTCAGGCTCTCCCCGTGGAGGTAATAATTGACGATATGATCAGTGCACG
GCTAACTAAGCGGCCTGCTGACTTTCTCGCCGATCAAAAGGCATTTTGCTATTAA
GGGATTGACGAGGGCGTATCTGCGCAGTAAGATGCGCCCCGCATTGGGGGACGG
TCCGGCGACCAGCGGGTCTCTAAAACCTAGCCAGCGGGGTTCGACGCCCCGGTCT
CTCGCCAAATTCGAAAAGCCTGCTCAACGAGCAGGCTTTTTTGCATGCTCGAGCA
GCTCAGTCATAAAAAATTTATTTGCTTTGTGAGCGGATAACAATTATAATAGATTC
AATTGTGAGCGGATAACAATTTCACACAGAATTCATTAAAGAGGAGAAATTAACT
ATGGGCCATCATTAGAAGAATGGCGGTGCGAGCAGTAAAGGAGAAGAACTTTTCA
CTGGAGTTGTCCCAATTCTTGTTGAATTAGATGGTGATGTTAATGGGCACTAGTTT
TCTGTCAGTGGAGAGGGTGAAGGTGATGCAACATACGGAAAACTTACCCTTAAAT
TTATTTGCACTACTGGAAAACTACCTGTTCCATGGCCAACACTTGTCACTACTTTG

ACCTGGGGTGTTCAATGCTTTGCGAGATACCCAGATCATATGAAACAGCATGACT
TTTTCAAGAGTGCCATGCCCGAAGGTTATGTACAGGAAAGAACTATATTTTTCAAA
GATGACGGGAACTACAAGACACGTGCTGAAGTCAAGTTTGAAGGTGATACCCTTG
TTAATAGAATCGAGTTAAAAGGTATTGATTTTAAAGAAGATGGAAACATTCTTGGA
CACAAATTGGAATACAACTATATCTCAGACAATGTATACATCACGGCAGACAAAC
AAAAGAATGGAATCAAAGCGAACTTCAAAATTAGACACAACATTGAAGATGGAGG
TGTTCAACTAGCAGACCATTATCAACAAAATACTCCAATTGGCGATGGCCCTGTCC
TTTTACCAGACAACCATTACCTGTCCACCCAATCTAAGCTCTCGAAAGATCCCAAC
GAAAAGAGAGACCACATGGTCCTTCTTGAGTTTGTAACAGCTGCTGGGATTACAC
ATGGCATGGATGAACTATACAAATAAGACTCCTGTTGATAGATCCAGTAATGACC
TCAGAACTCCATCTGGATTTGTTCAGAACGCTCGGTTGCCGCCGGGCGTTTTTTAT
TGGTGAGAATCCAAGCTAGCTTGGCGAGATTTTCAGGAGCTAAGGAAGCTAAATT
TTTTTAAGGCAGTTATTGGTGCCCTTAAACGCCTGGGGTAATGACTCTCTAGCTTG
AGGCATCAAATAAAACGAAAGGCTCAGTCGAAAGACTGGGCCTTTCGTTTTATCT
GTTGTTTGTCGGTGAACGCTCTCCTGAGTAGGACAAATCCGCCCTCTAGACTGGG
TTGAAGGCTCTCAAGGGCATCGGTCGAGATCCCGGTGCCTAATGAGTGAGCTAAC
TTACATTAATTGCGTTGCGCTCACTGCCCGCTTTCCAGTCGGGAAACCTGTCGTG
CCAGCTGCATTAATGAATCGGCCAACGCGCGGGGAGAGGCGGTTTGCGTATTGG
GCGCCAGGGTGGTTTTTCTTTTCACCAGTGAGACGGGCAACAGCTGATTGCCCTT
CACCGCCTGGCCCTGAGAGAGTTGCAGCAAGCGGTCCACGCTGGTTTGCCCCAGC
AGGCGAAAATCCTGTTTGATGGTGGTTAACGGCGGGATATAACATGAGCTGTCTT
CGGTATCGTCGTATCCCACTACCGAGATATCCGCACCAACGCGCAGCCCGGACTC
GGTAATGGCGCGCATTGCGCCCAGCGCCATCTGATCGTTGGCAACCAGCATCGCA
GTGGGAACGATGCCCTCATTCAGCATTTGCATGGTTTGTTGAAAACCGGACATGG
CACTCCAGTCGCCTTCCCGTTCCGCTATCGGCTGAATTTGATTGCGAGTGAGATA
TTTATGCCAGCCAGCCAGACGCAGACGCGCCGAGACAGAACTTAATGGGCCCGCT
AACAGCGCGATTTGCTGGTGACCCAATGCGACCAGATGCTCCACGCCCAGTCGCG
TACCGTCTTCATGGGAGAAAATAATACTGTTGATGGGTGTCTGGTCAGAGACATC
AAGAAATAACGCCGGAACATTAGTGCAGGCAGCTTCCACAGCAATGGCATCCTGG
TCATCCAGCGGATAGTTAATGATCAGCCCACTGACGCGTTGCGCGAGAAGATTGT
GCACCGCCGCTTTACAGGCTTCGACGCCGCTTCGTTCTACCATCGACACCACCAC
GCTGGCACCCAGTTGATCGGCGCGAGATTTAATCGCCGCGACAATTTGCGACGGC
GCGTGCAGGGCCAGACTGGAGGTGGCAACGCCAATCAGCAACGACTGTTTGCCC
GCCAGTTGTTGTGCCACGCGGTTGGGAATGTAATTCAGCTCCGCCATCGCCGCTT
CCACTTTTTCCCGCGTTTTCGCAGAAACGTGGCTGGCCTGGTTCACCACGCGGGA
AACGGTCTGATAAGAGACACCGGCATACTCTGCGACATCGTATAACGTTACTGGT
TTCACATTCACCACCCTGAATTGACTCTCTTCCGGGCGCTATCATGCCATACCGCG
AAAGGTTTTGCACCATTCGATGGTGTCCAATAACTGCCTTAAAAAAATTACGCCCC
GCCCTGCCACTCATCGCAGTACTGTTGTAATTCATTAAGCATTCTGCCGACATGGA
AGCCATCACAGACGGCATGATGAACCTGAATCGCCAGCGGCATCAGCACCTTGTC
GCCTTGCGTATAATATTTGCCCATGGTGAAAACGGGGGCGAAGAAGTTGTCCATA
TTGGCCACGTTTAAATCAAAACTGGTGAAACTCACCCAGGGATTGGCTGAGACGA
AAAACATATTCTCAATAAACCCTTTAGGGAAATAGGCCAGGTTTTCACCGTAACAC
GCCACATCTTGCGAATATATGTGTAGAAACTGCCGGAAATCGTCGTGGTATTCAC
TCCAGAGCGATGAAAACGTTTCAGTTTGCTCATGGAAAACGGTGTAACAAGGGTG
AACACTATCCCATATCACCAGCTCACCGTCTTTCATTGCCATACGGAATTCCGGAT
GAGCATTCATCAGGCGGGCAAGAATGTGAATAAAGGCCGGATAAAACTTGTGCTT
ATTTTTCTTTACGGTCTTTAAAAAGGCCGTAATATCCAGCTGAACGGTCTGGTTAT
AGGTACATTGAGCAACTGACTGAAATGCCTCAAAATGTTCTTTACGATGCCATTG
GGATATATCAACGGTGGTATATCCAGTGATTTTTTTCTCCATTTTAGCTTCCTTAG
C

pFI4 – aFAB120 promoter

**TCCTGAAAATCTCGATAACTCAAAAAATACGCCCGGTAGTGATCTTATTTCATTAT
GGTGAAAGTTGGAACCTCTTACGTGCCGATCAACGTCTCATTTTCGCCAAAAGTT
GGCCCAGGGCTTCCCGGTATCAACAGGGACACCAGGATTTATTTATTCTGCGAAG
TGATCTTCCGTCACAGGTATTTATTCGGCGCAAAGTGCGTCGGGTGATGCTGCCA
ACTTACTGATTTAGTGTATGATGGTGTTTTTGAGGTGCTCCAGTGGCTTCTGTTTC
TATCAGCTGTCCCTCCTGTTCAGCTACTGACGGGGTGGTGCGTAACGGCAAAAGC
ACCGCCGGACATCAGCGCTAGCGGAGTGTATACTGGCTTACTATGTTGGCACTGA
TGAGGGTGTCAGTGAAGTGCTTCATGTGGCAGGAGAAAAAAGGCTGCACCGGTG
CGTCAGCAGAATATGTGATACAGGATATATTCCGCTTCCTCGCTCACTGACTCGCT
ACGCTCGGTCGTTCGACTGCGGCGAGCGGAAATGGCTTACGAACGGGGCGGAGA
TTTCCTGGAAGATGCCAGGAAGATACTTAACAGGGAAGTGAGAGGGCCGCGGCA
AAGCCGTTTTTCCATAGGCTCCGCCCCCCTGACAAGCATCACGAAATCTGACGCT
CAAATCAGTGGTGGCGAAACCCGACAGGACTATAAAGATACCAGGCGTTTCCCCC
TGGCGGCTCCCTCGTGCGCTCTCCTGTTCCTGCCTTTCGGTTTACCGGTGTCATTC
CGCTGTTATGGCCGCGTTTGTCTCATTCCACGCCTGACACTCAGTTCCGGGTAGG
CAGTTCGCTCCAAGCTGGACTGTATGCACGAACCCCCCGTTCAGTCCGACCGCTG
CGCCTTATCCGGTAACTATCGTCTTGAGTCCAACCCGGAAAGACATGCAAAAGCA
CCACTGGCAGCAGCCACTGGTAATTGATTTAGAGGAGTTAGTCTTGAAGTCATGC
GCCGGTTAAGGCTAAACTGAAAGGACAAGTTTTGGTGACTGCGCTCCTCCAAGCC
AGTTACCTCGGTTCAAAGAGTTGGTAGCTCAGAGAACCTTCGAAAAACCGCCCTG
CAAGGCGGTTTTTTCGTTTTCAGAGCAAGAGATTACGCGCAGACCAAAACGATCT
CAAGAAGATCATCTTATTAATCAGATAAAATATTTCTAGATTTCAGTGCAATTTAT
CTCTTCAAATGTAGCACCTGAAGTCAGCCCCATACGATATAAGTTGTAATTCTCAT
GTTTGACAGCTTATCATCGATAAGCTTGGTACCCAATCGACATCGCATCTTTTTGT
ACCTATAATGTGTGGATACCCGTTTTTTTGGGCTAACAGGAGGAATTAGATCATG
ACGGTCAAGTACACCGATGCTCAAATTCAACGTCTTCGCGAATATGGAAACGGAA
CCTACGAACAAAAGGTTTTCGAGGACTTAGCATCTCGTGACGCGGCTTTCAGTAA
AGAGATGAGCGTGGCGTCAACTGACAATGAAAAAAAGATTAAAGGGATGATTGCA
AATCCATCACGTCATGGTTTAACGCAGTTAATGAATGATATTGCAGACGCATTAGT
GGCAGAGGGCTTTATTGAAGTCCGTACGCCGATTTTCATCTCCAAAGATGCTTTG
GCACGTATGACTATCACCGAAGACAAGCCCCTGTTTAAGCAAGTTTTCTGGATCG
ACGAAAAGCGTGCACTTCGCCCTATGTTGGCACCAAACCTGGCCTCCGTACTGCG
CGACTTACGCGATCACACCGACGGACCCGTGAAGATTTTCGAAATGGGATCATGT
TTTCGCAAGGAATCACATTCAGGGATGCATCTGGAGGAATTCACCATGTTGAACT
TAGTTGATATGGGACCGCGCGGCGATGCCACAGAAGTATTAAAAAACTACATCAG
TGTCGTAATGAAGGCTGCTGGTTTGCCTGATTATGATTTAGTACAAGAAGAAAGT
GATGTATACAAAGAAACAATTGATGTGGAAATCAACGGGCAAGAAGTATGCAGTG
CCGCAGTGGGGCCGATTCCGCTGGATGCGGCCCATGACGTGCATGAGCCTTGGT
CTGGTGCTGGCTTCGGGTTGGAGCGTCTTTTAACGATTCGTGAGAAATACTCAAC
GGTCAAGAAAGGCGGCGCTTCCATCAGTTACTTGAACGGCGCCAAGATTAATTGA
GTTTAAACGGTCTCCAGCTTGGCTGTTTTGGCGGATGAGAGAAGATTTTCAGCCT
GATACAGATTAAATCAGAACGCAGAAGCGGTCTGATAAAACAGAATTTGCCTGGC
GGCAGTAGCGCGGTGGTCCCACCTGACCCCATGCCGAACTCAGAAGTGAAACGC
CGTAGCGCCGATGGTAGTGTGGGGTCTCCCCATGCGAGAGTAGGGAACTGCCAG
GCATCAAATAAAACGAAAGGCTCAGTCGAAAGACTGGGCCTTGTTTGTGAGCTCC
CGGTCATCAATCATCCCCATAATCCTTGTTAGATTATCAATTTTAAAAAACTAACA
GTTGTCAGCCTGTCCCGCTTTAATATCATGTGAGCACCGGTTTATTGACTACCGGA
AGCAGTGTGACCGTGTGCTTCTCAAATGCCTGAGGCCAGTTTGCTCAGGCTCTCC
CCGTGGAGGTAATAATTGACGATATGATCAGTGCACGGCTAACTAAGCGGCCTGC
TGACTTTCTCGCCGATCAAAAGGCATTTTGCTATTAAGGGATTGACGAGGGCGTA
TCTGCGCAGTAAGATGCGCCCCGCATTGGGGGACGGTCCGGCGACCAGCGGGTC
TCTAAAACCTAGCCAGCGGGGTTCGACGCCCCGGTCTCTCGCCAAATTCGAAAAG
CCTGCTCAACGAGCAGGCTTTTTGCATGCTCGAGCAGCTCAGTCATAAAAAATTT
ATTTGCTTTGTGAGCGGATAACAATTATAATAGATTCAATTGTGAGCGGATAACAA
TTTCACACAGAATTCATTAAAGAGGAGAAATTAACTATGGGCCATCATTAGAAGA**

ATGGCGGTGCGAGCAGTAAAGGAGAAGAACTTTTCACTGGAGTTGTCCCAATTCT
TGTTGAATTAGATGGTGATGTTAATGGGCACAAATTTTCTGTCAGTGGAGAGGGT
GAAGGTGATGCAACATACGGAAAACTTACCCTTAAATTTATTTGCACTACTGGAAA
ACTACCTGTTCCATGGCCAACACTTGTCACTACTTTGACCTGGGGTGTTCAATGCT
TTGCGAGATACCCAGATCATATGAAACAGCATGACTTTTTCAAGAGTGCCATGCC
CGAAGGTTATGTACAGGAAAGAACTATATTTTTCAAAGATGACGGGAACTACAAG
ACACGTGCTGAAGTCAAGTTTGAAGGTGATACCCTTGTTAATAGAATCGAGTTAA
AAGGTATTGATTTTAAAGAAGATGGAAACATTCTTGGACACAAATTGGAATACAA
CTATATCTCAGACAATGTATACATCACGGCAGACAAACAAAAGAATGGAATCAAA
GCGAACTTCAAAATTAGACACAACATTGAAGATGGAGGTGTTCAACTAGCAGACC
ATTATCAACAAAATACTCCAATTGGCGATGGCCCTGTCCTTTTACCAGACAACCAT
TACCTGTCCACCCAATCTAAGCTCTCGAAAGATCCCAACGAAAAGAGAGACCACA
TGGTCCTTCTTGAGTTTGTAACAGCTGCTGGGATTACACATGGCATGGATGAACT
ATACAAATAAGACTCCTGTTGATAGATCCAGTAATGACCTCAGAACTCCATCTGGA
TTTGTTCAGAACGCTCGGTTGCCGCCGGGCGTTTTTTATTGGTGAGAATCCAAGC
TAGCTTGGCGAGATTTTCAGGAGCTAAGGAAGCTAAATTTTTTTAAGGCAGTTATT
GGTGCCCTTAAACGCCTGGGGTAATGACTCTCTAGCTTGAGGCATCAAATAAAAC
GAAAGGCTCAGTCGAAAGACTGGGCCTTTCGTTTTATCTGTTGTTTGTCGGTGAA
CGCTCTCCTGAGTAGGACAAATCCGCCCTCTAGACTGGGTTGAAGGCTCTCAAGG
GCATCGGTCGAGATCCCGGTGCCTAATGAGTGAGCTAACTTACATTAATTGCGTT
GCGCTCACTGCCCGCTTTCCAGTCGGGAAACCTGTCGTGCCAGCTGCATTAATGA
ATCGGCCAACGCGCGGGGAGAGGCGGTTTGCGTATTGGGCGCCAGGGTGGTTTT
TCTTTTCACCAGTGAGACGGGCAACAGCTGATTGCCCTTCACCGCCTGGCCCTGA
GAGAGTTGCAGCAAGCGGTCCACGCTGGTTTGCCCCAGCAGGCGAAAATCCTGTT
TGATGGTGGTTAACGGCGGGATATAACATGAGCTGTCTTCGGTATCGTCGTATCC
CACTACCGAGATATCCGCACCAACGCGCAGCCCGGACTCGGTAATGGCGCGCATT
GCGCCCAGCGCCATCTGATCGTTGGCAACCAGCATCGCAGTGGGAACGATGCCCT
CATTCAGCATTTGCATGGTTTGTTGAAAACCGGACATGGCACTCCAGTCGCCTTC
CCGTTCCGCTATCGGCTGAATTTGATTGCGAGTGAGATATTTATGCCAGCCAGCC
AGACGCAGACGCGCCGAGACAGAACTTAATGGGCCCGCTAACAGCGCGATTTGCT
GGTGACCCAATGCGACCAGATGCTCCACGCCCAGTCGCGTACCGTCTTCATGGGA
GAAAATAATACTGTTGATGGGTGTCTGGTCAGAGACATCAAGAAATAACGCCGGA
ACATTAGTGCAGGCAGCTTCCACAGCAATGGCATCCTGGTCATCCAGCGGATAGT
TAATGATCAGCCCACTGACGCGTTGCGCGAGAAGATTGTGCACCGCCGCTTTACA
GGCTTCGACGCCGCTTCGTTCTACCATCGACACCACCACGCTGGCACCCAGTTGA
TCGGCGCGAGATTTAATCGCCGCGACAATTTGCGACGGCGCGTGCAGGGCCAGA
CTGGAGGTGGCAACGCCAATCAGCAACGACTGTTTGCCCGCCAGTTGTTGTGCCA
CGCGGTTGGGAATGTAATTCAGCTCCGCCATCGCCGCTTCCACTTTTTCCCGCGT
TTTCGCAGAAACGTGGCTGGCCTGGTTCACCACGCGGGAAACGGTCTGATAAGAG
ACACCGGCATACTCTGCGACATCGTATAACGTTACTGGTTTCACATTCACCACCCT
GAATTGACTCTCTTCCGGGCGCTATCATGCCATACCGCGAAAGGTTTTGCACCAT
TCGATGGTGTCCAATAACTGCCTTAAAAAAATTACGCCCCGCCCTGCCACTCATC
GCAGTACTGTTGTAATTCATTAAGCATTCTGCCGACATGGAAGCCATCACAGACG
GCATGATGAACCTGAATCGCCAGCGGCATCAGCACCTTGTCGCCTTGCGTATAAT
ATTTGCCCATGGTGAAAACGGGGGCGAAGAAGTTGTCCATATTGGCCACGTTTAA
ATCAAAACTGGTGAAACTCACCCAGGGATTGGCTGAGACGAAAAACATATTCTCA
ATAAACCCTTTAGGGAAATAGGCCAGGTTTTCACCGTAACACGCCACATCTTGCG
AATATATGTGTAGAAACTGCCGGAAATCGTCGTGGTATTCACTCCAGAGCGATGA
AAACGTTTCAGTTTGCTCATGGAAAACGGTGTAACAAGGGTGAACACTATCCCAT
ATCACCAGCTCACCGTCTTTCATTGCCATACGGAATTCCGGATGAGCATTCATCAG
GCGGGCAAGAATGTGAATAAAGGCCGGATAAAACTTGTGCTTATTTTTCTTTACG
GTCTTTAAAAAGGCCGTAATATCCAGCTGAACGGTCTGGTTATAGGTACATTGAG
CAACTGACTGAAATGCCTCAAAATGTTCTTTACGATGCCATTGGGATATATCAACG
GTGGTATATCCAGTGATTTTTTTCTCCATTTTAGCTTCCTTAGC

pFI5 – tetA/tetB/tetR

CCATCGAATGGCCAGATGATTAATTCCTAATTTTTGTTGACACTCTATCATTGATA
GAGTTATTTTACCACTCCCTATCAGTGATAGAGAAAAGTGAAATGAATAGTTCGAC
AAAAATCTAGATAACGAGGGCAAAAAAGGAGGAATTAGATCATGACGGTCAAGTA
CACCGATGCTCAAATTCAACGTCTTCGCGAATATGGAAACGGAACCTACGAACAA
AAGGTTTTCGAGGACTTAGCATCTCGTGACGCGGCTTTCAGTAAAGAGATGAGCG
TGGCGTCAACTGACAATGAAAAAAAGATTAAAGGGATGATTGCAAATCCATCACG
TCATGGTTTAACGCAGTTAATGAATGATATTGCAGACGCATTAGTGGCAGAGGGC
TTTATTGAAGTCCGTACGCCGATTTTCATCTCCAAAGATGCTTTGGCACGTATGAC
TATCACCGAAGACAAGCCCCTGTTTAAGCAAGTTTTCTGGATCGACGAAAAGCGT
GCACTTCGCCCTATGTTGGCACCAAACCTGGCCTCCGTACTGCGCGACTTACGCG
ATCACACCGACGGACCCGTGAAGATTTTCGAAATGGGATCATGTTTTCGCAAGGA
ATCACATTCAGGGATGCATCTGGAGGAATTCACCATGTTGAACTTAGTTGATATG
GGACCGCGCGGCGATGCCACAGAAGTATTAAAAAACTACATCAGTGTCGTAATGA
AGGCTGCTGGTTTGCCTGATTATGATTTAGTACAAGAAGAAAGTGATGTATACAA
AGAAACAATTGATGTGGAAATCAACGGGCAAGAAGTATGCAGTGCCGCAGTGGG
GCCGATTCCGCTGGATGCGGCCCATGACGTGCATGAGCCTTGGTCTGGTGCTGGC
TTCGGGTTGGAGCGTCTTTTAACGATTCGTGAGAAATACTCAACGGTCAAGAAAG
GCGGCGCTTCCATCAGTTACTTGAACGGCGCCAAGATTAATGTCGACCATCATCA
TCATCATCATTGAGTTTAAACGGTCTCCAGCTTGGCTGTTTTGGCGGATGAGAGA
AGATTTTCAGCCTGATACAGATTAAATCAGAACGCAGAAGCGGTCTGATAAAACA
GAATTTGCCTGGCGGCAGTAGCGCGGTGGTCCCACCTGACCCCATGCCGAACTCA
GAAGTGAAACGCCGTAGCGCCGATGGTAGTGTGGGGTCTCCCCATGCGAGAGTA
GGGAACTGCCAGGCATCAAATAAAACGAAAGGCTCAGTCGAAAGACTGGGCCTTG
TTTGTGAGCTCCCGGTCATCAATCATCCCCATAATCCTTGTTAGATTATCAATTTT
AAAAAACTAACAGTTGTCAGCCTGTCCCGCTTTAATATCATGTGAGCACCGGTTTA
TTGACTACCGGAAGCAGTGTGACCGTGTGCTTCTCAAATGCCTGAGGCCAGTTTG
CTCAGGCTCTCCCCGTGGAGGTAATAATTGACGATATGATCAGTGCACGGCTAAC
TAAGCGGCCTGCTGACTTTCTCGCCGATCAAAAGGCATTTTGCTATTAAGGGATT
GACGAGGGCGTATCTGCGCAGTAAGATGCGCCCCGCATTGGGGGACGGTCCGGC
GACCAGCGGGTCTCTAAAACCTAGCCAGCGGGGTTCGACGCCCCGGTCTCTCGCC
AAATTCGAAAAGCCTGCTCAACGAGCAGGCTTTTTTGCATGCTCGAGCAGCTCAG
TCATAAAAAATTTATTTGCTTTGTGAGCGGATAACAATTATAATAGATTCAATTGT
GAGCGGATAACAATTTCACACAGAATTCATTAAAGAGGAGAAATTAACTATGGGC
CATCATTAGAAGAATGGCGGTGCGAGCAGTAAAGGAGAAGAACTTTTCACTGGAG
TTGTCCCAATTCTTGTTGAATTAGATGGTGATGTTAATGGGCACTAGTTTTCTGTC
AGTGGAGAGGGTGAAGGTGATGCAACATACGGAAAACTTACCCTTAAATTTATTT
GCACTACTGGAAAACTACCTGTTCCATGGCCAACACTTGTCACTACTTTGACCTGG
GGTGTTCAATGCTTTGCGAGATACCCAGATCATATGAAACAGCATGACTTTTTCAA
GAGTGCCATGCCCGAAGGTTATGTACAGGAAAGAACTATATTTTTCAAAGATGAC
GGGAACTACAAGACACGTGCTGAAGTCAAGTTTGAAGGTGATACCCTTGTTAATA
GAATCGAGTTAAAAGGTATTGATTTTAAAGAAGATGGAAACATTCTTGGACACAA
ATTGGAATACAACTATATCTCAGACAATGTATACATCACGGCAGACAAACAAAAG
AATGGAATCAAAGCGAACTTCAAAATTAGACACAACATTGAAGATGGAGGTGTTC
AACTAGCAGACCATTATCAACAAAATACTCCAATTGGCGATGGCCCTGTCCTTTTA
CCAGACAACCATTACCTGTCCACCCAATCTAAGCTCTCGAAAGATCCCAACGAAA
AGAGAGACCACATGGTCCTTCTTGAGTTTGTAACAGCTGCTGGGATTACACATGG
CATGGATGAACTATACAAATAAGACTCCTGTTGATAGATCCAGTAATGACCTCAG
AACTCCATCTGGATTTGTTCAGAACGCTCGGTTGCCGCCGGGCGTTTTTTATTGGT
GAGAATCCAAGCTAGCTTGGCGAGATTTTCAGGAGCTAAGGAAGCTAAATTTTTT
TAAGGCAGTTATTGGTGCCCTTAAACGCCTGGGGTAATGACTCTCTAGCTTGAGG
CATCAAATAAAACGAAAGGCTCAGTCGAAAGACTGGGCCTTTCGTTTTATCTGTT
GTTTGTCGGTGAACGCTCTCCTGAGTAGGACAAATCCGCCCTCTAGACTGGGTTG
AAGGCTCTCAAGGGCATCGGTCGAGATCCCGGTGCCTAATGAGTGAGCTAACTTA
CATTAATTGCGTTGCGCTCACTGCCCGCTTTCCAGTCGGGAAACCTGTCGTGCCA

GCTGCATTAATGAATCGGCCAACGCGCGGGGAGAGGCGGTTTGCGTATTGGGCG
CCAGGGTGGTTTTTCTTTTCACCAGTGAGACGGGCAACAGCTGATTGCCCTTCAC
CGCCTGGCCCTGAGAGAGTTGCAGCAAGCGGTCCACGCTGGTTTGCCCCAGCAG
GCGAAAATCCTGTTTGATGGTGGTTAACGGCGGGATATAACATGAGCTGTCTTCG
GTATCGTCGTATCCCACTACCGAGATATCCGCACCAACGCGCAGCCCGGACTCGG
TAATGGCGCGCATTGCGCCCAGCGCCATCTGATCGTTGGCAACCAGCATCGCAGT
GGGAACGATGCCCTCATTCAGCATTTGCATGGTTTGTTGAAAACCGGACATGGCA
CTCCAGTCGCCTTCCCGTTCCGCTATCGGCTGAATTTGATTGCGAGTGAGATATTT
ATGCCAGCCAGCCAGACGCAGACGCGCCGAGACAGAACTTAATGGGCCCGCTAA
CAGCGCGATTTGCTGGTGACCCAATGCGACCAGATGCTCCACGCCCAGTCGCGTA
CCGTCTTCATGGGAGAAAATAATACTGTTGATGGGTGTCTGGTCAGAGACATCAA
GAAATAACGCCGGAACATTAGTGCAGGCAGCTTCCACAGCAATGGCATCCTGGTC
ATCCAGCGGATAGTTAATGATCAGCCCACTGACGCGTTGCGCGAGAAGATTGTGC
ACCGCCGCTTTACAGGCTTCGACGCCGCTTCGTTCTACCATCGACACCACCACGC
TGGCACCCAGTTGATCGGCGCGAGATTTAATCGCCGCGACAATTTGCGACGGCGC
GTGCAGGGCCAGACTGGAGGTGGCAACGCCAATCAGCAACGACTGTTTGCCCGC
CAGTTGTTGTGCCACGCGGTTGGGAATGTAATTCAGCTCAGCCATCGCCGCTTCC
ACTTTTTCCCGCGTTTTCGCAGAAACGTGGCTGGCCTGGTTCACCACGCGGGAAA
CGGTCTGATAAGAGACACCGGCATACTCTGCGACATCGTATAACGTTACTGGTTT
CACATTCACCACCCTGAATTGACTCTCTTCCGGGCGCTATCATGCCATACCGCGA
AAGGTTTTGCACCATTCGATGGTGTCTTAACAAAATTTGGCGTTAACAAAATTTGG
CGAAAATGAGACGTTTAACAAAATTTGGCGAAAATGAGACGTTGATCGGCACGTA
AGAGGTTCCAACTTTCACCATAATGAAATAAGATCACTACCGGGCGTATTTTTTGA
GTTATCGAGATTTTCAGGAGCTAAGGAAGCTAAAATGGAGAAAAAAATCACTGGA
TATACCACCGTTGATATATCCCAATGGCATCGTAAAGAACATTTTGAGGCATTTCA
GTCAGTTGCTCAATGTACCTATAACCAGACCGTTCAGCTGGATATTACGGCCTTTT
TAAAGACCGTAAAGAAAAATAAGCACAAGTTTTATCCGGCCTTTATTCACATTCTT
GCCCGCCTGATGAATGCTCATCCGGAGTTCCGTATGGCAATGAAAGACGGTGAGC
TGGTGATATGGGATAGTGTTCACCCTTGTTACACCGTTTTCCATGAGCAAACTGAA
ACGTTTTCATCGCTCTGGAGTGAATACCACGACGATTTCCGGCAGTTTCTACACAT
ATATTCGCAAGATGTGGCGTGTTACGGTGAAAACCTGGCCTATTTCCCTAAAGGG
TTTATTGAGAATATGTTTTTCGTCTCAGCCAATCCCTGGGTGAGTTTCACCAGTTT
TGATTTAAACGTGGCCAATATGGACAACTTCTTCGCCCCCGTTTTCACTATGGGCA
AATATTATACGCAAGGCGACAAGGTGCTGATGCCGCTGGCGATTCAGGTTCATCA
TGCCGTTTGTGATGGCTTCCATGTCGGCAGAATGCTTAATGAATTACAACAGTACT
GCGATGAGTGGCAGGGCGGGGCGTAATAGGAATTAATGATGTCTCGTTTAGATAA
AAGTAAAGTGATTAACAGCGCATTAGAGCTGCTTAATGAGGTCGGAATCGAAGGT
TTAACAACCCGTAAACTCGCCCAGAAGCTAGGTGTAGAGCAGCCTACATTGTATT
GGCATGTAAAAAATAAGCGGGCTTTGCTCGACGCCTTAGCCATTGAGATGTTAGA
TAGGCACCATACTCACTTTTGCCCTTTAGAAGGGGAAAGCTGGCAAGATTTTTTAC
GTAATAACGCTAAAAGTTTTAGATGTGCTTTACTAAGTCATCGCGATGGAGCAAA
AGTACATTTAGGTACACGGCCTACAGAAAAACAGTATGAAACTCTCGAAAATCAA
TTAGCCTTTTTATGCCAACAAGGTTTTTCACTAGAGAATGCATTATATGCACTCAG
CGCAGTGGGGCATTTTACTTTAGGTTGCGTATTGGAAGATCAAGAGCATCAAGTC
GCTAAAGAAGAAAGGGAAACACCTACTACTGATAGTATGCCGCCATTATTACGAC
AAGCTATCGAATTATTTGATCACCAAGGTGCAGAGCCAGCCTTCTTATTCGGCCTT
GAATTGATCATATGCGGATTAGAAAAACAACTTAAATGTGAAAGTGGGTCTTAAA
AGCAGCATAACCTTTTTCCGTGATGGTAACTTCACTAGTTTAAAAGGATCTAGGTG
AAGATCCTTTTTGATAATCTCATGACCAAAATCCCTTAACGTGAGTTTTCGTTCCA
CTGAGCGTCAGACCCCGTAGAAAAGATCAAAGGATCTTCTGATTAATAAGATGAT
CTTCTTGAGATCGTTTTGGTCTGCGCGTAATCTCTTGCTCTGAAAACGAAAAAACC
GCCTTGCAGGGCGGTTTTTCGAAGGTTCTCTGAGCTACCAACTCTTTGAACCGAG
GTAACTGGCTTGGAGGAGCGCAGTCACCAAAACTTGTCCTTTCAGTTTAGCCTTA
ACCGGCGCATGACTTCAAGACTAACTCCTCTAAATCAATTACCAGTGGCTGCTGC
CAGTGGTGCTTTTGCATGTCTTTCCGGGTTGGACTCAAGACGATAGTTACCGGAT

AAGGCGCAGCGGTCGGACTGAACGGGGGGTTCGTGCATACAGTCCAGCTTGGAG
CGAACTGCCTACCCGGAACTGAGTGTCAGGCGTGGAATGAGACAAACGCGGCCA
TAACAGCGGAATGACACCGGTAAACCGAAAGGCAGGAACAGGAGAGCGCACGAG
GGAGCCGCCAGGGGGAAACGCCTGGTATCTTTATAGTCCTGTCGGGTTTCGCCAC
CACTGATTTGAGCGTCAGATTTCGTGATGCTTGTCAGGGGGGCGGAGCCTATGGA
AAAACGGCTTTGCCGCGGCCCTAACGCCAGCAACGCGGCCTTTTTACGGTTCCTG
GCCTTTTGCTGGCCTTTTGCTCACATGACCCGACA

pFI6 – aFAB120 promoter, aFAB83 promoter

TCCTGAAAATCTCGATAACTCAAAAAATACGCCCGGTAGTGATCTTATTTCATTAT
GGTGAAAGTTGGAACCTCTTACGTGCCGATCAACGTCTCATTTTCGCCAAAAGTT
GGCCCAGGGCTTCCCGGTATCAACAGGGACACCAGGATTTATTTATTCTGCGAAG
TGATCTTCCGTCACAGGTATTTATTCGGCGCAAAGTGCGTCGGGTGATGCTGCCA
ACTTACTGATTTAGTGTATGATGGTGTTTTTGAGGTGCTCCAGTGGCTTCTGTTTC
TATCAGCTGTCCCTCCTGTTCAGCTACTGACGGGGTGGTGCGTAACGGCAAAAGC
ACCGCCGGACATCAGCGCTAGCGGAGTGTATACTGGCTTACTATGTTGGCACTGA
TGAGGGTGTCAGTGAAGTGCTTCATGTGGCAGGAGAAAAAAGGCTGCACCGGTG
CGTCAGCAGAATATGTGATACAGGATATATTCCGCTTCCTCGCTCACTGACTCGCT
ACGCTCGGTCGTTCGACTGCGGCGAGCGGAAATGGCTTACGAACGGGGCGGAGA
TTTCCTGGAAGATGCCAGGAAGATACTTAACAGGGAAGTGAGAGGGCCGCGGCA
AAGCCGTTTTTCCATAGGCTCCGCCCCCCTGACAAGCATCACGAAATCTGACGCT
CAAATCAGTGGTGGCGAAACCCGACAGGACTATAAAGATACCAGGCGTTTCCCCC
TGGCGGCTCCCTCGTGCGCTCTCCTGTTCCTGCCTTTCGGTTTACCGGTGTCATTC
CGCTGTTATGGCCGCGTTTGTCTCATTCCACGCCTGACACTCAGTTCCGGGTAGG
CAGTTCGCTCCAAGCTGGACTGTATGCACGAACCCCCCGTTCAGTCCGACCGCTG
CGCCTTATCCGGTAACTATCGTCTTGAGTCCAACCCGGAAAGACATGCAAAAGCA
CCACTGGCAGCAGCCACTGGTAATTGATTTAGAGGAGTTAGTCTTGAAGTCATGC
GCCGGTTAAGGCTAAACTGAAAGGACAAGTTTTGGTGACTGCGCTCCTCCAAGCC
AGTTACCTCGGTTCAAAGAGTTGGTAGCTCAGAGAACCTTCGAAAAACCGCCCTG
CAAGGCGGTTTTTTCGTTTTCAGAGCAAGAGATTACGCGCAGACCAAAACGATCT
CAAGAAGATCATCTTATTAATCAGATAAAATATTTCTAGATTTCAGTGCAATTTAT
CTCTTCAAATGTAGCACCTGAAGTCAGCCCCATACGATATAAGTTGTAATTCTCAT
GTTTGACAGCTTATCATCGATAAGCTTGGTACCCAATCGACATCGCATCTTTTTGT
ACCTATAATGTGTGGATACCCGTTTTTTTGGGCTAACAGGAGGAATTAGATCATG
ACGGTCAAGTACACCGATGCTCAAATTCAACGTCTTCGCGAATATGGAAACGGAA
CCTACGAACAAAAGGTTTTCGAGGACTTAGCATCTCGTGACGCGGCTTTCAGTAA
AGAGATGAGCGTGGCGTCAACTGACAATGAAAAAAAGATTAAAGGGATGATTGCA
AATCCATCACGTCATGGTTTAACGCAGTTAATGAATGATATTGCAGACGCATTAGT
GGCAGAGGGCTTTATTGAAGTCCGTACGCCGATTTTCATCTCCAAAGATGCTTTG
GCACGTATGACTATCACCGAAGACAAGCCCCTGTTTAAGCAAGTTTTCTGGATCG
ACGAAAAGCGTGCACTTCGCCCTATGTTGGCACCAAACCTGGCCTCCGTACTGCG
CGACTTACGCGATCACACCGACGGACCCGTGAAGATTTTCGAAATGGGATCATGT
TTTCGCAAGGAATCACATTCAGGGATGCATCTGGAGGAATTCACCATGTTGAACT
TAGTTGATATGGGACCGCGCGGCGATGCCACAGAAGTATTAAAAAACTACATCAG
TGTCGTAATGAAGGCTGCTGGTTTGCCTGATTATGATTTAGTACAAGAAGAAAGT
GATGTATACAAAGAAACAATTGATGTGGAAATCAACGGGCAAGAAGTATGCAGTG
CCGCAGTGGGGCCGATTCCGCTGGATGCGGCCCATGACGTGCATGAGCCTTGGT
CTGGTGCTGGCTTCGGGTTGGAGCGTCTTTTAACGATTCGTGAGAAATACTCAAC
GGTCAAGAAAGGCGGCGCTTCCATCAGTTACTTGAACGGCGCCAAGATTAATTGA
GTTTAAACGGTCTCCAGCTTGGCTGTTTTGGCGGATGAGAGAAGATTTTCAGCCT
GATACAGATTAAATCAGAACGCAGAAGCGGTCTGATAAAACAGAATTTGCCTGGC
GGCAGTAGCGCGGTGGTCCCACCTGACCCCATGCCGAACTCAGAAGTGAAACGC
CGTAGCGCCGATGGTAGTGTGGGGTCTCCCCATGCGAGAGTAGGGAACTGCCAG
GCATCAAATAAAACGAAAGGCTCAGTCGAAAGACTGGGCCTTGTTTGTGAGCTCC

CGGTCATCAATCATCCCCATAATCCTTGTTAGATTATCAATTTTAAAAAACTAACA
GTTGTCAGCCTGTCCCGCTTTAATATCATGTGAGCACCGGTTTATTGACTACCGGA
AGCAGTGTGACCGTGTGCTTCTCAAATGCCTGAGGCCAGTTTGCTCAGGCTCTCC
CCGTGGAGGTAATAATTGACGATATGATCAGTGCACGGCTAACTAAGCGGCCTGC
TGACTTTCTCGCCGATCAAAAAAAAATTTATTTGCTTTAAAGTCTAACCTATAGGT
AGATTTAACGTATGGGGGACGGTCCGGCGACCAGCGGGTCTCTAAAACCTAGCCA
GCGGGGTTCGACGCCCCGGTCTCTCGCCAAATTCGAAAAGCCTGCTCAACGAGCA
GGCTTTTTTGCATGCTCGAGCAGCTCAGTCATAAAAAATTTATTTGCTTTGTGAGC
GGATAACAATTATAATAGATTCAATTGTGAGCGGATAACAATTTCACACAGAATTC
ATTAAAGAGGAGAAATTAACTATGAGTAAAGGAGAAGAACTTTTCACTGGAGTTG
TCCCAATTCTTGTTGAATTAGATGGTGATGTTAATGGGCACTAGTTTTCTGTCAGT
GGAGAGGGTGAAGGTGATGCAACATACGGAAAACTTACCCTTAAATTTATTTGCA
CTACTGGAAAACTACCTGTTCCATGGCCAACACTTGTCACTACTTTGACCTGGGGT
GTTCAATGCTTTGCGAGATACCCAGATCATATGAAACAGCATGACTTTTTCAAGAG
TGCCATGCCCGAAGGTTATGTACAGGAAAGAACTATATTTTTCAAAGATGACGGG
AACTACAAGACACGTGCTGAAGTCAAGTTTGAAGGTGATACCCTTGTTAATAGAA
TCGAGTTAAAAGGTATTGATTTTAAAGAAGATGGAAACATTCTTGGACACAAATTG
GAATACAACTATATCTCAGACAATGTATACATCACGGCAGACAAACAAAAGAATG
GAATCAAAGCGAACTTCAAAATTAGACACAACATTGAAGATGGAGGTGTTCAACT
AGCAGACCATTATCAACAAAATACTCCAATTGGCGATGGCCCTGTCCTTTTACCAG
ACAACCATTACCTGTCCACCCAATCTAAGCTCTCGAAAGATCCCAACGAAAAGAG
AGACCACATGGTCCTTCTTGAGTTTGTAACAGCTGCTGGGATTACACATGGCATG
GATGAACTATACAAATAAGACTCCTGTTGATAGATCCAGTAATGACCTCAGAACTC
CATCTGGATTTGTTCAGAACGCTCGGTTGCCGCCGGGCGTTTTTTATTGGTGAGA
ATCCAAGCTAGCTTGGCGAGATTTTCAGGAGCTAAGGAAGCTAAATTTTTTTAAG
GCAGTTATTGGTGCCCTTAAACGCCTGGGGTAATGACTCTCTAGCTTGAGGCATC
AAATAAAACGAAAGGCTCAGTCGAAAGACTGGGCCTTTCGTTTTATCTGTTGTTTG
TCGGTGAACGCTCTCCTGAGTAGGACAAATCCGCCCTCTAGACTGGGTTGAAGGC
TCTCAAGGGCATCGGTCGAGATCCCGGTGCCTAATGAGTGAGCTAACTTACATTA
ATTGCGTTGCGCTCACTGCCCGCTTTCCAGTCGGGAAACCTGTCGTGCCAGCTGC
ATTAATGAATCGGCCAACGCGCGGGGAGAGGCGGTTTGCGTATTGGGCGCCAGG
GTGGTTTTTCTTTTCACCAGTGAGACGGGCAACAGCTGATTGCCCTTCACCGCCT
GGCCCTGAGAGAGTTGCAGCAAGCGGTCCACGCTGGTTTGCCCCAGCAGGCGAA
AATCCTGTTTGATGGTGGTTAACGGCGGGATATAACATGAGCTGTCTTCGGTATC
GTCGTATCCCACTACCGAGATATCCGCACCAACGCGCAGCCCGGACTCGGTAATG
GCGCGCATTGCGCCCAGCGCCATCTGATCGTTGGCAACCAGCATCGCAGTGGGAA
CGATGCCCTCATTCAGCATTTGCATGGTTTGTTGAAAACCGGACATGGCACTCCA
GTCGCCTTCCCGTTCCGCTATCGGCTGAATTTGATTGCGAGTGAGATATTTATGCC
AGCCAGCCAGACGCAGACGCGCCGAGACAGAACTTAATGGGCCCGCTAACAGCG
CGATTTGCTGGTGACCCAATGCGACCAGATGCTCCACGCCCAGTCGCGTACCGTC
TTCATGGGAGAAAATAATACTGTTGATGGGTGTCTGGTCAGAGACATCAAGAAAT
AACGCCGGAACATTAGTGCAGGCAGCTTCCACAGCAATGGCATCCTGGTCATCCA
GCGGATAGTTAATGATCAGCCCACTGACGCGTTGCGCGAGAAGATTGTGCACCGC
CGCTTTACAGGCTTCGACGCCGCTTCGTTCTACCATCGACACCACCACGCTGGCA
CCCAGTTGATCGGCGCGAGATTTAATCGCCGCGACAATTTGCGACGGCGCGTGCA
GGGCCAGACTGGAGGTGGCAACGCCAATCAGCAACGACTGTTTGCCCGCCAGTT
GTTGTGCCACGCGGTTGGGAATGTAATTCAGCTCCGCCATCGCCGCTTCCACTTT
TTCCCGCGTTTTCGCAGAAACGTGGCTGGCCTGGTTCACCACGCGGGAAACGGTC
TGATAAGAGACACCGGCATACTCTGCGACATCGTATAACGTTACTGGTTTCACATT
CACCACCCTGAATTGACTCTCTTCCGGGCGCTATCATGCCATACCGCGAAAGGTT
TTGCACCATTCGATGGTGTCCAATAACTGCCTTAAAAAAATTACGCCCCGCCCTGC
CACTCATCGCAGTACTGTTGTAATTCATTAAGCATTCTGCCGACATGGAAGCCATC
ACAGACGGCATGATGAACCTGAATCGCCAGCGGCATCAGCACCTTGTCGCCTTGC
GTATAATATTTGCCCATGGTGAAAACGGGGGCGAAGAAGTTGTCCATATTGGCCA
CGTTTAAATCAAAACTGGTGAAACTCACCCAGGGATTGGCTGAGACGAAAAACAT

ATTCTCAATAAACCCTTTAGGGAAATAGGCCAGGTTTTCACCGTAACACGCCACAT<br>CTTGCGAATATATGTGTAGAAACTGCCGGAAATCGTCGTGGTATTCACTCCAGAG<br>CGATGAAAACGTTTCAGTTTGCTCATGGAAAACGGTGTAACAAGGGTGAACACTA<br>TCCCATATCACCAGCTCACCGTCTTTCATTGCCATACGGAATTCCGGATGAGCATT<br>CATCAGGCGGGCAAGAATGTGAATAAAGGCCGGATAAAACTTGTGCTTATTTTTC<br>TTTACGGTCTTTAAAAAGGCCGTAATATCCAGCTGAACGGTCTGGTTATAGGTAC<br>ATTGAGCAACTGACTGAAATGCCTCAAAATGTTCTTTACGATGCCATTGGGATATA<br>TCAACGGTGGTATATCCAGTGATTTTTTTCTCCATTTTAGCTTCCTTAGC

pFI7 – aFAB120 promoter
TCCTGAAAATCTCGATAACTCAAAAAATACGCCCGGTAGTGATCTTATTTCATTAT<br>GGTGAAAGTTGGAACCTCTTACGTGCCGATCAACGTCTCATTTTCGCCAAAAGTT<br>GGCCCAGGGCTTCCCGGTATCAACAGGGACACCAGGATTTATTTATTCTGCGAAG<br>TGATCTTCCGTCACAGGTATTTATTCGGCGCAAAGTGCGTCGGGTGATGCTGCCA<br>ACTTACTGATTTAGTGTATGATGGTGTTTTTGAGGTGCTCCAGTGGCTTCTGTTTC<br>TATCAGCTGTCCCTCCTGTTCAGCTACTGACGGGGTGGTGCGTAACGGCAAAAGC<br>ACCGCCGGACATCAGCGCTAGCGGAGTGTATACTGGCTTACTATGTTGGCACTGA<br>TGAGGGTGTCAGTGAAGTGCTTCATGTGGCAGGAGAAAAAAGGCTGCACCGGTG<br>CGTCAGCAGAATATGTGATACAGGATATATTCCGCTTCCTCGCTCACTGACTCGCT<br>ACGCTCGGTCGTTCGACTGCGGCGAGCGGAAATGGCTTACGAACGGGGCGGAGA<br>TTTCCTGGAAGATGCCAGGAAGATACTTAACAGGGAAGTGAGAGGGCCGCGGCA<br>AAGCCGTTTTTCCATAGGCTCCGCCCCCCTGACAAGCATCACGAAATCTGACGCT<br>CAAATCAGTGGTGGCGAAACCCGACAGGACTATAAAGATACCAGGCGTTTCCCCC<br>TGGCGGCTCCCTCGTGCGCTCTCCTGTTCCTGCCTTTCGGTTTACCGGTGTCATTC<br>CGCTGTTATGGCCGCGTTTGTCTCATTCCACGCCTGACACTCAGTTCCGGGTAGG<br>CAGTTCGCTCCAAGCTGGACTGTATGCACGAACCCCCCGTTCAGTCCGACCGCTG<br>CGCCTTATCCGGTAACTATCGTCTTGAGTCCAACCCGGAAAGACATGCAAAAGCA<br>CCACTGGCAGCAGCCACTGGTAATTGATTTAGAGGAGTTAGTCTTGAAGTCATGC<br>GCCGGTTAAGGCTAAACTGAAAGGACAAGTTTTGGTGACTGCGCTCCTCCAAGCC<br>AGTTACCTCGGTTCAAAGAGTTGGTAGCTCAGAGAACCTTCGAAAAACCGCCCTG<br>CAAGGCGGTTTTTTCGTTTTCAGAGCAAGAGATTACGCGCAGACCAAAACGATCT<br>CAAGAAGATCATCTTATTAATCAGATAAAATATTTCTAGATTTCAGTGCAATTTAT<br>CTCTTCAAATGTAGCACCTGAAGTCAGCCCCATACGATATAAGTTGTAATTCTCAT<br>GTTTGACAGCTTATCATCGATAAGCTTGGTACCCAATCGACATCGCATCTTTTTGT<br>ACCTATAATGTGTGGATACCCGTTTTTTTGGGCTAACAGGAGGAATTAGATCATG<br>ACGGTCAAGTACACCGATGCTCAAATTCAACGTCTTCGCGAATATGGAAACGGAA<br>CCTACGAACAAAAGGTTTTCGAGGACTTAGCATCTCGTGACGCGGCTTTCAGTAA<br>AGAGATGAGCGTGGCGTCAACTGACAATGAAAAAAAGATTAAAGGGATGATTGCA<br>AATCCATCACGTCATGGTTTAACGCAGTTAATGAATGATATTGCAGACGCATTAGT<br>GGCAGAGGGCTTTATTGAAGTCCGTACGCCGATTTTCATCTCCAAAGATGCTTTG<br>GCACGTATGACTATCACCGAAGACAAGCCCCTGTTTAAGCAAGTTTTCTGGATCG<br>ACGAAAAGCGTGCACTTCGCCCTATGTTGGCACCAAACCTGGCCTCCGTACTGCG<br>CGACTTACGCGATCACACCGACGGACCCGTGAAGATTTTCGAAATGGGATCATGT<br>TTTCGCAAGGAATCACATTCAGGGATGCATCTGGAGGAATTCACCATGTTGAACT<br>TAGTTGATATGGGACCGCGCGGCGATGCCACAGAAGTATTAAAAAACTACATCAG<br>TGTCGTAATGAAGGCTGCTGGTTTGCCTGATTATGATTTAGTACAAGAAGAAAGT<br>GATGTATACAAAGAAACAATTGATGTGGAAATCAACGGGCAAGAAGTATGCAGTG<br>CCGCAGTGGGGCCGATTCCGCTGGATGCGGCCCATGACGTGCATGAGCCTTGGT<br>CTGGTGCTGGCTTCGGGTTGGAGCGTCTTTTAACGATTCGTGAGAAATACTCAAC<br>GGTCAAGAAAGGCGGCGCTTCCATCAGTTACTTGAACGGCGCCAAGATTAATTGA<br>GTTTAAACGGTCTCCAGCTTGGCTGTTTTGGCGGATGAGAGAAGATTTTCAGCCT<br>GATACAGATTAAATCAGAACGCAGAAGCGGTCTGATAAAACAGAATTTGCCTGGC<br>GGCAGTAGCGCGGTGGTCCCACCTGACCCCATGCCGAACTCAGAAGTGAAACGC<br>CGTAGCGCCGATGGTAGTGTGGGGTCTCCCCATGCGAGAGTAGGGAACTGCCAG

GCATCAAATAAAACGAAAGGCTCAGTCGAAAGACTGGGCCTTGTTTGTGAGCTCC
CGGTCATCAATCATCCCCATAATCCTTGTTAGATTATCAATTTTAAAAAACTAACA
GTTGTCAGCCTGTCCCGCTTTAATATCATGTGAGCACCGGTTTATTGACTACCGGA
AGCAGTGTGACCGTGTGCTTCTCAAATGCCTGAGGCCAGTTTGCTCAGGCTCTCC
CCGTGGAGGTAATAATTGACGATATGATCAGTGCACGGCTAACTAAGCGGCCTGC
TGACTTTCTCGCCGATCAAAAGGCATTTTGCTATTAAGGGATTGACGAGGGCGTA
TCTGCGCAGTAAGATGCGCCCCGCATTGGGGGACGGTCCGGCGACCAGCGGGTC
TCTAAAACCTAGCCAGCGGGGTTCGACGCCCCGGTCTCTCGCCAAATTCGAAAAG
CCTGCTCAACGAGCAGGCTTTTTTGCATGCTCGAGCAGCTCAGTCATAAAAAATTT
ATTTGCTTTGTGAGCGGATAACAATTATAATAGATTCAATTGTGAGCGGATAACAA
TTTCACACAGAATTCATTAAAGAGGAGAAATTAACTATGAGTAAAGGAGAAGAAC
TTTTCACTGGAGTTGTCCCAATTCTTGTTGAATTAGATGGTGATGTTAATGGGCAC
TAGTTTTCTGTCAGTGGAGAGGGTGAAGGTGATGCAACATACGGAAAACTTACCC
TTAAATTTATTTGCACTACTGGAAAACTACCTGTTCCATGGCCAACACTTGTCACT
ACTTTGACCTGGGGTGTTCAATGCTTTGCGAGATACCCAGATCATATGAAACAGC
ATGACTTTTTCAAGAGTGCCATGCCCGAAGGTTATGTACAGGAAAGAACTATATTT
TTCAAAGATGACGGGAACTACAAGACACGTGCTGAAGTCAAGTTTGAAGGTGATA
CCCTTGTTAATAGAATCGAGTTAAAAGGTATTGATTTTAAAGAAGATGGAAACATT
CTTGGACACAAATTGGAATACAACTATATCTCAGACAATGTATACATCACGGCAG
ACAAACAAAAGAATGGAATCAAAGCGAACTTCAAAATTAGACACAACATTGAAGA
TGGAGGTGTTCAACTAGCAGACCATTATCAACAAAATACTCCAATTGGCGATGGC
CCTGTCCTTTTACCAGACAACCATTACCTGTCCACCCAATCTAAGCTCTCGAAAGA
TCCCAACGAAAAGAGAGACCACATGGTCCTTCTTGAGTTTGTAACAGCTGCTGGG
ATTACACATGGCATGGATGAACTATACAAATAAGACTCCTGTTGATAGATCCAGTA
ATGACCTCAGAACTCCATCTGGATTTGTTCAGAACGCTCGGTTGCCGCCGGGCGT
TTTTTATTGGTGAGAATCCAAGCTAGCTTGGCGAGATTTTCAGGAGCTAAGGAAG
CTAAATTTTTTTAAGGCAGTTATTGGTGCCCTTAAACGCCTGGGGTAATGACTCTC
TAGCTTGAGGCATCAAATAAAACGAAAGGCTCAGTCGAAAGACTGGGCCTTTCGT
TTTATCTGTTGTTTGTCGGTGAACGCTCTCCTGAGTAGGACAAATCCGCCCTCTAG
ACTGGGTTGAAGGCTCTCAAGGGCATCGGTCGAGATCCCGGTGCCTAATGAGTGA
GCTAACTTACATTAATTGCGTTGCGCTCACTGCCCGCTTTCCAGTCGGGAAACCT
GTCGTGCCAGCTGCATTAATGAATCGGCCAACGCGCGGGGAGAGGCGGTTTGCG
TATTGGGCGCCAGGGTGGTTTTTCTTTTCACCAGTGAGACGGGCAACAGCTGATT
GCCCTTCACCGCCTGGCCCTGAGAGAGTTGCAGCAAGCGGTCCACGCTGGTTTGC
CCCAGCAGGCGAAAATCCTGTTTGATGGTGGTTAACGGCGGGATATAACATGAGC
TGTCTTCGGTATCGTCGTATCCCACTACCGAGATATCCGCACCAACGCGCAGCCC
GGACTCGGTAATGGCGCGCATTGCGCCCAGCGCCATCTGATCGTTGGCAACCAGC
ATCGCAGTGGGAACGATGCCCTCATTCAGCATTTGCATGGTTTGTTGAAAACCGG
ACATGGCACTCCAGTCGCCTTCCCGTTCCGCTATCGGCTGAATTTGATTGCGAGT
GAGATATTTATGCCAGCCAGCCAGACGCAGACGCGCCGAGACAGAACTTAATGGG
CCCGCTAACAGCGCGATTTGCTGGTGACCCAATGCGACCAGATGCTCCACGCCCA
GTCGCGTACCGTCTTCATGGGAGAAAATAATACTGTTGATGGGTGTCTGGTCAGA
GACATCAAGAAATAACGCCGGAACATTAGTGCAGGCAGCTTCCACAGCAATGGCA
TCCTGGTCATCCAGCGGATAGTTAATGATCAGCCCACTGACGCGTTGCGCGAGAA
GATTGTGCACCGCCGCTTTACAGGCTTCGACGCCGCTTCGTTCTACCATCGACAC
CACCACGCTGGCACCCAGTTGATCGGCGCGAGATTTAATCGCCGCGACAATTTGC
GACGGCGCGTGCAGGGCCAGACTGGAGGTGGCAACGCCAATCAGCAACGACTGT
TTGCCCGCCAGTTGTTGTGCCACGCGGTTGGGAATGTAATTCAGCTCCGCCATCG
CCGCTTCCACTTTTTCCCGCGTTTTCGCAGAAACGTGGCTGGCCTGGTTCACCAC
GCGGGAAACGGTCTGATAAGAGACACCGGCATACTCTGCGACATCGTATAACGTT
ACTGGTTTCACATTCACCACCCTGAATTGACTCTCTTCCGGGCGCTATCATGCCAT
ACCGCGAAAGGTTTTGCACCATTCGATGGTGTCCAATAACTGCCTTAAAAAAATTA
CGCCCCGCCCTGCCACTCATCGCAGTACTGTTGTAATTCATTAAGCATTCTGCCGA
CATGGAAGCCATCACAGACGGCATGATGAACCTGAATCGCCAGCGGCATCAGCAC
CTTGTCGCCTTGCGTATAATATTTGCCCATGGTGAAAACGGGGGCGAAGAAGTTG

**TCCATATTGGCCACGTTTAAATCAAAACTGGTGAAACTCACCCAGGGATTGGCTG
AGACGAAAAACATATTCTCAATAAACCCTTTAGGGAAATAGGCCAGGTTTTCACC
GTAACACGCCACATCTTGCGAATATATGTGTAGAAACTGCCGGAAATCGTCGTGG
TATTCACTCCAGAGCGATGAAAACGTTTCAGTTTGCTCATGGAAAACGGTGTAAC
AAGGGTGAACACTATCCCATATCACCAGCTCACCGTCTTTCATTGCCATACGGAAT
TCCGGATGAGCATTCATCAGGCGGGCAAGAATGTGAATAAAGGCCGGATAAAACT
TGTGCTTATTTTTCTTTACGGTCTTTAAAAAGGCCGTAATATCCAGCTGAACGGTC
TGGTTATAGGTACATTGAGCAACTGACTGAAATGCCTCAAAATGTTCTTTACGATG
CCATTGGGATATATCAACGGTGGTATATCCAGTGATTTTTTTCTCCATTTTAGCTT
CCTTAGC**

pFI8 – aFAB120 promoter

**TCCTGAAAATCTCGATAACTCAAAAAATACGCCCGGTAGTGATCTTATTTCATTAT
GGTGAAAGTTGGAACCTCTTACGTGCCGATCAACGTCTCATTTTCGCCAAAAGTT
GGCCCAGGGCTTCCCGGTATCAACAGGGACACCAGGATTTATTTATTCTGCGAAG
TGATCTTCCGTCACAGGTATTTATTCGGCGCAAAGTGCGTCGGGTGATGCTGCCA
ACTTACTGATTTAGTGTATGATGGTGTTTTTGAGGTGCTCCAGTGGCTTCTGTTTC
TATCAGCTGTCCCTCCTGTTCAGCTACTGACGGGGTGGTGCGTAACGGCAAAAGC
ACCGCCGGACATCAGCGCTAGCGGAGTGTATACTGGCTTACTATGTTGGCACTGA
TGAGGGTGTCAGTGAAGTGCTTCATGTGGCAGGAGAAAAAAGGCTGCACCGGTG
CGTCAGCAGAATATGTGATACAGGATATATTCCGCTTCCTCGCTCACTGACTCGCT
ACGCTCGGTCGTTCGACTGCGGCGAGCGGAAATGGCTTACGAACGGGGCGGAGA
TTTCCTGGAAGATGCCAGGAAGATACTTAACAGGGAAGTGAGAGGGCCGCGGCA
AAGCCGTTTTTCCATAGGCTCCGCCCCCCTGACAAGCATCACGAAATCTGACGCT
CAAATCAGTGGTGGCGAAACCCGACAGGACTATAAAGATACCAGGCGTTTCCCCC
TGGCGGCTCCCTCGTGCGCTCTCCTGTTCCTGCCTTTCGGTTTACCGGTGTCATTC
CGCTGTTATGGCCGCGTTTGTCTCATTCCACGCCTGACACTCAGTTCCGGGTAGG
CAGTTCGCTCCAAGCTGGACTGTATGCACGAACCCCCCGTTCAGTCCGACCGCTG
CGCCTTATCCGGTAACTATCGTCTTGAGTCCAACCCGGAAAGACATGCAAAAGCA
CCACTGGCAGCAGCCACTGGTAATTGATTTAGAGGAGTTAGTCTTGAAGTCATGC
GCCGGTTAAGGCTAAACTGAAAGGACAAGTTTTGGTGACTGCGCTCCTCCAAGCC
AGTTACCTCGGTTCAAAGAGTTGGTAGCTCAGAGAACCTTCGAAAAACCGCCCTG
CAAGGCGGTTTTTTCGTTTTCAGAGCAAGAGATTACGCGCAGACCAAAACGATCT
CAAGAAGATCATCTTATTAATCAGATAAAATATTTCTAGATTTCAGTGCAATTTAT
CTCTTCAAATGTAGCACCTGAAGTCAGCCCCATACGATATAAGTTGTAATTCTCAT
GTTTGACAGCTTATCATCGATAAGCTTGGTACCCAATCGACATCGCATCTTTTTGT
ACCTATAATGTGTGGATACCCGTTTTTTTGGGCTAACAGGAGGAATTAGATCATG
ACGGTCAAGTACACCGATGCTCAAATTCAACGTCTTCGCGAATATGGAAACGGAA
CCTACGAACAAAAGGTTTTCGAGGACTTAGCATCTCGTGACGCGGCTTTCAGTAA
AGAGATGAGCGTGGCGTCAACTGACAATGAAAAAAAGATTAAAGGGATGATTGCA
AATCCATCACGTCATGGTTTAACGCAGTTAATGAATGATATTGCAGACGCATTAGT
GGCAGAGGGCTTTATTGAAGTCCGTACGCCGATTTTCATCTCCAAAGATGCTTTG
GCACGTATGACTATCACCGAAGACAAGCCCCTGTTTAAGCAAGTTTTCTGGATCG
ACGAAAAGCGTGCACTTCGCCCTATGTTGGCACCAAACCTGGCCTCCGTACTGCG
CGACTTACGCGATCACACCGACGGACCCGTGAAGATTTTCGAAATGGGATCATGT
TTTCGCAAGGAATCACATTCAGGGATGCATCTGGAGGAATTCACCATGTTGAACT
TAGTTGATATGGGACCGCGCGGCGATGCCACAGAAGTATTAAAAAACTACATCAG
TGTCGTAATGAAGGCTGCTGGTTTGCCTGATTATGATTTAGTACAAGAAGAAAGT
GATGTATACAAAGAAACAATTGATGTGGAAATCAACGGGCAAGAAGTATGCAGTG
CCGCAGTGGGGCCGATTCCGCTGGATGCGGCCCATGACGTGCATGAGCCTTGGT
CTGGTGCTGGCTTCGGGTTGGAGCGTCTTTTAACGATTCGTGAGAAATACTCAAC
GGTCAAGAAAGGCGGCGCTTCCATCAGTTACTTGAACGGCGCCAAGATTAATTGA
GTTTAAACGGTCTCCAGCTTGGCTGTTTTGGCGGATGAGAGAAGATTTTCAGCCT
GATACAGATTAAATCAGAACGCAGAAGCGGTCTGATAAAACAGAATTTGCCTGGC**

**GGCAGTAGCGCGGTGGTCCCACCTGACCCCATGCCGAACTCAGAAGTGAAACGC
CGTAGCGCCGATGGTAGTGTGGGGTCTCCCCATGCGAGAGTAGGGAACTGCCAG
GCATCAAATAAAACGAAAGGCTCAGTCGAAAGACTGGGCCTTGTTTGTGAGCTCC
CGGTCATCAATCATCCCCATAATCCTTGTTAGATTATCAATTTTAAAAAACTAACA
GTTGTCAGCCTGTCCCGCTTTAATATCATGTGAGCACCGGTTTATTGACTACCGGA
AGCAGTGTGACCGTGTGCTTCTCAAATGCCTGAGGCCAGTTTGCTCAGGCTCTCC
CCGTGGAGGTAATAATTGACGATATGATCAGTGCACGGCTAACTAAGCGGCCTGC
TGACTTTCTCGCCGATCAAAAGGCATTTTGCTATTAAGGGATTGACGAGGGCGTA
TCTGCGCAGTAAGATGCGCCCCGCATTGGGGGACGGTCCGGCGACCAGCGGGTC
TCTAAAACCTAGCCAGCGGGGTTCGACGCCCCGGTCTCTCGCCAAATTCGAAAAG
CCTGCTCAACGAGCAGGCTTTTTTGCATGCTCGAGCAGCTCAGTCATAAAAAATTT
ATTTGCTTTGTGAGCGGATAACAATTATAATAGATTCAATTGTGAGCGGATAACAA
TTTCACACAGAATTCATTAAAGAGGAGAAATTAACTATGAGTAAAGGAGAAGAAC
TTTTCACTGGAGTTGTCCCAATTCTTGTTGAATTAGATGGTGATGTTAATGGGCAC
AAATTTTCTGTCAGTGGAGAGGGTGAAGGTGATGCAACATACGGAAAACTTACCC
TTTAGTTTATTTGCACTACTGGAAAACTACCTGTTCCATGGCCAACACTTGTCACT
ACTTTGACCTGGGGTGTTCAATGCTTTGCGAGATACCCAGATCATATGAAACAGC
ATGACTTTTTCAAGAGTGCCATGCCCGAAGGTTATGTACAGGAAAGAACTATATTT
TTCAAAGATGACGGGAACTACAAGACACGTGCTGAAGTCAAGTTTGAAGGTGATA
CCCTTGTTAATAGAATCGAGTTAAAAGGTATTGATTTTAAAGAAGATGGAAACATT
CTTGGACACAAATTGGAATACAACTATATCTCAGACAATGTATACATCACGGCAG
ACAAACAAAAGAATGGAATCAAAGCGAACTTCAAAATTAGACACAACATTGAAGA
TGGAGGTGTTCAACTAGCAGACCATTATCAACAAAATACTCCAATTGGCGATGGC
CCTGTCCTTTTACCAGACAACCATTACCTGTCCACCCAATCTAAGCTCTCGAAAGA
TCCCAACGAAAAGAGAGACCACATGGTCCTTCTTGAGTTTGTAACAGCTGCTGGG
ATTACACATGGCATGGATGAACTATACAAATAAGACTCCTGTTGATAGATCCAGTA
ATGACCTCAGAACTCCATCTGGATTTGTTCAGAACGCTCGGTTGCCGCCGGGCGT
TTTTTATTGGTGAGAATCCAAGCTAGCTTGGCGAGATTTTCAGGAGCTAAGGAAG
CTAAATTTTTTTAAGGCAGTTATTGGTGCCCTTAAACGCCTGGGGTAATGACTCTC
TAGCTTGAGGCATCAAATAAAACGAAAGGCTCAGTCGAAAGACTGGGCCTTTCGT
TTTATCTGTTGTTTGTCGGTGAACGCTCTCCTGAGTAGGACAAATCCGCCCTCTAG
ACTGGGTTGAAGGCTCTCAAGGGCATCGGTCGAGATCCCGGTGCCTAATGAGTGA
GCTAACTTACATTAATTGCGTTGCGCTCACTGCCCGCTTTCCAGTCGGGAAACCT
GTCGTGCCAGCTGCATTAATGAATCGGCCAACGCGCGGGGAGAGGCGGTTTGCG
TATTGGGCGCCAGGGTGGTTTTTCTTTTCACCAGTGAGACGGGCAACAGCTGATT
GCCCTTCACCGCCTGGCCCTGAGAGAGTTGCAGCAAGCGGTCCACGCTGGTTTGC
CCCAGCAGGCGAAAATCCTGTTTGATGGTGGTTAACGGCGGGATATAACATGAGC
TGTCTTCGGTATCGTCGTATCCCACTACCGAGATATCCGCACCAACGCGCAGCCC
GGACTCGGTAATGGCGCGCATTGCGCCCAGCGCCATCTGATCGTTGGCAACCAGC
ATCGCAGTGGGAACGATGCCCTCATTCAGCATTTGCATGGTTTGTTGAAAACCGG
ACATGGCACTCCAGTCGCCTTCCCGTTCCGCTATCGGCTGAATTTGATTGCGAGT
GAGATATTTATGCCAGCCAGCCAGACGCAGACGCGCCGAGACAGAACTTAATGGG
CCCGCTAACAGCGCGATTTGCTGGTGACCCAATGCGACCAGATGCTCCACGCCCA
GTCGCGTACCGTCTTCATGGGAGAAAATAATACTGTTGATGGGTGTCTGGTCAGA
GACATCAAGAAATAACGCCGGAACATTAGTGCAGGCAGCTTCCACAGCAATGGCA
TCCTGGTCATCCAGCGGATAGTTAATGATCAGCCCACTGACGCGTTGCGCGAGAA
GATTGTGCACCGCCGCTTTACAGGCTTCGACGCCGCTTCGTTCTACCATCGACAC
CACCACGCTGGCACCCAGTTGATCGGCGCGAGATTTAATCGCCGCGACAATTTGC
GACGGCGCGTGCAGGGCCAGACTGGAGGTGGCAACGCCAATCAGCAACGACTGT
TTGCCCGCCAGTTGTTGTGCCACGCGGTTGGGAATGTAATTCAGCTCCGCCATCG
CCGCTTCCACTTTTTCCCGCGTTTTCGCAGAAACGTGGCTGGCCTGGTTCACCAC
GCGGGAAACGGTCTGATAAGAGACACCGGCATACTCTGCGACATCGTATAACGTT
ACTGGTTTCACATTCACCACCCTGAATTGACTCTCTTCCGGGCGCTATCATGCCAT
ACCGCGAAAGGTTTTGCACCATTCGATGGTGTCCAATAACTGCCTTAAAAAAATTA
CGCCCCGCCCTGCCACTCATCGCAGTACTGTTGTAATTCATTAAGCATTCTGCCGA**

**CATGGAAGCCATCACAGACGGCATGATGAACCTGAATCGCCAGCGGCATCAGCAC**<br>**CTTGTCGCCTTGCGTATAATATTTGCCCATGGTGAAAACGGGGGCGAAGAAGTTG**<br>**TCCATATTGGCCACGTTTAAATCAAAACTGGTGAAACTCACCCAGGGATTGGCTG**<br>**AGACGAAAAACATATTCTCAATAAACCCTTTAGGGAAATAGGCCAGGTTTTCACC**<br>**GTAACACGCCACATCTTGCGAATATATGTGTAGAAACTGCCGGAAATCGTCGTGG**<br>**TATTCACTCCAGAGCGATGAAAACGTTTCAGTTTGCTCATGGAAAACGGTGTAAC**<br>**AAGGGTGAACACTATCCCATATCACCAGCTCACCGTCTTTCATTGCCATACGGAAT**<br>**TCCGGATGAGCATTCATCAGGCGGGCAAGAATGTGAATAAAGGCCGGATAAAACT**<br>**TGTGCTTATTTTTCTTTACGGTCTTTAAAAAGGCCGTAATATCCAGCTGAACGGTC**<br>**TGGTTATAGGTACATTGAGCAACTGACTGAAATGCCTCAAAATGTTCTTTACGATG**<br>**CCATTGGGATATATCAACGGTGGTATATCCAGTGATTTTTTTCTCCATTTTAGCTT**<br>**CCTTAGC**

**pFI4_UAG_Halo and pFI4_AAA_Halo protein**
**TCCTGAAAATCTCGATAACTCAAAAAATACGCCCGGTAGTGATCTTATTTCATTAT**<br>**GGTGAAAGTTGGAACCTCTTACGTGCCGATCAACGTCTCATTTTCGCCAAAAGTT**<br>**GGCCCAGGGCTTCCCGGTATCAACAGGGACACCAGGATTTATTTATTCTGCGAAG**<br>**TGATCTTCCGTCACAGGTATTTATTCGGCGCAAAGTGCGTCGGGTGATGCTGCCA**<br>**ACTTACTGATTTAGTGTATGATGGTGTTTTTGAGGTGCTCCAGTGGCTTCTGTTTC**<br>**TATCAGCTGTCCCTCCTGTTCAGCTACTGACGGGGTGGTGCGTAACGGCAAAAGC**<br>**ACCGCCGGACATCAGCGCTAGCGGAGTGTATACTGGCTTACTATGTTGGCACTGA**<br>**TGAGGGTGTCAGTGAAGTGCTTCATGTGGCAGGAGAAAAAAGGCTGCACCGGTG**<br>**CGTCAGCAGAATATGTGATACAGGATATATTCCGCTTCCTCGCTCACTGACTCGCT**<br>**ACGCTCGGTCGTTCGACTGCGGCGAGCGGAAATGGCTTACGAACGGGGCGGAGA**<br>**TTTCCTGGAAGATGCCAGGAAGATACTTAACAGGGAAGTGAGAGGGCCGCGGCA**<br>**AAGCCGTTTTTCCATAGGCTCCGCCCCCCTGACAAGCATCACGAAATCTGACGCT**<br>**CAAATCAGTGGTGGCGAAACCCGACAGGACTATAAAGATACCAGGCGTTTCCCCC**<br>**TGGCGGCTCCCTCGTGCGCTCTCCTGTTCCTGCCTTTCGGTTTACCGGTGTCATTC**<br>**CGCTGTTATGGCCGCGTTTGTCTCATTCCACGCCTGACACTCAGTTCCGGGTAGG**<br>**CAGTTCGCTCCAAGCTGGACTGTATGCACGAACCCCCCGTTCAGTCCGACCGCTG**<br>**CGCCTTATCCGGTAACTATCGTCTTGAGTCCAACCCGGAAAGACATGCAAAAGCA**<br>**CCACTGGCAGCAGCCACTGGTAATTGATTTAGAGGAGTTAGTCTTGAAGTCATGC**<br>**GCCGGTTAAGGCTAAACTGAAAGGACAAGTTTTGGTGACTGCGCTCCTCCAAGCC**<br>**AGTTACCTCGGTTCAAAGAGTTGGTAGCTCAGAGAACCTTCGAAAAACCGCCCTG**<br>**CAAGGCGGTTTTTTCGTTTTCAGAGCAAGAGATTACGCGCAGACCAAAACGATCT**<br>**CAAGAAGATCATCTTATTAATCAGATAAAATATTTCTAGATTTCAGTGCAATTTAT**<br>**CTCTTCAAATGTAGCACCTGAAGTCAGCCCCATACGATATAAGTTGTAATTCTCAT**<br>**GTTTGACAGCTTATCATCGATAAGCTTGGTACCCAATCGACATCGCATCTTTTTGT**<br>**ACCTATAATGTGTGGATACCCGTTTTTTTGGGCTAACAGGAGGAATTAGATCATG**<br>**ACGGTCAAGTACACCGATGCTCAAATTCAACGTCTTCGCGAATATGGAAACGGAA**<br>**CCTACGAACAAAAGGTTTTCGAGGACTTAGCATCTCGTGACGCGGCTTTCAGTAA**<br>**AGAGATGAGCGTGGCGTCAACTGACAATGAAAAAAAGATTAAAGGGATGATTGCA**<br>**AATCCATCACGTCATGGTTTAACGCAGTTAATGAATGATATTGCAGACGCATTAGT**<br>**GGCAGAGGGCTTTATTGAAGTCCGTACGCCGATTTTCATCTCCAAAGATGCTTTG**<br>**GCACGTATGACTATCACCGAAGACAAGCCCCTGTTTAAGCAAGTTTTCTGGATCG**<br>**ACGAAAAGCGTGCACTTCGCCCTATGTTGGCACCAAACCTGGCCTCCGTACTGCG**<br>**CGACTTACGCGATCACACCGACGGACCCGTGAAGATTTTCGAAATGGGATCATGT**<br>**TTTCGCAAGGAATCACATTCAGGGATGCATCTGGAGGAATTCACCATGTTGAACT**<br>**TAGTTGATATGGGACCGCGCGGCGATGCCACAGAAGTATTAAAAAACTACATCAG**<br>**TGTCGTAATGAAGGCTGCTGGTTTGCCTGATTATGATTTAGTACAAGAAGAAAGT**<br>**GATGTATACAAAGAAACAATTGATGTGGAAATCAACGGGCAAGAAGTATGCAGTG**<br>**CCGCAGTGGGGCCGATTCCGCTGGATGCGGCCCATGACGTGCATGAGCCTTGGT**

CTGGTGCTGGCTTCGGGTTGGAGCGTCTTTTAACGATTCGTGAGAAATACTCAAC
GGTCAAGAAAGGCGGCGCTTCCATCAGTTACTTGAACGGCGCCAAGATTAATTGA
GTTTAAACGGTCTCCAGCTTGGCTGTTTTGGCGGATGAGAGAAGATTTTCAGCCT
GATACAGATTAAATCAGAACGCAGAAGCGGTCTGATAAAACAGAATTTGCCTGGC
GGCAGTAGCGCGGTGGTCCCACCTGACCCCATGCCGAACTCAGAAGTGAAACGC
CGTAGCGCCGATGGTAGTGTGGGGTCTCCCCATGCGAGAGTAGGGAACTGCCAG
GCATCAAATAAAACGAAAGGCTCAGTCGAAAGACTGGGCCTTGTTTGTGAGCTCC
CGGTCATCAATCATCCCCATAATCCTTGTTAGATTATCAATTTTAAAAAACTAACA
GTTGTCAGCCTGTCCCGCTTTAATATCATGTGAGCACCGGTTTATTGACTACCGGA
AGCAGTGTGACCGTGTGCTTCTCAAATGCCTGAGGCCAGTTTGCTCAGGCTCTCC
CCGTGGAGGTAATAATTGACGATATGATCAGTGCACGGCTAACTAAGCGGCCTGC
TGACTTTCTCGCCGATCAAAAGGCATTTTGCTATTAAGGGATTGACGAGGGCGTA
TCTGCGCAGTAAGATGCGCCCCGCATTGGGGGACGGTCCGGCGACCAGCGGGTC
TCTAAAACCTAGCCAGCGGGGTTCGACGCCCCGGTCTCTCGCCAAATTCGAAAAG
CCTGCTCAACGAGCAGGCTTTTTTGCATGCTCGAGCAGCTCAGTCATAAAAAATTT
ATTTGCTTTGTGAGCGGATAACAATTATAATAGATTCAATTGTGAGCGGATAACAA
TTTCACACAGAATTCATTAAAGAGGAGAAATTAACTATGGGCCATCATTAG/AAAA
AGAATGGCGGTGCGAGCGCAGAAATCGGTACTGGCTTTCCATTCGACCCCCATTA
TGTGGAAGTCCTGGGCGAGCGCATGCACTACGTCGATGTTGGTCCGCGCGATGG
CACCCCTGTGCTGTTCCTGCACGGTAACCCGACCTCCTCCTACGTGTGGCGCAAC
ATCATCCCGCATGTTGCACCGACCCATCGCTGCATTGCTCCAGACCTGATCGGTA
TGGGCAAATCCGACAAACCAGACCTGGGTTATTTCTTCGACGACCACGTCCGCTT
CATGGATGCCTTCATCGAAGCCCTGGGTCTGGAAGAGGTCGTCCTGGTCATTCAC
GACTGGGGCTCCGCTCTGGGTTTCCACTGGGCCAAGCGCAATCCAGAGCGCGTCA
AAGGTATTGCATTTATGGAGTTCATCCGCCCTATCCCGACCTGGGACGAATGGCC
AGAATTTGCCCGCGAGACCTTCCAGGCCTTCCGCACCACCGACGTCGGCCGCAAG
CTGATCATCGATCAGAACGTTTTTATCGAGGGTACGCTGCCGATGGGTGTCGTCC
GCCCGCTGACTGAAGTCGAGATGGACCATTACCGCGAGCCGTTCCTGAATCCTGT
TGACCGCGAGCCACTGTGGCGCTTCCCAAACGAGCTGCCAATCGCCGGTGAGCCA
GCGAACATCGTCGCGCTGGTCGAAGAATACATGGACTGGCTGCACCAGTCCCCTG
TCCCGAAGCTGCTGTTCTGGGGCACCCCAGGCGTTCTGATCCCACCGGCCGAAGC
CGCTCGCCTGGCCAAAAGCCTGCCTAACTGCAAGGCTGTGGACATCGGCCCGGGT
CTGAATCTGCTGCAAGAAGACAACCCGGACCTGATCGGCAGCGAGATCGCGCGCT
GGCTGTCGACGCTCGAGATTTCCGGCTGAGACTCCTGTTGATAGATCCAGTAATG
ACCTCAGAACTCCATCTGGATTTGTTCAGAACGCTCGGTTGCCGCCGGGCGTTTT
TTATTGGTGAGAATCCAAGCTAGCTTGGCGAGATTTTCAGGAGCTAAGGAAGCTA
AATTTTTTTAAGGCAGTTATTGGTGCCCTTAAACGCCTGGGGTAATGACTCTCTAG
CTTGAGGCATCAAATAAAACGAAAGGCTCAGTCGAAAGACTGGGCCTTTCGTTTT
ATCTGTTGTTTGTCGGTGAACGCTCTCCTGAGTAGGACAAATCCGCCCTCTAGAC
TGGGTTGAAGGCTCTCAAGGGCATCGGTCGAGATCCCGGTGCCTAATGAGTGAGC
TAACTTACATTAATTGCGTTGCGCTCACTGCCCGCTTTCCAGTCGGGAAACCTGTC
GTGCCAGCTGCATTAATGAATCGGCCAACGCGCGGGGAGAGGCGGTTTGCGTATT
GGGCGCCAGGGTGGTTTTTCTTTTCACCAGTGAGACGGGCAACAGCTGATTGCCC
TTCACCGCCTGGCCCTGAGAGAGTTGCAGCAAGCGGTCCACGCTGGTTTGCCCCA
GCAGGCGAAAATCCTGTTTGATGGTGGTTAACGGCGGGATATAACATGAGCTGTC
TTCGGTATCGTCGTATCCCACTACCGAGATATCCGCACCAACGCGCAGCCCGGAC
TCGGTAATGGCGCGCATTGCGCCCAGCGCCATCTGATCGTTGGCAACCAGCATCG
CAGTGGGAACGATGCCCTCATTCAGCATTTGCATGGTTTGTTGAAAACCGGACAT
GGCACTCCAGTCGCCTTCCCGTTCCGCTATCGGCTGAATTTGATTGCGAGTGAGA
TATTTATGCCAGCCAGCCAGACGCAGACGCGCCGAGACAGAACTTAATGGGCCCG
CTAACAGCGCGATTTGCTGGTGACCCAATGCGACCAGATGCTCCACGCCCAGTCG
CGTACCGTCTTCATGGGAGAAAATAATACTGTTGATGGGTGTCTGGTCAGAGACA
TCAAGAAATAACGCCGGAACATTAGTGCAGGCAGCTTCCACAGCAATGGCATCCT
GGTCATCCAGCGGATAGTTAATGATCAGCCCACTGACGCGTTGCGCGAGAAGATT
GTGCACCGCCGCTTTACAGGCTTCGACGCCGCTTCGTTCTACCATCGACACCACC

**ACGCTGGCACCCAGTTGATCGGCGCGAGATTTAATCGCCGCGACAATTTGCGACG
GCGCGTGCAGGGCCAGACTGGAGGTGGCAACGCCAATCAGCAACGACTGTTTGC
CCGCCAGTTGTTGTGCCACGCGGTTGGGAATGTAATTCAGCTCCGCCATCGCCGC
TTCCACTTTTTCCCGCGTTTTCGCAGAAACGTGGCTGGCCTGGTTCACCACGCGG
GAAACGGTCTGATAAGAGACACCGGCATACTCTGCGACATCGTATAACGTTACTG
GTTTCACATTCACCACCCTGAATTGACTCTCTTCCGGGCGCTATCATGCCATACCG
CGAAAGGTTTTGCACCATTCGATGGTGTCCAATAACTGCCTTAAAAAATTACGCC
CCGCCCTGCCACTCATCGCAGTACTGTTGTAATTCATTAAGCATTCTGCCGACATG
GAAGCCATCACAGACGGCATGATGAACCTGAATCGCCAGCGGCATCAGCACCTTG
TCGCCTTGCGTATAATATTTGCCCATGGTGAAAACGGGGGCGAAGAAGTTGTCCA
TATTGGCCACGTTTAAATCAAAACTGGTGAAACTCACCCAGGGATTGGCTGAGAC
GAAAAACATATTCTCAATAAACCCTTTAGGGAAATAGGCCAGGTTTTCACCGTAAC
ACGCCACATCTTGCGAATATATGTGTAGAAACTGCCGGAAATCGTCGTGGTATTC
ACTCCAGAGCGATGAAAACGTTTCAGTTTGCTCATGGAAAACGGTGTAACAAGGG
TGAACACTATCCCATATCACCAGCTCACCGTCTTTCATTGCCATACGGAATTCCGG
ATGAGCATTCATCAGGCGGGCAAGAATGTGAATAAAGGCCGGATAAAACTTGTGC
TTATTTTTCTTTACGGTCTTTAAAAAGGCCGTAATATCCAGCTGAACGGTCTGGTT
ATAGGTACATTGAGCAACTGACTGAAATGCCTCAAAATGTTCTTTACGATGCCATT
GGGATATATCAACGGTGGTATATCCAGTGATTTTTTTCTCCATTTTAGCTTCCTTA
GC**

pFI4_LacY_UAG_HaloTag

**TCCTGAAAATCTCGATAACTCAAAAAATACGCCCGGTAGTGATCTTATTTCATTAT
GGTGAAAGTTGGAACCTCTTACGTGCCGATCAACGTCTCATTTTCGCCAAAAGTT
GGCCCAGGGCTTCCCGGTATCAACAGGGACACCAGGATTTATTTATTCTGCGAAG
TGATCTTCCGTCACAGGTATTTATTCGGCGCAAAGTGCGTCGGGTGATGCTGCCA
ACTTACTGATTTAGTGTATGATGGTGTTTTTGAGGTGCTCCAGTGGCTTCTGTTTC
TATCAGCTGTCCCTCCTGTTCAGCTACTGACGGGGTGGTGCGTAACGGCAAAAGC
ACCGCCGGACATCAGCGCTAGCGGAGTGTATACTGGCTTACTATGTTGGCACTGA
TGAGGGTGTCAGTGAAGTGCTTCATGTGGCAGGAGAAAAAAGGCTGCACCGGTG
CGTCAGCAGAATATGTGATACAGGATATATTCCGCTTCCTCGCTCACTGACTCGCT
ACGCTCGGTCGTTCGACTGCGGCGAGCGGAAATGGCTTACGAACGGGGCGGAGA
TTTCCTGGAAGATGCCAGGAAGATACTTAACAGGGAAGTGAGAGGGCCGCGGCA
AAGCCGTTTTTCCATAGGCTCCGCCCCCCTGACAAGCATCACGAAATCTGACGCT
CAAATCAGTGGTGGCGAAACCCGACAGGACTATAAAGATACCAGGCGTTTCCCCC
TGGCGGCTCCCTCGTGCGCTCTCCTGTTCCTGCCTTTCGGTTTACCGGTGTCATTC
CGCTGTTATGGCCGCGTTTGTCTCATTCCACGCCTGACACTCAGTTCCGGGTAGG
CAGTTCGCTCCAAGCTGGACTGTATGCACGAACCCCCCGTTCAGTCCGACCGCTG
CGCCTTATCCGGTAACTATCGTCTTGAGTCCAACCCGGAAAGACATGCAAAAGCA
CCACTGGCAGCAGCCACTGGTAATTGATTTAGAGGAGTTAGTCTTGAAGTCATGC
GCCGGTTAAGGCTAAACTGAAAGGACAAGTTTTGGTGACTGCGCTCCTCCAAGCC
AGTTACCTCGGTTCAAAGAGTTGGTAGCTCAGAGAACCTTCGAAAAACCGCCCTG
CAAGGCGGTTTTTTCGTTTTCAGAGCAAGAGATTACGCGCAGACCAAAACGATCT
CAAGAAGATCATCTTATTAATCAGATAAAATATTTCTAGATTTCAGTGCAATTTAT
CTCTTCAAATGTAGCACCTGAAGTCAGCCCCATACGATATAAGTTGTAATTCTCAT
GTTTGACAGCTTATCATCGATAAGCTTGGTACCCAATCGACATCGCATCTTTTTGT
ACCTATAATGTGTGGATACCCGTTTTTTTGGGCTAACAGGAGGAATTAGATCATG
ACGGTCAAGTACACCGATGCTCAAATTCAACGTCTTCGCGAATATGGAAACGGAA
CCTACGAACAAAAGGTTTTCGAGGACTTAGCATCTCGTGACGCGGCTTTCAGTAA
AGAGATGAGCGTGGCGTCAACTGACAATGAAAAAAAGATTAAAGGGATGATTGCA
AATCCATCACGTCATGGTTTAACGCAGTTAATGAATGATATTGCAGACGCATTAGT
GGCAGAGGGCTTTATTGAAGTCCGTACGCCGATTTTCATCTCCAAAGATGCTTTG**

**GCACGTATGACTATCACCGAAGACAAGCCCCTGTTTAAGCAAGTTTTCTGGATCG
ACGAAAAGCGTGCACTTCGCCCTATGTTGGCACCAAACCTGGCCTCCGTACTGCG
CGACTTACGCGATCACACCGACGGACCCGTGAAGATTTTCGAAATGGGATCATGT
TTTCGCAAGGAATCACATTCAGGGATGCATCTGGAGGAATTCACCATGTTGAACT
TAGTTGATATGGGACCGCGCGGCGATGCCACAGAAGTATTAAAAAACTACATCAG
TGTCGTAATGAAGGCTGCTGGTTTGCCTGATTATGATTTAGTACAAGAAGAAAGT
GATGTATACAAAGAAACAATTGATGTGGAAATCAACGGGCAAGAAGTATGCAGTG
CCGCAGTGGGGCCGATTCCGCTGGATGCGGCCCATGACGTGCATGAGCCTTGGT
CTGGTGCTGGCTTCGGGTTGGAGCGTCTTTTAACGATTCGTGAGAAATACTCAAC
GGTCAAGAAAGGCGGCGCTTCCATCAGTTACTTGAACGGCGCCAAGATTAATTGA
GTTTAAACGGTCTCCAGCTTGGCTGTTTTGGCGGATGAGAGAAGATTTTCAGCCT
GATACAGATTAAATCAGAACGCAGAAGCGGTCTGATAAAACAGAATTTGCCTGGC
GGCAGTAGCGCGGTGGTCCCACCTGACCCCATGCCGAACTCAGAAGTGAAACGC
CGTAGCGCCGATGGTAGTGTGGGGTCTCCCCATGCGAGAGTAGGGAACTGCCAG
GCATCAAATAAAACGAAAGGCTCAGTCGAAAGACTGGGCCTTGTTTGTGAGCTCC
CGGTCATCAATCATCCCCATAATCCTTGTTAGATTATCAATTTTAAAAAACTAACA
GTTGTCAGCCTGTCCCGCTTTAATATCATGTGAGCACCGGTTTATTGACTACCGGA
AGCAGTGTGACCGTGTGCTTCTCAAATGCCTGAGGCCAGTTTGCTCAGGCTCTCC
CCGTGGAGGTAATAATTGACGATATGATCAGTGCACGGCTAACTAAGCGGCCTGC
TGACTTTCTCGCCGATCAAAAGGCATTTTGCTATTAAGGGATTGACGAGGGCGTA
TCTGCGCAGTAAGATGCGCCCCGCATTGGGGGACGGTCCGGCGACCAGCGGGTC
TCTAAAAC<u>CTA</u>GCCAGCGGGGTTCGACGCCCCGGTCTCTCGCCAAATTCGAAAAG
CCTGCTCAACGAGCAGGCTTTTTTGCATGCTCGAGCAGCTCAGTCATAAAAAATTT
ATTTGCTTTGTGAGCGGATAACAATTATAATAGATTCAATTGTGAGCGGATAACAA
TTTCACACAGAATTCATTAAAGAGGAGAAATTAACTATGTACTATTTAAAAAACAC
AAACTTTTGGATGTTCGGTTTATTCTTTTTCTTTTACTTTTTTATCATGGGAGCCTA
CTTCCCGTTTTTCCCGATTTGGCTACATGACATCAACCATATCAGCAAAAGTGATA
CGGGTATTATTTTTGCCGCTATTTCTCTGTTCTCGCTATTATTCCAACCGCTGTTT
GGTCTGCTTTCTGACAAACTCGGGCTGCGCAAATACCTGCTGTGGATTATTACCG
GCATGTTAGTGATGTTTGCGCCGTTCTTTATTTTTATCTTCGGGCCACTGTTACAA
TACAACATTTTAGTAGGATCGATTGTTGGTGGTATTTATCTAGGCTTTTGTTTTAA
CGCCGGTGCGCCAGCAGTAGAGGCATTTATTGAGAAAGTCAGCCGTCGCAGTAAT
TTCGAATTTGGTCGCGCGCGGATGTTTGGCTGTGTTGGCTGGGCGCTGTGTGCCT
CGATTGTCGGCATCATGTTCACCATCAATAATCAGTTTGTTTTCTGGCTGGGCTCT
GGCTGTGCACTCATCCTCGCCGTTTTACTCTTTTTCGCCAAAACGGATGCGCCCTC
TTCTGCCACGGTTGCCAATGCGGTAGGTGCCAACCATTCGGCATTTAGCCTTAAG
CTGGCACTGGAACTGTTCAGACAGCCAAAACTGTGGTTTTTGTCACTGTATGTTAT
TGGCGTTTCCTGCACCTACGATGTTTTTGACCAACAGTTTGCTAATTTCTTTACTT
CGTTCTTTGCTACCGGTGAACAGGGTACGCGGGTATTTGGCTACGTAACGACAAT
GGGCGAATTACTTAACGCCTCGATTATGTTCTTTGCGCCACTGATCATTAATCGCA
TCGGTGGGAAAAACGCCCTGCTGCTGGCTGGCACTATTATGTCTGTACGTATTAT
TGGCTCATCGTTCGCCACCTCAGCGCTGGAAGTGGTTATTCTGAAAACGCTGCAT
ATGTTTGAAGTACCGTTCCTGCTGGTGGGCTGCTTTAAATATATTACCAGCCAGTT
TGAAGTGCGTTTTTCAGCGACGATTTATCTGGTCTGTTTCTGCTTCTTTAAGCAAC
TGGCGATGATTTTTATGTCTGTACTGGCGGGCAATATGTATGAAAGCATCGGTTT
CCAGGGCGCTTATCTGGTGCTGGGTCTGGTGGCGCTGGGCTTCACCTTAATTTCC
GTGTTCACGCTTAGCGGCCCCGGCCCGCTTTCCCTGCTGCGTCGTCAGGTGAATG
AAGTCGCTGGTAGCGGCCAT<u>TAG</u>AAAAAGAATGGCGGTGCGAGCGCAGAAATCG
GTACTGGCTTTCCATTCGACCCCCATTATGTGGAAGTCCTGGGCGAGCGCATGCA
CTACGTCGATGTTGGTCCGCGCGATGGCACCCCTGTGCTGTTCCTGCACGGTAAC
CCGACCTCCTCCTACGTGTGGCGCAACATCATCCCGCATGTTGCACCGACCCATC
GCTGCATTGCTCCAGACCTGATCGGTATGGGCAAATCCGACAAACCAGACCTGGG
TTATTTCTTCGACGACCACGTCCGCTTCATGGATGCCTTCATCGAAGCCCTGGGTC
TGGAAGAGGTCGTCCTGGTCATTCACGACTGGGGCTCCGCTCTGGGTTTCCACTG
GGCCAAGCGCAATCCAGAGCGCGTCAAAGGTATTGCATTTATGGAGTTCATCCGC**

**CCTATCCCGACCTGGGACGAATGGCCAGAATTTGCCCGCGAGACCTTCCAGGCCT
TCCGCACCACCGACGTCGGCCGCAAGCTGATCATCGATCAGAACGTTTTTATCGA
GGGTACGCTGCCGATGGGTGTCGTCCGCCCGCTGACTGAAGTCGAGATGGACCA
TTACCGCGAGCCGTTCCTGAATCCTGTTGACCGCGAGCCACTGTGGCGCTTCCCA
AACGAGCTGCCAATCGCCGGTGAGCCAGCGAACATCGTCGCGCTGGTCGAAGAA
TACATGGACTGGCTGCACCAGTCCCCTGTCCCGAAGCTGCTGTTCTGGGGCACCC
CAGGCGTTCTGATCCCACCGGCCGAAGCCGCTCGCCTGGCCAAAAGCCTGCCTAA
CTGCAAGGCTGTGGACATCGGCCCGGGTCTGAATCTGCTGCAAGAAGACAACCCG
GACCTGATCGGCAGCGAGATCGCGCGCTGGCTGTCGACGCTCGAGATTTCCGGCT
GAGACTCCTGTTGATAGATCCAGTAATGACCTCAGAACTCCATCTGGATTTGTTCA
GAACGCTCGGTTGCCGCCGGGCGTTTTTTATTGGTGAGAATCCAAGCTAGCTTGG
CGAGATTTTCAGGAGCTAAGGAAGCTAAATTTTTTTAAGGCAGTTATTGGTGCCCT
TAAACGCCTGGGGTAATGACTCTCTAGCTTGAGGCATCAAATAAAACGAAAGGCT
CAGTCGAAAGACTGGGCCTTTCGTTTTATCTGTTGTTTGTCGGTGAACGCTCTCCT
GAGTAGGACAAATCCGCCCTCTAGACTGGGTTGAAGGCTCTCAAGGGCATCGGTC
GAGATCCCGGTGCCTAATGAGTGAGCTAACTTACATTAATTGCGTTGCGCTCACT
GCCCGCTTTCCAGTCGGGAAACCTGTCGTGCCAGCTGCATTAATGAATCGGCCAA
CGCGCGGGGAGAGGCGGTTTGCGTATTGGGCGCCAGGGTGGTTTTTCTTTTCACC
AGTGAGACGGGCAACAGCTGATTGCCCTTCACCGCCTGGCCCTGAGAGAGTTGCA
GCAAGCGGTCCACGCTGGTTTGCCCCAGCAGGCGAAAATCCTGTTTGATGGTGGT
TAACGGCGGGATATAACATGAGCTGTCTTCGGTATCGTCGTATCCCACTACCGAG
ATATCCGCACCAACGCGCAGCCCGGACTCGGTAATGGCGCGCATTGCGCCCAGC
GCCATCTGATCGTTGGCAACCAGCATCGCAGTGGGAACGATGCCCTCATTCAGCA
TTTGCATGGTTTGTTGAAAACCGGACATGGCACTCCAGTCGCCTTCCCGTTCCGC
TATCGGCTGAATTTGATTGCGAGTGAGATATTTATGCCAGCCAGCCAGACGCAGA
CGCGCCGAGACAGAACTTAATGGGCCCGCTAACAGCGCGATTTGCTGGTGACCCA
ATGCGACCAGATGCTCCACGCCCAGTCGCGTACCGTCTTCATGGGAGAAAATAAT
ACTGTTGATGGGTGTCTGGTCAGAGACATCAAGAAATAACGCCGGAACATTAGTG
CAGGCAGCTTCCACAGCAATGGCATCCTGGTCATCCAGCGGATAGTTAATGATCA
GCCCACTGACGCGTTGCGCGAGAAGATTGTGCACCGCCGCTTTACAGGCTTCGAC
GCCGCTTCGTTCTACCATCGACACCACCACGCTGGCACCCAGTTGATCGGCGCGA
GATTTAATCGCCGCGACAATTTGCGACGGCGCGTGCAGGGCCAGACTGGAGGTG
GCAACGCCAATCAGCAACGACTGTTTGCCCGCCAGTTGTTGTGCCACGCGGTTGG
GAATGTAATTCAGCTCCGCCATCGCCGCTTCCACTTTTTCCCGCGTTTTCGCAGAA
ACGTGGCTGGCCTGGTTCACCACGCGGGAAACGGTCTGATAAGAGACACCGGCA
TACTCTGCGACATCGTATAACGTTACTGGTTTCACATTCACCACCCTGAATTGACT
CTCTTCCGGGCGCTATCATGCCATACCGCGAAAGGTTTTGCACCATTCGATGGTG
TCCAATAACTGCCTTAAAAAAATTACGCCCCGCCCTGCCACTCATCGCAGTACTGT
TGTAATTCATTAAGCATTCTGCCGACATGGAAGCCATCACAGACGGCATGATGAA
CCTGAATCGCCAGCGGCATCAGCACCTTGTCGCCTTGCGTATAATATTTGCCCAT
GGTGAAAACGGGGGCGAAGAAGTTGTCCATATTGGCCACGTTTAAATCAAAACTG
GTGAAACTCACCCAGGGATTGGCTGAGACGAAAAACATATTCTCAATAAACCCTT
TAGGGAAATAGGCCAGGTTTTCACCGTAACACGCCACATCTTGCGAATATATGTG
TAGAAACTGCCGGAAATCGTCGTGGTATTCACTCCAGAGCGATGAAAACGTTTCA
GTTTGCTCATGGAAAACGGTGTAACAAGGGTGAACACTATCCCATATCACCAGCT
CACCGTCTTTCATTGCCATACGGAATTCCGGATGAGCATTCATCAGGCGGGCAAG
AATGTGAATAAAGGCCGGATAAAACTTGTGCTTATTTTTCTTTACGGTCTTTAAAA
AGGCCGTAATATCCAGCTGAACGGTCTGGTTATAGGTACATTGAGCAACTGACTG
AAATGCCTCAAAATGTTCTTTACGATGCCATTGGGATATATCAACGGTGGTATATC
CAGTGATTTTTTTCTCCATTTTAGCTTCCTTAGC**
